# Establishing new grid‐size‐dependent attributes to rank areas of endemism for conservation priorities

**DOI:** 10.1111/cla.70002

**Published:** 2025-06-19

**Authors:** Augusto Frota, Weferson Júnio da Graça

**Affiliations:** ^1^ Universidade Estadual de Maringá (UEM), Centro de Ciências Biológicas (CCB), Departamento de Biologia (DBI), Programa de Pós‐Graduação em Ecologia de Ambientes Aquáticos Continentais (PEA) Av. Colombo, 5790 Maringá 87020900 Paraná Brazil; ^2^ UEM, CCB, Programa de Pós‐Graduação em Biologia Comparada (PGB) Av. Colombo, 5790 Maringá 87020900 Paraná Brazil; ^3^ UEM, CCB, DBI and Núcleo de Pesquisas em Limnologia, Ictiologia e Aquicultura (Nupélia) Av. Colombo, 5790 Maringá 87020900 Paraná Brazil

## Abstract

Delineating Areas of Endemism (AEs) is crucial for identifying priority areas for biodiversity conservation in a spatial planning framework. Endemicity Analysis in the NDM/VNDM software is one of the primary methodologies for its delineation. Larger grid sizes have been employed to yield higher endemicity scores for AEs, recovering more endemic species and enhancing their conservation appeal. Compiling a robust geographic distribution dataset for 399 freshwater fish species from Southern Brazil, we identified AEs by conducting endemicity analyses with three different grid sizes. We also developed a spatial conservation Priority Index incorporating three grid‐size‐dependent attributes. We identified 153 AEs, each varying in endemicity scores, species richness, and threatened species. These variations were influenced by the analysed grid size and spatial overlap with specific freshwater ecoregions. The recovered AEs show freshwater fish distribution patterns corroborating significant vicariance events and faunal exchanges between river basins in distinct bordering ecoregions. We found an almost 50% reduction in spatial area when ranking Endemicity and Priority Index scores. This finding demonstrates the effectiveness of the Priority Index in highlighting similar sets of endemic, sympatric, and threatened species within smaller areas. This approach can effectively reconcile attributes easily extracted from the NDM/VNDM program when prioritizing spatial conservation.

## Introduction

Threats to biodiversity pose an urgent global challenge, and systematic conservation planning focuses on implementing cost‐effective actions to achieve biodiversity conservation goals (Margules and Pressey, 2000; Giakoumi et al., 2025). Future decision‐making will have long‐term effects on the planet and must be guided by effective, robust, and transparent spatial planning (Smith et al., 2022; Giakoumi et al., 2025). There is an urgent need for government agencies to provide national guidance and datasets to set targets that include species data in spatial planning, thus creating a national framework to inform local actions (Smith et al., 2022). While scientists can provide valuable guidance to decision‐makers, they have limitations in tackling complex spatial optimization problems (Martin et al., 2012). To address this issue, spatial conservation prioritization has emerged as a potential solution (Kukkala and Moilanen, 2013). This process involves identifying critical areas for biodiversity and determining the most effective ways and times to achieve conservation goals (Kukkala and Moilanen, 2013; Smith et al., 2022; Giakoumi et al., 2025). Prioritization is typically part of a broader systematic conservation planning framework (Margules and Pressey, 2000; Smith et al., 2022; Giakoumi et al., 2025), which employs one or more operational models to transform prioritization into actionable conservation strategies (Martin et al., 2012; Kukkala and Moilanen, 2013; Smith et al., 2022; Giakoumi et al., 2025).

Endemism plays a significant role in identifying priority areas for conservation through spatial prioritization (e.g. Darwall and Vié, 2005; Richardson and Whittaker, 2010; Gonçalves et al., 2018; Tognelli et al., 2019; Tumini et al., 2019; Dagosta et al., 2021; Frota et al., 2021; Della and Prado, 2024). Species that are geographically restricted often exhibit highly congruent distribution patterns, which support the identification of Areas of Endemism (hereafter AEs). Although there has been criticism regarding the potential artificiality and subjectivity of these areas (see Hovenkamp, 1997; Schultz and Cracraft, 2024), they can indicate intrinsic evolutionary histories (Morrone, 2009; Nogueira et al., 2010; Dagosta et al., 2021), encompassing both historical and ecological elements within a specific geographic space (Albert et al., 2020; Pacifico et al., 2020; Della and Prado, 2024). Moreover, information on endemic species can be particularly useful when studying regions that promote extensive speciation (e.g. Jézéquel et al., 2020; Dagosta et al., 2021; Frota et al., 2021).

There are several criteria in the literature for defining AEs. Most definitions emphasize the varying degrees of congruence in the distribution of species or taxa within a specific area, focusing primarily on the partial or total sympatry of taxa (Cracraft, 1985; Platnick, 1991; Morrone, 1994, 2009; Linder, 2001; Szumik et al., 2002; Szumik and Goloboff, 2004; Escalante, 2015). In general, recovered AEs rely mainly on the spatial overlap of the distributions of two or more species (Platnick, 1991; Linder, 2001; Szumik et al., 2002). Historically, AEs have been important to serve as precursors for geographic units used in cladistic biogeographic analyses. Thus, AEs form the foundation for constructing hypotheses regarding the biogeographic history of a taxon or the area it inhabits, as well as for proposing biogeographic regionalizations (Cracraft, 1985; Morrone, 1994, 2009; Linder, 2001; Szumik et al., 2002; Domínguez et al., 2006; Ebach et al., 2008; Munguía‐Lino et al., 2016). Also, AEs are high‐priority targets for conservation efforts because they contain unique and irreplaceable biotas with a shared evolutionary history (Cracraft, 1985; Myers et al., 2000; Szumik et al., 2002; Guedes et al., 2014; Morrone, 2014; Martínez‐Hernández et al., 2015; Gomes‐da‐Silva et al., 2017; Frota et al., 2021; Della and Prado, 2024).

One of the primary methodologies proposed for identifying AEs is Endemicity Analysis (Szumik et al., 2002; Szumik and Goloboff, 2004; Morrone, 2018). This analysis utilizes the NDM/VNDM endemism viewer program (Goloboff, 2024) to pinpoint areas with exclusive species and evaluate the spatial congruence of different taxa. It uses a series of grids over the study area in conjunction with the distribution of georeferenced occurrence data (Szumik et al., 2002; Szumik and Goloboff, 2004). Since grids serve as sampling units, the analyses are scale‐dependent. Smaller grids are employed to identify areas with spatial concordance of species distributions in localized regions, typically featuring local endemics. Conversely, larger grids are necessary for detecting regions with more widely distributed species (e.g. Aagesen et al., 2009; Casagranda et al., 2009; DaSilva et al., 2015; Prado et al., 2015; Ocampo‐Salinas et al., 2019; Frota et al., 2021).

For conservation purposes, AEs are prioritized based on their endemicity scores, often using larger grid sizes, as these can capture a greater concentration of endemic species (e.g. Frota et al., 2021). However, due to practical and financial constraints, it is necessary to prioritize some regions over others, as it is not feasible to protect every population of all species everywhere (Kukkala and Moilanen, 2013). To enhance conservation efforts, it may be beneficial to combine other attributes that depend on grid size and are relevant to AEs. This approach could help develop indices that optimize cost–benefit ratios, guiding the prioritization of conservation efforts within the spatial conservation prioritization framework (Martin et al., 2012; Kukkala and Moilanen, 2013).

Effective prioritization for biodiversity conservation requires a strong understanding of biodiversity and its geographic distribution. In Brazil, many national parks and areas designated for biodiversity conservation fail to achieve their intended goals, particularly in aquatic environments and their diverse fish populations (Azevedo‐Santos et al., 2019; Bailly et al., 2021; Dagosta et al., 2021; Frota et al., 2021). Historically, aquatic organisms have often been overlooked in the establishment of conservation areas due to a lack of sufficient data on their taxonomy and geographic distributions (Heilpern, 2015; Dagosta et al., 2021, 2024). Therefore, understanding the distribution patterns of freshwater fish is essential for developing effective strategies aimed at conserving aquatic and endemic biodiversity (Chakona et al., 2019; Tognelli et al., 2019; Dagosta et al., 2021; Frota et al., 2021). While priority regions for the conservation of freshwater fish have been identified on both global (e.g. Abell et al., 2008; Tedesco et al., 2017) and continental scales (e.g. Reis et al., 2016; Jézéquel et al., 2020; Dagosta et al., 2021), such data remain limited at the local scale within Brazil (e.g. Nogueira et al., 2010; Frota et al., 2021). This study aims to: (i) identify AEs using the NDM/VNDM program, utilizing robust data on freshwater fish occurrences in Southern Brazil; (ii) establish biogeographic patterns by analysing variations across three different grid sizes (0.1° × 0.1°, 0.3° × 0.3°, and 0.5° × 0.5°); and (iii) propose a spatial conservation Priority Index for the identified AEs, taking into account grid‐size‐dependent attributes such as species richness per area (i.e. sympatric species), endemic species per area, and threatened species per area.

## Material and methods

### Study area

**Fig. 1:**
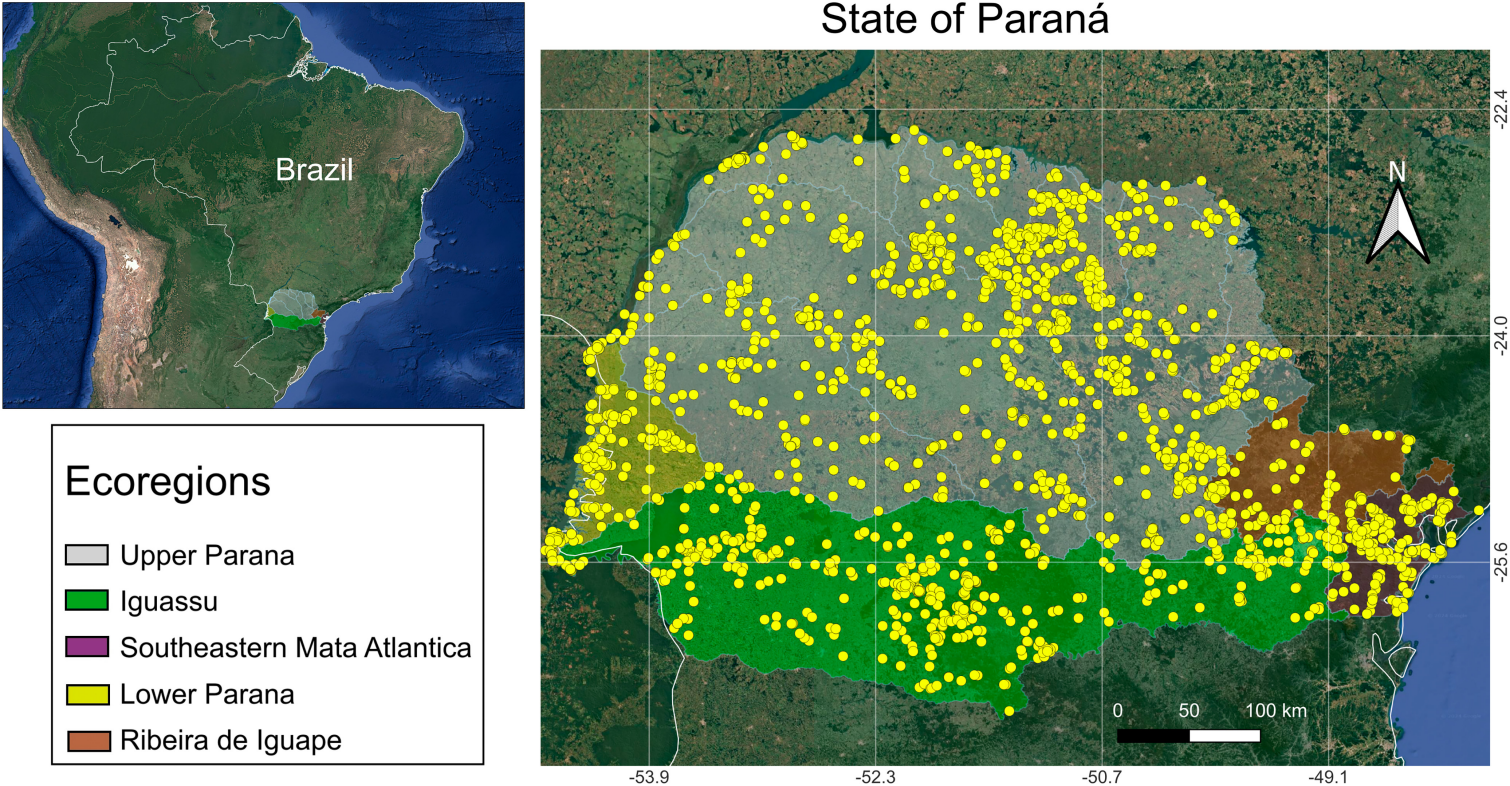
Map of the study area, highlighting the State of Paraná in Southern Brazil. The main river basins in the state are colour‐coded to represent the five freshwater ecoregions, as categorized by Reis et al. (2020). The yellow dots on the map indicate the georeferenced records used to compile the analysed database.

The study area encompasses the river basins of the State of Paraná, located in Southern Brazil (see Fig. 1). This state is bordered by the State of São Paulo to the North, Paraguay and Argentina to the West, State of Santa Catarina to the South, and the Atlantic Ocean to the East. The State of Paraná consists of 399 municipalities and covers an area of 196490.1 km^2^ (Paraná, 2010). According to the IBGE (2022), the population is approximately 11 444 380 inhabitants.

The State of Paraná comprises five freshwater biogeographic regions (ecoregions, according to Abell et al., 2008), although none of these regions are entirely contained within its boundaries (Reis et al., 2020). The river basins in the State of Paraná (Fig. 1) are divided into two main slopes. The Atlantic slope includes drainages that flow eastward directly into the Atlantic Ocean. In contrast, the Interior slope consists of drainages that flow westward and is completely covered by the Paraná River basin, which is part of the Río de La Plata system. The Paraná River itself extends far south of the State of Paraná, eventually reaching the estuary of the Río de La Plata in Argentina and Uruguay. This Interior slope also includes sections of the Upper Paraná, Lower Paraná, and Iguassu ecoregions (Abell et al., 2008; Reis et al., 2020).

The Interior slope covers an area of 183 225 km^2^, which corresponds to approximately 90% of the state's total area (Paraná, 2010). The Upper Parana ecoregion in the State of Paraná is divided into four distinct sub‐ecoregions, each characterized by significantly different fish communities (Reis et al., 2020). The Ivaí and Piquiri subecoregions are associated with unique river basins that are entirely located within the state. The Paranapanema subecoregion includes the Paranapanema River and its tributaries, which extend partially into the State of São Paulo. Lastly, the Floodplain subecoregion is made up of several small tributaries that flow directly into the Paraná River. The Paraná River floodplain primarily lies along its right bank, within the State of Mato Grosso do Sul; however, the tributaries of the Floodplain subecoregion in the State of Paraná share many species with the floodplain, being strongly influenced by it (see Ota et al., 2018).

The concept of the Lower Parana ecoregion described by Reis et al. (2020) differs slightly from that presented by Abell et al. (2008). In the latter, the northern boundary is approximately at the Itaipu Dam; however, according to Reis et al. (2020), the Itaipu reservoir and its tributaries are included within the Lower Parana ecoregion. This change reflects the original biogeographic barrier that separates it from the Upper Parana ecoregion, namely the Sete Quedas waterfalls, which are now submerged (see Júlio Júnior et al., 2009). Additionally, the Iguassu ecoregion encompasses the entire extent of the Iguassu River basin, except for a small section downstream of the Iguassu Falls, which is classified as part of the Lower Parana ecoregion (Reis et al., 2020).

The Atlantic slope encompasses part of the Ribeira de Iguape River basin, which is a key component of the Ribeira de Iguape ecoregion. Additionally, it includes several smaller coastal drainages that flow into Guaratuba Bay and Paranaguá Bay, both of which are part of the Southeastern Mata Atlantica ecoregion (Abell et al., 2008; Reis et al., 2020). The Ribeira de Iguape River basin covers an area of 28 306 km^2^, with 9736 km^2^ located in the State of Paraná, representing approximately 5% of the state's total area (Paraná, 2010). The portion of the Southeastern Mata Atlantica ecoregion within the State of Paraná extends over 5630.8 km^2^, which accounts for about 3% of the state's total area. In the state, the Atlantic slope contains the largest proportion of remaining forest cover, totaling 81%. This high percentage is largely due to the dominance of mountainous and steep terrain in the region (Paraná, 2010; IPARDES, 2017).

The State of Paraná is primarily composed of magmatic and metamorphic rocks situated on the South American Platform. This platform serves as the foundation for the sedimentary and volcanic units of the Paraná Shield (Maack, 2017). The Paraná Shield consists mainly of crystalline, igneous, and metamorphic rocks, representing the oldest and highest regions of the state. The eastern area where the Paraná Shield is exposed is referred to as the First Paranaense Plateau (Maack, 2017). The western parts of the First Paranaense Plateau are predominantly made up of volcanic and Palaeozoic sedimentary rocks, collectively known as the Paraná Basin (Maack, 2017). The Paraná Basin includes two geological units: the Second Paranaense Plateau and the Third Paranaense Plateau. These units cover most of the state, and their boundaries were defined by a flexural crystalline uplift known as the Ponta Grossa Arch (Franco‐Magalhães et al., 2010; Maack, 2017).

### Database preparation

The compilation of georeferenced records by ecoregion for native freshwater fish species in the State of Paraná has been updated from Reis et al. (2020). This update incorporates the most recent records available from various ichthyological collections up to November 2024, including: California Academy of Sciences, San Francisco, USA (CAS); Coleção de Peixes da Universidade Federal do Rio Grande do Sul, Porto Alegre, Brazil (UFRGS); Coleção de Peixes do Laboratório de Ictiologia de Ribeirão Preto, Universidade de São Paulo, Ribeirão Preto, Brazil (LIRP); Coleção Ictiológica do GERPEL, Toledo, Brazil (CIG); Coleção Ictiológica do NUPÉLIA, Maringá, Brazil (NUP); Field Museum of Natural History, Chicago, USA (FMNH); Museu de Ciências e Tecnologia da PUCRS, Porto Alegre, Brazil (MCP); Museu de História Natural Capão da Imbuia, Curitiba, Brazil (MHNCI); Museu de Zoologia da Universidade Estadual de Londrina, Londrina, Brazil (MZUEL); Museu de Zoologia da Universidade de São Paulo, São Paulo, Brazil (MZUSP); Museu Nacional, Universidade Federal do Rio de Janeiro, Rio de Janeiro, Brazil (MNRJ); and Museu Paraense Emílio Goeldi, Belém, Brazil (MPEG). These records can be accessed through SpeciesLink (http://splink.cria.org.br), FishNet2 (http://fishnet2.net), and SiBBr (https://collectory.sibbr.gov.br/collectory/public/show/dr186). All records obtained through virtual searches in these databases have been verified against primary sources of information, including original descriptions, taxonomic revisions, inventories, and notes on geographic distribution.

Additionally, to address sampling gaps in several river basins in the State of Paraná, eight field trips were conducted on the following dates: January 2014, October 2014, October 2015, June 2017, July 2018, October 2023, April 2024, and October 2024. These trips resulted in the sampling of 261 rivers and streams of Strahler order less than three (Strahler, 1957) from various basins, including the Ivaí, Piquiri, Tibagi, das Cinzas, and Itararé (Upper Parana ecoregion); São Francisco Falso and São Francisco Verdadeiro (Lower Parana ecoregion); Ribeira de Iguape (Ribeira de Iguape ecoregion); as well as Jordão, Areia, Chopim, Iratim, and other smaller rivers (Iguassu ecoregion). At each sampling site, a 100‐meter stretch containing at least one sequence of riffle‐run‐pool mesohabitats was defined, georeferenced, and sampled using electrofishing. This technique utilizes two electrified dip nets that generate an electric discharge of 200–400 volts and a current of 2 amperes to temporarily stun the fish (Alves et al., 2021). All captured specimens were first anaesthetised with benzocaine hydrochloride and then fixed with 10% formalin in the field. After a few days, the fish were transferred to 70% ethanol in the laboratory and deposited in the Coleção Ictiológica do NUPÉLIA (NUP). Voucher specimens and sampling species were documented in previously published studies (Frota et al., 2016a, b, 2019, 2020, 2021; Cavalli et al., 2018; Reis et al., 2020; Mezzaroba et al., 2021).

Taxon identifications were updated for the Iguassu ecoregion according to Baumgartner et al. (2012) and Mezzaroba et al. (2021). For the Ivaí River basin in the Upper Parana ecoregion, updates were made based on the studies of Frota et al. (2016a), Reis et al. (2020), and Dagosta et al. (2024). The Piquiri River basin, also in the Upper Parana ecoregion, was referenced using Cavalli et al. (2018), Reis et al. (2020), and Dagosta et al. (2024). Jarduli et al. (2020), Reis et al. (2020), and Dagosta et al. (2024) provided updates for the das Cinzas, Itararé, and Tibagi river basins, all in the Upper Parana ecoregion. For the Atlantic slope basins, including Ribeira de Iguape and Southeastern Mata Atlantica ecoregions, the updates were based on Oyakawa et al. (2006), Frota et al. (2019), and Reis et al. (2020). The validity of the species names was verified in Fricke et al. (2025). The final database contains 17 538 georeferenced records for 399 native fish species from the State of Paraná and is available in the Supporting Information.

This study received approval from the Committee of Ethics for the Use of Animals in Experimentation at the State University of Maringá (UEM), under process numbers 5680160117 and 7283090823. Specimens were collected with permanent permit number 14028 issued by the Brazilian Institute of Environment and Renewable Natural Resources (IBAMA).

### Biogeographic analysis

Endemicity Analysis, as described by Szumik et al. (2002) and Szumik and Goloboff (2004), was conducted to identify AEs using the NDM/VNDM software (Goloboff, 2024). This approach measures the congruence between the distribution of a species and a specific area using the Endemicity Index (EI), which ranges from zero to one. The method categorizes species records into three groups: absent, present, and presumably present. The EI increases when a species is recorded in more grids within a given area and decreases when the same species is found in grids outside that area. Therefore, the EI equals one for species that are uniformly distributed solely within that area and declines for species that are also found elsewhere or are unevenly distributed within the area. The analysis provides options for “radius size to fill” and “radius size to assume”, that correspond to inferred and assumed presences, respectively. These options allow users to add a percentage of the cell size for each parameter, promoting a major impact on the EI (see Szumik and Goloboff, 2004; Della and Prado, 2024). More detailed mathematical expressions and calculations for the EI can be found in the updated method by Szumik and Goloboff (2004).

NDM/VNDM generates grids within the area being studied, each with an endemicity value. The final Endemicity score (E) of an Area of Endemism (AE) is calculated by summing the Endemicity Indices (EIs) of the endemic species present in that area (Szumik and Goloboff, 2004). Two key factors contribute to the final E score: the number of species included in the area and the degree of congruence, as measured by the EI, between the distributions of these species and the area itself (Szumik and Goloboff, 2004). As a result, the final E score increases when both the number of species in the area and the alignment between these species and the area improve (Szumik and Goloboff, 2004; Aagesen et al., 2013). Depending on the objectives and scale of the analysis, the resulting AEs can be combined into consensus areas (hereafter CAs) using either strict or flexible criteria (see Aagesen et al., 2013; Szumik and Goloboff, 2015).

A spreadsheet containing the georeferenced records of the evaluated fish species was saved as a CSV file and converted to an XYD file using the online tool GeX (http://gex.mfuhlendorf.com/) (Santos and Fuhlendorf, 2019). Analyses were conducted using three different grid sizes (0.1° × 0.1°, 0.3° × 0.3°, and 0.5° × 0.5°) with grid origin entered for X = −55.0 and Y = −22.0. Only sets of grids that included two or more endemic species were retained if they had a final E score of at least two. For each grid size, the search was performed with 100 replicates. In the software, default values for the factors involved in the calculation of the EI were used (see Szumik and Goloboff, 2004). No values were added for “radius size to fill” and “radius size to assume”. This procedure was chosen because we could assume records that do not align with the freshwater biogeographic patterns observed at the boundaries of the river basins, particularly in the ecoregions adjacent to the study area, for all grid sizes analysed. Accordingly, by using default parameters, the observed occurrences have a higher value than the assumed occurrences in the analysis (Szumik and Goloboff, 2004; Hoffmeister and Ferrari, 2016). To delineate the gradual overlap and replacement of species between candidate areas, CAs were obtained using a flexible consensus rule with a cut‐off value of 10% (see Aagesen et al., 2013; DaSilva et al., 2015). Area information was exported from NDM/VNDM and converted to shapefiles using DIVAGIS (Hijmans et al., 2001; see also Prado et al., 2015).

### Priority Index

For each AE recovered in all analyses, we plotted the following values: final E scores, ichthyofaunal units (i.e. ecoregions) that overlap with the area, the number of species with EI score (i.e. the number of endemic species in the area), grid size, the number of grids in the area, the number of species occurring in the area (i.e. sympatric species), and the number of threatened species. Most of these values are provided directly by the NDM/VNDM program; however, the categorization of threatened species was based on State Decree No. 6040 (Paraná, 2024), National Ordinance N^o^. 148 (MMA, 2022), or the Biodiversity Extinction Risk Assessment System (ICMBio, 2025).

Using these values, we calculated the Priority Index score (PI) for each area and ranked them from highest to lowest priority based on specific combinations of overlapping ecoregion(s) (e.g. Lower Parana + Iguassu; Ribeira de Iguape + Upper Parana + Iguassu). The Priority Index for each AE recovered across all analysed grid sizes was determined using three specific attributes: (I) Species/Area, (II) Endemicity, and (III) Extinction.

The Species‐Area attribute (*A*) was calculated by the expression:

$$A=a/\left(b\times c\right),$$

where *a* = number of species occurring in the area; *b* = number of grids in the area; *c* = multiplication factor for grid size (0.1° = 1, 0.3° = 9 and 0.5° = 25); *b* × *c* = equivalent grid (this takes into account the effect of grid size through the multiplication factor, as 0.3° × 0.3° and 0.5° × 0.5° grids are, respectively, nine and 25 times larger in area than 0.1° × 0.1° grids).

The Endemicity attribute (*B*) was calculated by the expression:

$$B=\left(d\times e\times f\right)/\left(b\times c\right),$$

where *d* = number of species with EI score (i.e. endemic species); *e* = final E score of the area; *f* = ichthyofaunal units (ecoregions) overlapping with the area; *b* × *c* = equivalent grid as previously described.

The Extinction attribute (C) was calculated by the expression:

$$C=\left(2^{g}\times 3^{h}\times 4^{i}\times 5^{j}\times 6^{k}\right)/\left(b\times c\right),$$

where *g* = total number of species in the area categorized as Near Threatened (NT); *h* = total number of species in the area categorized as Vulnerable (VU); *i* = total number of species in the area categorized as Endangered (EN); *j* = total number of species in the area categorized as Critically Endangered (CR); *k* = total number of species in the area categorized as Probably Extinct in the Wild (CR PEX); *b* × *c* = equivalent grid as previously described.

The final PI score was calculated by the expression:

PI=logA×B×C.

Finally, the AEs for each combination of ecoregion(s) were ranked based on their Endemicity (*E*) and Priority Index (PI) scores. This ranking was then used to create maps that visualize the spatial differences in conservation priorities for areas with similar sets of endemic, sympatric, and threatened species. All maps were generated using QGIS 2.14 software.

## Results

### Recovered areas of endemism

**Fig. 2:**
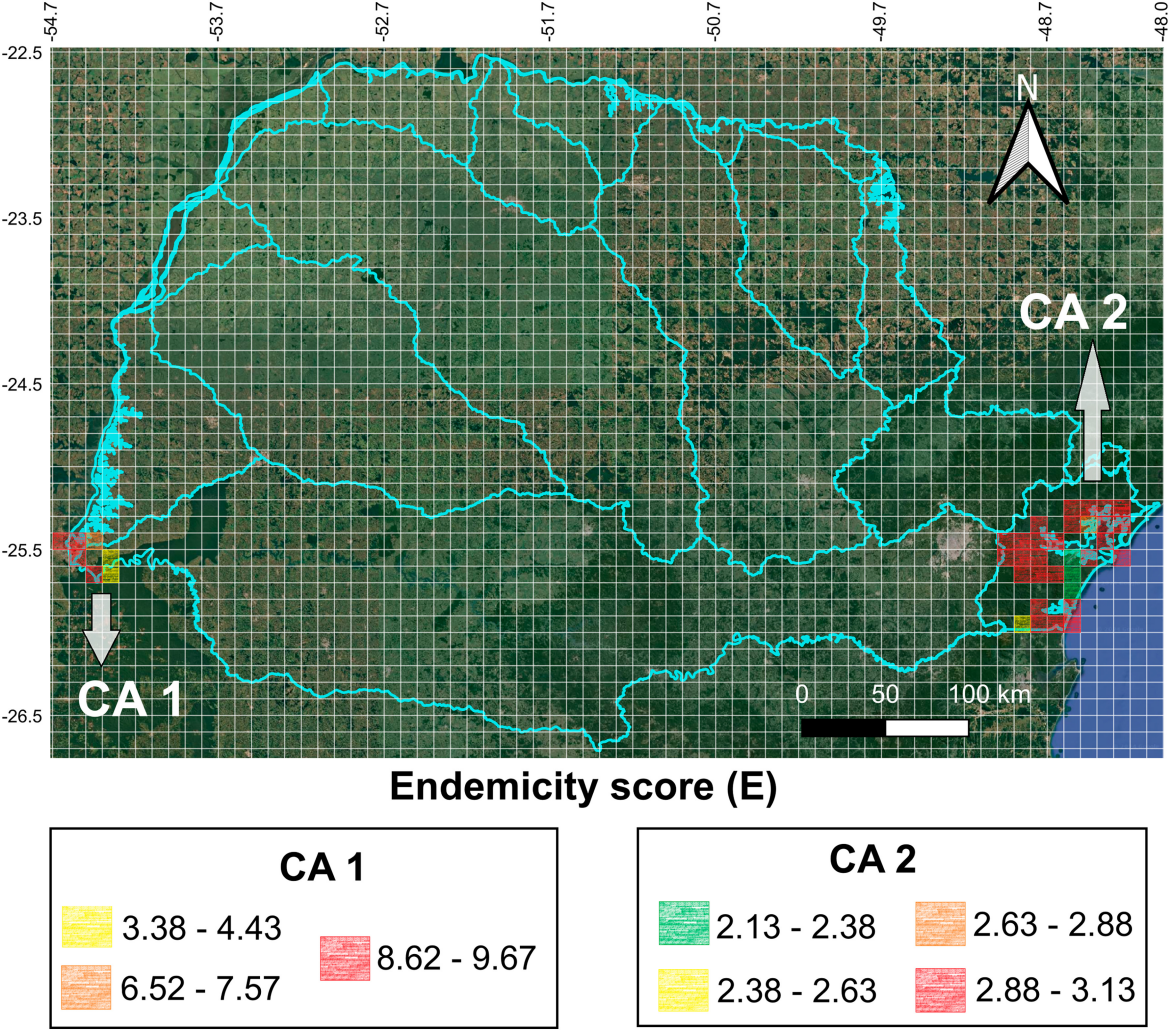
Consensus Areas (CAs) and their corresponding ranges of Endemicity scores (E) obtained through Endemicity Analysis for grid sizes of 0.1° × 0.1°.

In the Endemicity Analysis performed with a grid size of 0.1° × 0.1°, 17 AEs were recovered (Table S1; Figs. S1–S17). The *E* score of each area ranged from 2.13 to 9.67 (Table S1), the size/number of grids ranged from 2 to 24 (Table S1), the number of endemic species ranged from 3 to 12 species (Table S1), the number of sympatric species ranged from 61 to 140 species (Table S1), and three combinations of ecoregion(s) were detected: Lower Parana; Lower Parana + Iguassu; and Southeastern Mata Atlantica (Table 1). In the consensus analysis, the 17 AEs were grouped into two CAs (Table 1; Fig. 2). The combinations of ecoregion(s) were arranged in Lower Parana and Lower Parana + Iguassu in CA 1 (Fig. 2), with 14 endemic species (Table 1) and in Southeastern Mata Atlantica in CA 2 (Fig. 2), with nine endemic species (Table 1).

**Table 1:** Results of endemicity analysis for a 0.1° × 0.1° grid size

CAs	AEs	Ecoregion(s)	*N*	Endemic species (EI)	Size	*E*
1	2, 7, 8 12, 13, 14, 15, and 16	Lower Parana/Lower Parana + Iguassu	14	*Crenicichla mandelburgeri* (0.58), *Hemiodus orthonops* (0.88), *Bryconamericus exodon* (1.0), *Saxatilia lepidota* (1.0), *Gymnogeophagus setequedas* (0.88), *Odontostilbe pequira* (1.0), *Bujurquina vittata* (1.0), *Platydoras armatulus* (0.88), *Pachyurus bonariensis* (1.0), *Plagioscion squamosissimus* (1.0), *Paravandellia oxyptera* (1.0), *Abramites hypselonotus* (1.0), *Moenkhausia dichroura* (1.0), and *Triportheus nematurus* (1.0)	7	2.33–9.67
2	1, 3, 4, 5, 6, 9, 10, 11, and 17	Southeastern Mata Atlantica	9	*Acentronichthys leptos* (0.74), *Hisonotus leucofrenatus* (0.84), *Hyphessobrycon griemi* (0.49), *Phalloceros pellos* (0.77), *Microglanis cottoides* (0.68), *Scleromystax macropterus* (0.65), *Schizolecis guntheri* (0.74), *Gymnotus pantherinus* (0.83), and *Crenicichla lacustris* (0.63)	31	2.13–2.98

CAs = consensus areas; AEs = included areas of endemism in each CA (coding according to Table S1 and Figs. S1–S17); *N* = Number of endemic species in each CA; EI = highest individual value of the Endemicity Index; Size = number of grids of each CA; E = minimum and maximum final Endemicity scores of each CA.

**Fig. 3:**
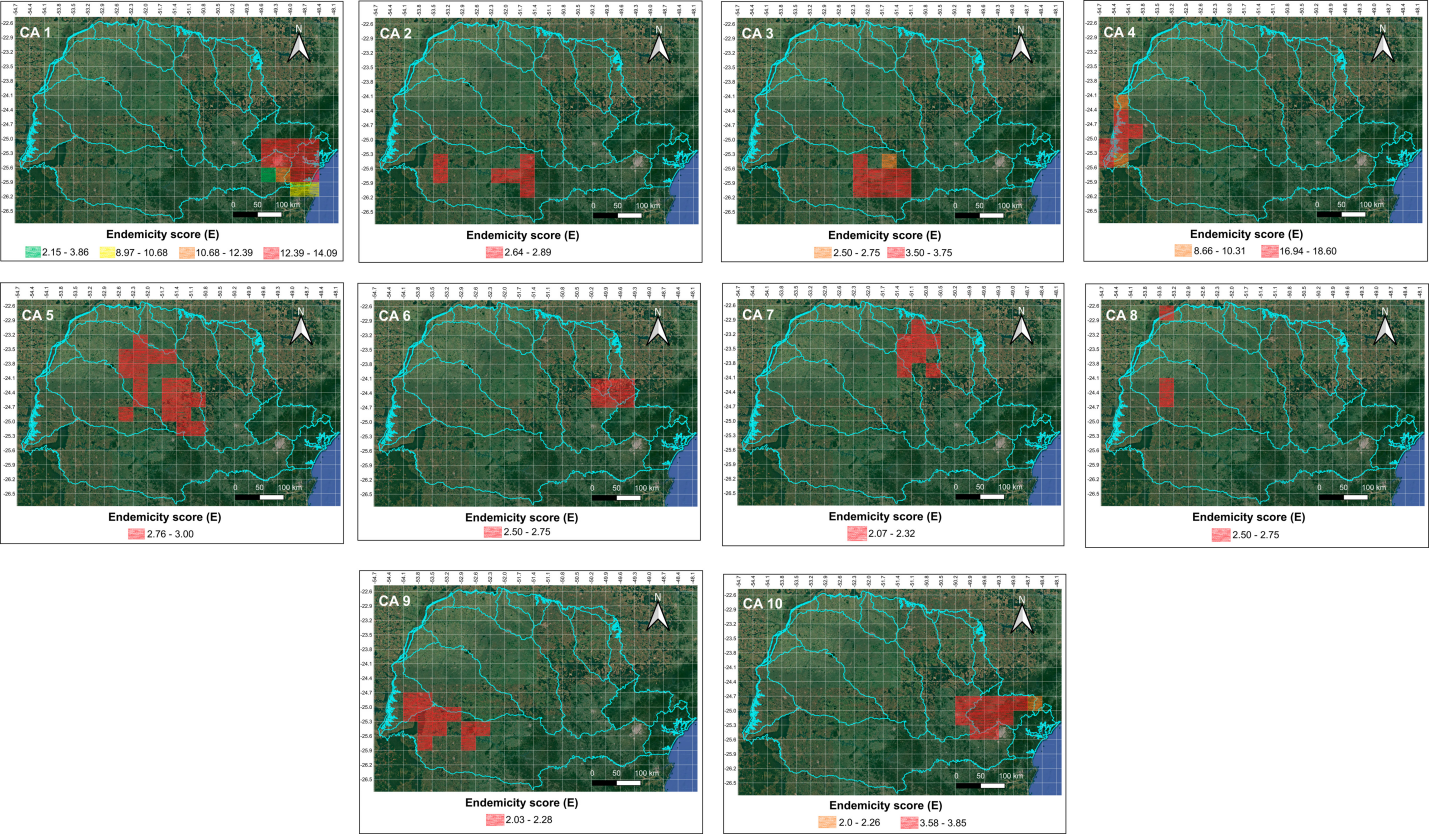
Consensus Areas (CAs) and their corresponding ranges of Endemicity scores (E) obtained through Endemicity Analysis for grid sizes of 0.3° × 0.3°.

In the Endemicity Analysis performed with a grid size of 0.3° × 0.3°, 45 AEs were recovered (Table S2; Figs. S18–S62). The E score of each area ranged from 2.0 to 18.6 (Table S2), the size/number of grids ranged from 2 to 16 (Table S2), the number of endemic species ranged from 2 to 28 species (Table S2), the number of sympatric species ranged from 33 to 154 species (Table S2) and 11 combinations of ecoregion(s) were detected: Lower Parana; Iguassu; Upper Parana; Southeastern Mata Atlantica; Lower Parana + Iguassu; Lower Parana + Upper Parana + Iguassu, Ribeira de Iguape + Upper Parana; Ribeira de Iguape + vUpper Parana + Iguassu; Southeastern Mata Atlantica + Iguassu; Southeastern Mata Atlantica + Ribeira de Iguape + Iguassu; and Upper Parana + Iguassu (Table 2). In the consensus analysis, the 45 AEs were grouped into 10 CAs (Table 2; Fig. 3). The combinations of ecoregion(s) were arranged as Southeastern Mata Atlantica; Southeastern Mata Atlantica + Iguassu; and Southeastern Mata Atlantica + Ribeira de Iguape + Iguassu in CA 1 (Fig. 3), with 29 endemic species (Table 2); in Iguassu in CA 2 (Fig. 3), with four endemic species (Table 2); in Iguassu and Upper Parana + Iguassu in CA 3 (Fig. 3), with nine endemic species (Table 2); in Lower Parana; Lower Parana + Iguassu; and Lower Parana + Upper Parana + Iguassu in CA 4 (Fig. 3), with 39 endemic species (Table 2); in Upper Parana in CA 5 (Fig. 3), with six endemic species (Table 2); in Ribeira de Iguape + Upper Parana in CA 6 (Fig. 3), with four endemic species (Table 2); in Upper Parana in CA 7 (Fig. 3), with four endemic species (Table 2); in Upper Parana in CA 8 (Fig. 3), with three endemic species (Table 2); in Lower Parana + Iguassu and Lower Parana + Upper Parana + Iguassu in CA 9 (Fig. 3), with six endemic species (Table 2); and in Ribeira de Iguape + Upper Parana and Ribeira de Iguape + Upper Parana + Iguassu in CA 10 (Fig. 3), with 12 endemic species (Table 2).

**Table 2:** Results of endemicity analysis for a 0.3° × 0.3° grid size

CAs	AEs	Ecoregion(s)	*N*	Endemic species (EI)	Size	*E*
1	2, 6, 10, 11, 13, 17, 18, 27, 29, 32, 33, 36, 42, and 44	Southeastern Mata Atlantica/Southeastern Mata Atlantica + Iguassu/Southeastern Mata Atlantica + Ribeira de Iguape + Iguassu	29	*Deuterodon langei* (0.59), *Acentronichthys leptos* (1.0), *Hyphessobrycon bifasciatus* (0.68), *Hisonotus leucofrenatus* (0.75), *Scleromystax barbatus* (0.70), *Hollandichthys multifasciatus* (0.64), *Hyphessobrycon griemi* (0.90), *Phalloceros pelos* (0.88), *Mimagoniates lateralis* (1.0), *Poecilia vivípara* (1.0), *Microglanis cottoides* (0.65), *Scleromystax macropterus* (1.0), *Schizolecis guntheri* (1.0), *Ancistrus multispinis* (0.57), *Pareiorhaphis azygolechis* (0.63), *Spintherobolus ankoseion* (1.0), *Glandulocauda caerulea* (0.42), *Gymnotus pantherinus* (0.70), *Crenicichla lacustris* (0.75), *Rachoviscus crassiceps* (0.70), *Rhamdioglanis frenatus* (0.46), *Crenicichla tingui* (0.67), *Phalloceros alessandrae* (1.0), *Phalloceros buckupi* (0.70), *Atlantirivulus luelingi* (0.75), *Homodiaetus graciosa* (0.67), *Australoheros* sp. (0.61), *Listrura boticário* (0.67), and *Phalloptychus januarius* (1.0)	14	2.15–14.1
2	4	Iguassu	4	*Psalidodon gymnogenys* (0.38), *Ancistrus agostinhoi* (0.79), *Jenynsia diphyes* (0.61), and *Neoplecostomus* sp. 1 (0.86)	7	2.64
3	5, 7, 20, 21, 35, and 37	Iguassu/Upper Parana + Iguassu	9	*Astyanax jordanensis* (0.55), *Cambeva igobi* (0.55), *Cambeva crassicaudata* (1.0), *Cambeva papillifera* (0.75), *Cambeva cauim* (0.75), *Cambeva plumbea* (0.46), *Hypostomus nigropunctatus* (0.70), *Cambeva taroba* (1.0), and *Cnesterodon omorgmatos* (1.0)	10	2.25–3.75
4	8, 12, 24, 26, 38, 39, and 41	Lower Parana/Lower Parana + Iguassu/Lower Parana + Upper Parana + Iguassu	39	*Diapoma guarani* (1.0), *Catathyridium jenynsii* (0.39), *Loricariichthys rostratus* (0.50), *Crenicichla mandelburgeri* (1.0), *Roeboides descalvadensis* (0.57), *Hemiodus orthonops* (1.0), *Saxatilia lepidota* (1.0), *Tetragonopterus argenteus* (0.80), *Gymnogeophagus setequedas* (0.50), *Hypophthalmus oremaculatus* (0.67), *Loricariichthys platymetopon* (1.0), *Pimelodella taenioptera* (0.83), *Ageneiosus ucayalensis* (0.88), *Auchenipterus osteomystax* (0.64), *Serrasalmus marginatus* (0.64), *Cynopotamus kincaidi* (0.90), *Pterodoras granulosus* (0.80), *Trachelyopterus galeatus* (1.0), *Bujurquina vittata* (1.0), *Hypostomus cochliodon* (0.80), *Megaleporinus macrocephalus* (0.58), *Otocinclus vittatus* (0.70), *Platydoras armatulus* (1.0), *Trachydoras paraguayensis* (0.80), *Ageneiosus inermis* (1.0), *Moenkhausia forestii* (1.0), *Pimelodus mysteriosus* (0.50), *Pimelodus ornatos* (0.88), *Sorubim lima* (0.57), *Aphyocharax dentatus* (0.75), *Galeocharax humeralis* (0.75), *Ossancora eigenmanni* (0.80), *Paravandellia oxyptera* (1.0), *Psellogrammus kennedyi* (1.0), *Rhamphichthys hahni* (1.0), *Farlowella hahni* (1.0), *Platanichthys platana* (0.70), *Pterygoplichthys ambrosettii* (1.0), and *Triportheus nematurus* (1.0)	8	7.0–18.6
5	14, and 19	Upper Parana	6	*Hisonotus pachysarkos* (0.80), *Apareiodon vladii* (0.65), *Hypostomus robertsoni* (0.81), *Cambeva horacioi* (0.78), *Characidium heirmostigmata* (0.62), and *Cyphocharax* cf. *corumbae* (0.63)	19	2.76–2.85
6	16, and 34	Ribeira de Iguape + Upper Parana	4	*Characidium schubarti* (1.0), *Astyanax* sp. 3 (0.55), *Characidium itarare* (0.75), and *Cambeva pascuali* (0.50)	6	2.0–2.63
7	22	Upper Parana	4	*Schizodon intermedius* (0.46), *Cheirodon stenodon* (0.36), *Hisonotus depressicauda* (0.75), and *Bunocephalus larai* (0.50)	8	2.07
8	23, and 40	Upper Parana	3	*Amaralia oviraptor* (0.83), *Myloplus tiete* (1.0), *and Trachelyopterus* sp. (1.0)	3	2.0–2.5
9	28, 30, and 31	Lower Parana + Iguassu/Lower Parana + Upper Parana + Iguassu	6	*Tatia jaracatia* (0.86), *Bryconamericus pyahu* (0.81), *Crenicichla tuca* (0.79), *Hisonotus yasi* (0.79), *Australoheros angiru* (0.46), and *Gymnogeophagus taroba* (0.71)	12	2.03–2.18
10	1, 3, 9, 15, 25, 43, and 45	Ribeira de Iguape + Upper Parana/Ribeira de Iguape + Upper Parana + Iguassu	12	*Isbrueckerichthys duseni* (0.68), *Harttia kronei* (0.63), *Hypostomus interruptus* (0.57), *Chasmocranus lopezae* (0.80), *Neoplecostomus ribeirensis* (0.69), *Rhamdioglanis transfasciatus* (0.63), *Cambeva zonata* (0.90), *Astyanax* sp. 1 (1.0), *Ituglanis proops* (0.80), *Kronichthys subterres* (0.50), *Astyanax* sp. 2 (1.0), and *Isbrueckerichthys epakmos* (0.60)	12	2.0–3.85

CAs = consensus areas; AEs = included areas of endemism in each CA (coding according to Table S2 and Figs. S18–S62); *N* = Number of endemic species in each CA; EI = highest individual value of the Endemicity Index; Size = number of grids of each CA; E = minimum and maximum final Endemicity scores of each CA.

**Fig. 4:**
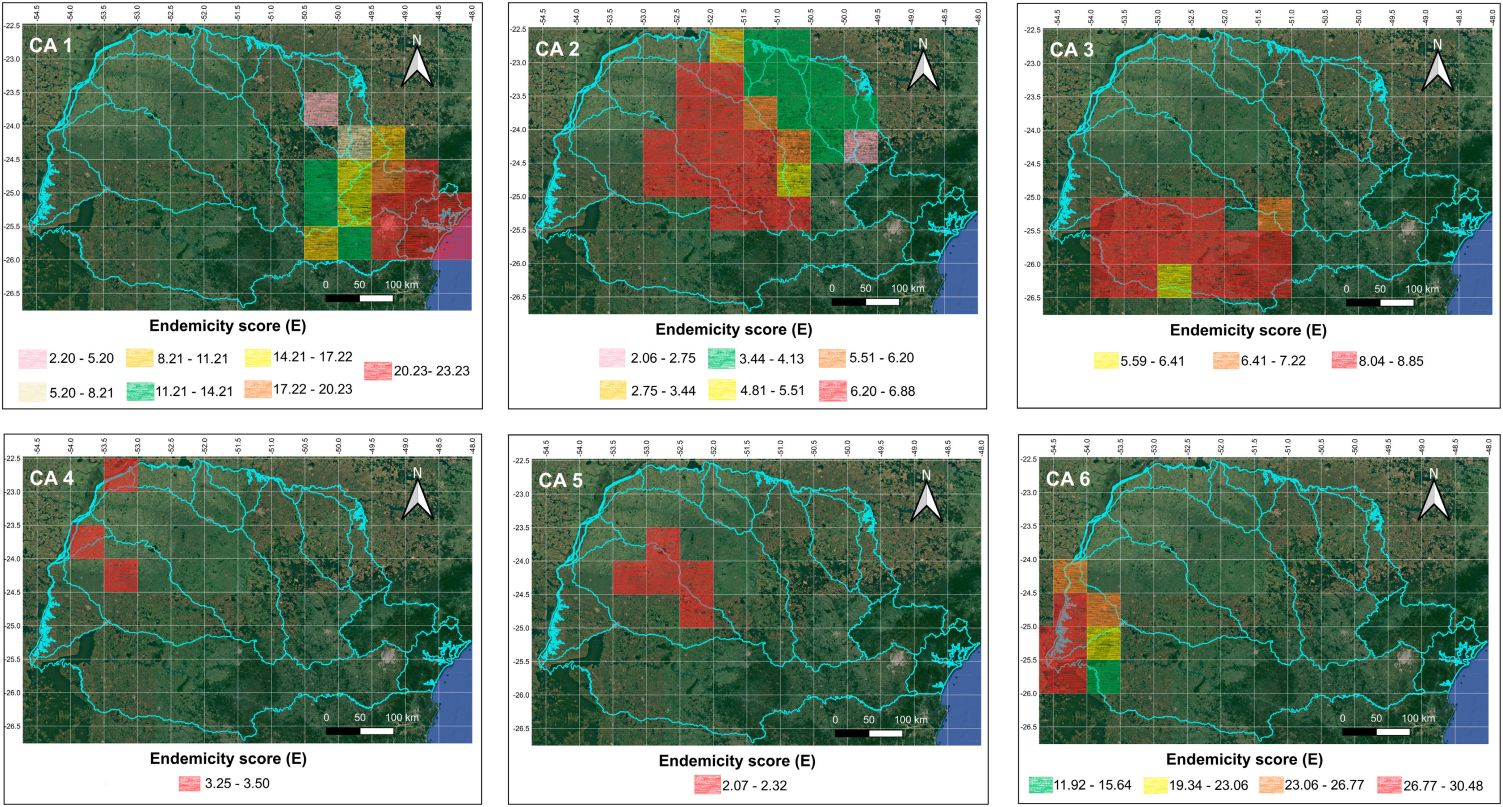
Consensus Areas (CAs) and their corresponding ranges of Endemicity scores (E) obtained through Endemicity Analysis for grid sizes of 0.5° × 0.5°.

In the Endemicity Analysis performed with a grid size of 0.5° × 0.5°, 91 AEs were recovered (Table S3; Figs. S63–S153). The E score of each area ranged from 2.06 to 30.49 (Table S3), the size/number of grids ranged from 2 to 14 (Table S3), the number of endemic species ranged from 3 to 41 species (Table S3), the number of sympatric species ranged from 32 to 172 species (Table S3) and 12 combinations of ecoregion(s) were detected: Lower Parana; Iguassu; Upper Parana; Southeastern Mata Atlantica; Lower Parana + Iguassu; Lower Parana + Upper Parana + Iguassu; Ribeira de Iguape + Upper Parana; Ribeira de Iguape + Upper Parana + Iguassu; Southeastern Mata Atlantica + Iguassu; Southeastern Mata Atlantica + Ribeira de Iguape + Iguassu; Southeastern Mata Atlantica + Ribeira de Iguape + Upper Parana + Iguassu; and Upper Parana + Iguassu (Table 3). In the consensus analysis, the 91 AEs were grouped into six CAs (Table 3; Fig. 4). The combinations of ecoregion(s) were arranged as Ribeira de Iguape + Upper Parana; Ribeira de Iguape + Upper Parana + Iguassu; Southeastern Mata Atlantica; Southeastern Mata Atlantica + Iguassu; Southeastern Mata Atlantica + Ribeira de Iguape + Iguassu; Southeastern Mata Atlantica + Ribeira de Iguape + Upper Parana + Iguassu; and Upper Parana in CA 1 (Fig. 4), with 60 endemic species (Table 3); in Upper Parana and Upper Parana + Iguassu in CA 2 (Fig. 4), with 30 endemic species (Table 3); in Iguassu and Upper Parana + Iguassu in CA 3 (Fig. 4), with 31 endemic species (Table 3); in Upper Parana in CA 4 (Fig. 4), with four endemic species (Table 3); in Upper Parana in CA 5 (Fig. 4), with three endemic species (Table 3); and in Lower Parana; Lower Parana + Iguassu; and Lower Parana + Upper Parana + Iguassu in CA 6 (Fig. 4), with 46 endemic species (Table 3).

**Table 3:** Results of Endemicity Analysis for a 0.5° × 0.5° grid size

CAs	AEs	Ecoregion(s)	*N*	Endemic species (EI)	Size	*E*
1	3, 4, 8, 10, 17, 20, 22, 27, 28, 31, 32, 38, 39, 41, 42, 43, 47, 51, 53, 57, 60, 62, 66, 70, 72, 75, 76, 80, 82, 83, and 85	Ribeira de Iguape + Upper Parana/Ribeira de Iguape + Upper Parana + Iguassu/Southeastern Mata Atlantica/Southeastern Mata Atlantica + Iguassu/Southeastern Mata Atlantica + Ribeira de Iguape + Iguassu/Southeastern Mata Atlantica + Ribeira de Iguape + Upper Parana + Iguassu/Upper Parana	60	*Kronichthys lacerta* (0.75), *Astyanax laticeps* (0.86), *Deuterodon langei* (1.0), *Isbrueckerichthys duseni* (1.0), *Deuterodon iguape* (1.0), *Acentronichthys leptos* (1.0), *Hyphessobrycon bifasciatus* (0.63), *Characidium pterostictum* (0.32), *Harttia kronei* (1.0), *Hisonotus leucofrenatus* (1.0), *Scleromystax barbatus* (1.0), *Deuterodon janeiroensis* (0.64), *Characidium lanei* (0.75), *Hollandichthys multifasciatus* (1.0), *Hyphessobrycon griemi* (1.0), *Characidium schubarti* (1.0), *Rineloricaria kronei* (0.72), *Phalloceros pelos* (1.0), *Pseudotothyris obtusa* (0.60), *Mimagoniates lateralis* (0.83), *Parotocinclus maculicauda* (0.94), *Poecilia vivípara* (0.88), *Hypostomus interruptus* (1.0), *Microglanis cottoides* (1.0), *Chasmocranus lopezae* (0.79), *Neoplecostomus ribeirensis* (1.0), *Scleromystax macropterus* (1.0), *Bryconamericus microcephalus* (0.86), *Pimelodella transitoria* (0.79), *Schizolecis guntheri* (1.0), *Ancistrus multispinis* (1.0), *Pareiorhaphis azygolechis* (0.88), *Spintherobolus ankoseion* (0.83), *Cambeva iheringi* (0.42), *Rhamdioglanis transfasciatus* (0.83), *Glandulocauda caerulea* (0.58), *Gymnotus pantherinus* (1.0), *Astyanax* sp. 3 (1.0), *Astyanax totae* (0.75), *Crenicichla lacustris* (1.0), *Cambeva zonata* (1.0), *Hoplisoma longipinne*, *Rhamdiopsis moreirai* (0.46), *Rhamdioglanis frenatus* (0.79), *Crenicichla tingui* (0.83), *Characidium itarare* (1.0), *Cnesterodon carnegiei* (1.0), *Ituglanis proops* (1.0), *Kronichthys subterres* (0.75), *Phalloceros alessandrae* (0.83), *Atlantirivulus luelingi* (0.83), *Cambeva cubataonis* (0.71), *Cambeva pascuali* (0.83), *Phalloceros megapolos* (1.0), *Australoheros* sp. (0.75), *Isbrueckerichthys epakmos* (0.63), *Phalloptychus januarius* (0.83), *Astyanax* sp. 4 (1.0), *Hoplisoma steindachneri* (0.83), and *Leptopanchax aureoguttatus* (1.0)	17	2.2–23.23
2	5, 6, 7, 9, 11, 12, 13, 14, 15, 16, 18, 26, 29, 33, 35, 36, 40, 44, 45, 46, 49, 50, 55, 59, 61, 65, 74, 77, 81, and 88	Upper Parana/Upper Parana + Iguassu	30	*Hisonotus francirochai* (0.30), *Schizodon intermedius* (1.0), *Isbrueckerichthys calvus* (1.0), *Gymnotus omarorum* (1.0), *Cnesterodon hypselurus* (0.40), *Bryconamericus coeruleus* (1.0), *Rineloricaria latirostris* (0.32), *Cheirodon stenodon* (1.0), *Hisonotus pachysarkos* (1.0), *Neoplecostomus selenae* (0.54), *Apareiodon vladii* (0.56), *Hypostomus robertsoni* (1.0), *Neoplecostomus* sp. 2 (0.85), *Cambeva horacioi* (0.75), *Planaltina kaingang* (0.75), *Characidium heirmostigmata* (1.0), *Isbrueckerichthys saxicola* (0.56), *Hisonotus depressicauda* (0.70), *Brycon nattereri* (0.50), *Hoplisoma lacrimostigmata* (0.46), *Curculionichthys oliveirai* (0.41), *Bryconamericus misei* (0.58), *Cyphocharax* cf. *corumbae* (1.0), *Leporinus paranensis* (0.70), *Bunocephalus larai* (0.67), *Hypostomus* sp. 4 (0.50), *Eigenmannia* sp. (0.50), *Hypostomus* sp. 2 (1.0), *Neoplecostomus paranensis* (0.80), and *Rhyacoglanis paranensis* (0.50)	29	2.06–6.88
3	19, 23, 24, 25, 30, 34, 37, 48, 58, 67, 68, 71, 73, 79, 87, and 90	Iguassu/Upper Parana + Iguassu	31	*Crenicichla iguassuensis* (0.83), *Pimelodus ortmanni* (0.71), *Ancistrus mullerae* (0.70), *Crenicichla tesay* (0.62), *Psalidodon gymnogenys* (1.0), *Psalidodon gymnodontus* (1.0), *Glanidium ribeiroi* (0.67), *Cambeva stawiarski* (0.50), *Bryconamericus ikaa* (0.69), *Tatia jaracatia* (0.67), *Ancistrus agostinhoi* (0.81), *Hoplisoma carlae* (0.50), *Astyanax jordanensis* (1.0), *Jenynsia diphyes* (0.68), *Neoplecostomus* sp. 1 (0.83), *Cambeva igobi* (1.0), *Diapoma potamohadros* (1.0), *Bryconamericus pyahu* (0.58), *Cambeva crassicaudata* (1.0), *Cambeva papillifera* (1.0), *Steindachneridion melanodermatum* (0.54), *Crenicichla tuca* (0.75), *Cambeva cauim* (1.0), *Cambeva plumbea* (0.75), *Hisonotus yasi* (1.0), *Australoheros angiru* (1.0)	17	3.14–8.85
4	54, and 78	Upper Parana	4	*Poecilia hollandi* (0.83), *Amaralia oviraptor* (1.0), *Myloplus tiete* (1.0), and *Trachelyopterus* sp. (1.0)	3	3.0–3.33
5	56	Upper Parana	3	*Neoplecostomus* sp. 3 (0.57), *Hisonotus* sp. (0.70), and *Cambeva perobana* (0.80)	5	2.07
6	1, 2, 21, 52, 63, 64, 69, 84, 86, 89, and 91	Lower Parana/Lower Parana + Iguassu/Lower Parana + Upper Parana + Iguassu	46	*Diapoma guarani* (1.0), *Hoplisoma carlae* (0.30), *Catathyridium jenynsii* (0.79), *Loricariichthys rostratus* (0.93), *Crenicichla mandelburgeri* (0.88), *Roeboides descalvadensis* (0.88), *Hemiodus orthonops* (0.88), *Bryconamericus exodon* (0.88), *Saxatilia lepidota* (0.88), *Tetragonopterus argenteus* (0.79), *Hypophthalmus oremaculatus* (0.79), *Loricariichthys platymetopon* (1.0), *Pimelodella taenioptera* (0.88), *Ageneiosus ucayalensis* (0.88), *Auchenipterus osteomystax* (1.0), *Odontostilbe pequira* (1.0), *Serrasalmus marginatus* (0.90), *Cynopotamus kincaidi* (0.88), *Pterodoras granulosus* (1.0), *Trachelyopterus galeatus* (0.88), *Bujurquina vittata* (0.88), *Hypostomus cochliodon* (1.0), *Megaleporinus macrocephalus* (0.71), *Otocinclus vittatus* (0.71), *Platydoras armatulus* (0.88), *Trachydoras paraguayensis* (1.0), *Ageneiosus inermis* (0.88), *Gymnotus pantanal* (0.75), *Moenkhausia forestii* (0.88), *Pachyurus bonariensis* (1.0), *Pimelodus mysteriosus* (0.88), *Pimelodus ornatus* (0.75), *Plagioscion squamosissimus* (1.0), *Sorubim lima* (0.79), *Aphyocharax dentatus* (0.75), *Galeocharax humeralis* (0.88), *Ossancora eigenmanni* (0.88), *Paravandellia oxyptera* (1.0), *Psellogrammus kennedyi* (0.88), *Rhamphichthys hahni* (0.75), *Abramites hypselonotus* (1.0), *Farlowella hahni* (0.75), *Gymnotus paraguensis* (0.67), *Platanichthys platana* (0.64), *Pterygoplichthys ambrosettii* (0.75), and *Triportheus nematurus* (1.0)	9	4.5–30.49

CAs = Consensus Areas; AEs = Included Areas of Endemism in each CA (coding according to Table S3 and Figs. S63–S153); *N* = Number of endemic species in each CA; EI = highest individual value of the Endemicity Index; Size = number of grids of each CA; *E* = minimum and maximum final Endemicity scores of each CA.

### Prioritized areas of endemism

In total, 12 combinations of ecoregion(s) with the AEs recovered were computed for all grid sizes: Lower Parana; Iguassu; Upper Parana; Southeastern Mata Atlantica; Lower Parana + Iguassu; Lower Parana + Upper Parana + Iguassu; Ribeira de Iguape + Upper Parana; Ribeira de Iguape + Upper Parana + Iguassu; Southeastern Mata Atlantica + Iguassu; Southeastern Mata Atlantica + Ribeira de Iguape + Iguassu; Southeastern Mata Atlantica + Ribeira de Iguape + Upper Parana + Iguassu; and Upper Parana + Iguassu (Table 4). For all combinations of ecoregion(s) and AEs recovered, the minimum Endemicity (*E*) score was for AEs 34, 40, and 43 (0.3°; *E* = 2.0) located in the combination Ribeira de Iguape + Upper Parana or only Upper Parana (Tables S4, 4), and the maximum score was for AE 64 (0.5°; *E* = 30.49) located in the combination Lower Parana + Iguassu (Tables S4, 4). Regarding the Priority Index, the minimum score calculated was for AE 19 (0.3°; PI = −3.34) located in Upper Parana (Tables S4, 4), and the maximum score was for AE 16 (0.1°, PI = 5.19), located in the Southeastern Mata Atlantica (Tables S4, 4).

**Table 4:** Results of the ranking of Areas of Endemism (AEs) in conservation priorities considering only the first five positions between the Endemicity (E) and Priority Index (PI) scores for each combination of ecoregion(s), that is, grids of the AEs that are spatially overlapped in each ecoregion

Ecoregion(s)	*N* (0.1°)	*N* (0.3°)	*N* (0.5°)	*E* (máx/mín)	PI (máx/mín)	Ranking *E*	Ranking PI
Iguassu	0	6	4	2.64/7.54	1.02/2.03	(1st) AE 67 (0.5°), (2nd) AE 37 (0.5°), (3rd) AE 87 (0.5°), (4th) AE 79 (0.5°), and (5th) AE 5 (0.3°)	(1st) AE 79 (0.5°), (2nd) AE 5 (0.3°), (3rd) AE 20 (0.3°), (4th) AE 7 (0.3°), and (5th) AE 87 (0.5°)
Lower Parana	1	3	1	2.33/18.60	0,05/2.56	(1st) AE 8 (0.3°), (2nd) AE 24 (0.3°), (3rd) AE 38 (0.3), (4th) AE 86 (0.5°), and (5th) AE 14 (0.1°)	(1st) AE 8 (0.3°), (2nd) AE 24 (0.3°), (3rd) AE 38 (0.3), (4th) AE 14 (0.1°), and (5th) AE 86 (0.5°)
Lower Parana + Iguassu	7	3	7	2.18/30,49	−1.36/5.19	(1st) AE 64 (0.5°), (2nd) AE 89 (0.5°), (3rd) AE 2 (0.5°), (4th) AE 69 (0.5°), and (5th) AE 91 (0.5°)	(1st) AE 16 (0.1°), (2nd) AE 13 (0.1°), (3rd) AE 12 (0.1°), (4th) AE 15 (0.1°), and (5th) AE 8 (0.1°)
Lower Parana + Upper Parana + Iguassu	0	4	3	2.03/23.71	−1.37/2.74	(1st) AE 63 (0.5°), (2nd) AE 52 (0.5°), (3rd) AE 21 (0.5°), (4th) AE 39 (0.3°), and (5th) AE 12 (0.3°)	(1st) AE 12 (0.3°), (2nd) AE 63 (0.5°), (3rd) AE 39 (0.3°), (4th) AE 21 (0.5°), and (5th) AE 52 (0.5°)
Ribeira de Iguape + Upper Parana	0	5	6	2.0/5.24	−2.47/−0.31	(1st) AE 43 (0.5°), (2nd) AE 51 (0.5°), (3rd) AE 10 (0.5°), (4th) AE 39 (0.5°), and (5th) AE 17 (0.5°)	(1st) AE 43 (0.3°), (2nd) AE 34 (0.3°), (3rd) AE 16 (0.3°), (4th) AE 39 (0.5°), and (5th) AE 17 (0.5°)
Ribeira de Iguape + Upper Parana + Iguassu	0	4	6	3.01/9.28	0.30/1.54	(1st) AE 42 (0.5°), (2nd) AE 32 (0.5°), (3rd) AE 80 (0.5°), (4th) AE 4 (0.5°), and (5th) AE 72 (0.5°)	(1st) AE 82 (0.5°), (2nd) AE 3 (0.3°), (3rd) AE 9 (0.3°), (4th) AE 80 (0.5°), and (5th) AE 25 (0.3°)
Southeastern Mata Atlantica	9	2	1	2.13/5.69	−1.20/2.39	(1st) AE 36 (0.3°), (2nd) AE 4 (0.1°), (3rd) AE 10 (0.1°), (4th) AE 41 (0.5°), and (5th) AE 5 (0.1°)	(1st) AE 10 (0.1°), (2nd) AE 1 (0.1°), (3rd) AE 11 (0.1°), (4th) AE 3 (0.1°), and (5th) AE 6 (0.1°)
Southeastern Mata Atlantica + Iguassu	0	2	3	9.45/21.25	2.06/2.75	(1st) AE 31 (0.5°), (2nd) AE 57 (0.5°), (3rd) AE 85 (0.5°), (4th) AE 6 (0.3°), and (5th) AE 10 (0.3°)	(1st) AE 31 (0.5°), (2nd) AE 6 (0.3°), (3rd) AE 85 (0.5°), (4th) AE 10 (0.3°), and (5th) AE 57 (0.5°)
Southeastern Mata Atlantica + Ribeira de Iguape + Iguassu	0	10	3	2.16/23.23	2.27/3.27	(1st) AE 47 (0.5°), (2nd) AE 70 (0.5°), (3rd) AE 66 (0.5°), (4th) AE 13 (0.3°), and (5th) AE 17 (0.3°)	(1st) AE 70 (0.5°), (2nd) AE 2 (0.3°), (3rd) AE 66 (0.5°), (4th) AE 47 (0.5°), and (5th) AE 29 (0.3°)
Southeastern Mata Atlantica + Ribeira de Iguape + Upper Parana + Iguassu	0	0	10	7.41/18.94	1.81/3.64	(1st) AE 20 (0.5°), (2nd) AE 53 (0.5°), (3rd) AE 8 (0.5°), (4th) AE 62 (0.5°), and (5th) AE 38 (0.5°)	(1st) AE 53 (0.5°), (2nd) AE 3 (0.5°), (3rd) AE 8 (0.5°), (4th) AE 38 (0.5°), and (5th) AE 27 (0.5°)
Upper Parana	0	5	24	2.0/5.8	−3.34/1.40	(1st) AE 61 (0.5°), (2nd) AE 45 (0.5°), (3rd) AE 35 (0.5°), (4th) AE 50 (0.5°), and (5th) AE 59 (0.5°)	(1st) AE 40 (0.3°), (2nd) AE 23 (0.3°), (3rd) AE 26 (0.5°), (4th) AE 36 (0.5°), and (5th) AE 9 (0.5°)
Upper Parana + Iguassu	0	1	23	2.25/8.85	−2.65/3.43	(1st) AE 68 (0.5°), (2nd) AE 58 (0.5°), (3rd) AE 48 (0.5°), (4th) AE 71 (0.5°), and (5th) AE 12 (0.5°)	(1st) AE 34 (0.5°), (2nd) AE 58 (0.5°), (3rd) AE 30 (0.5°), (4th) AE 37 (0.3°), and (5th) AE 24 (0.5°)

*N* = number of AEs recovered for each set of ecoregion(s) in the three analyses performed (0.1° × 0.1°), (0.3° × 0.3°), and (0.5° × 0.5°); *E* = final Endemicity score of each AE; PI = Priority Index score of each AE.

**Fig. 5:**
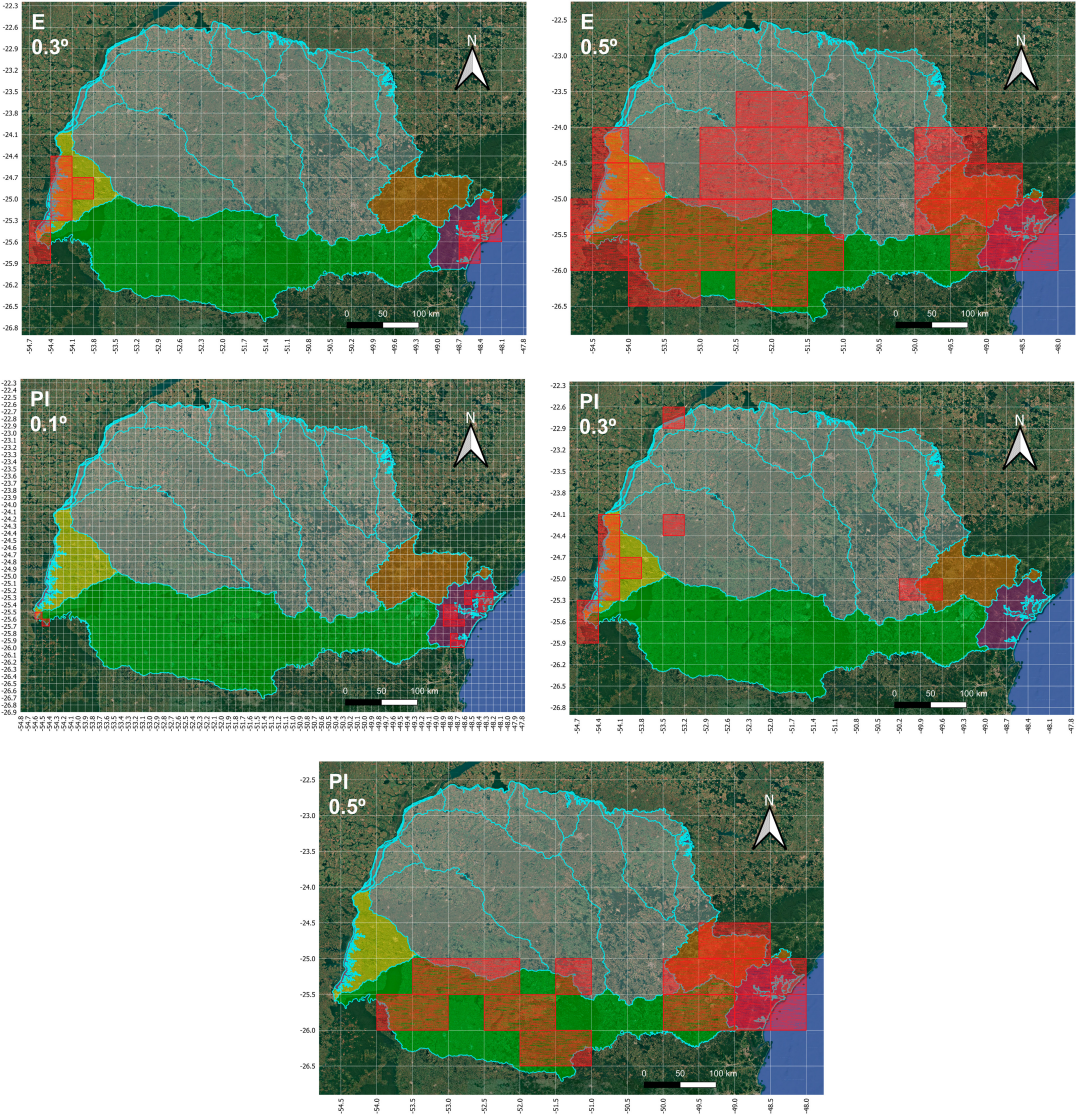
Maps summarizing the spatial optimization of Areas of Endemism (AEs) ranked based on their Endemicity (E) and Priority Index (PI) scores for all established combinations of ecoregion(s).

In general, for all combinations of ecoregion(s) there was a maximization of sets of similar species (endemic, sympatric and threatened) in smaller areas when the AEs were ranked (positioned) based on the Priority Index (PI) scores compared to the Endemicity (*E*) scores (Tables 4 and 5; Fig. 5). The numbers calculated in Table 5 and illustrated in Fig. 5 refer to the territorial extension represented as the number of grids at the smallest analysed size (0.1°). This measurement is necessary to represent and compare the spatial prioritization of AEs across all grid sizes analysed. These values account for the effect of grid size through the multiplication factor (*b* × *c*, see Material and methods) since 0.3° × 0.3° grids are nine times larger in area than 0.1° × 0.1° grids, while 0.5° × 0.5° grids are 25 times larger.

**Table 5:** Comparisons between Areas of Endemism (AEs) considering the first positions of the rankings based on the Endemicity (E) and Priority Index (PI) scores according to Table 4

Grid size	*E*			PI		
	(1st) AEs	Size	Equivalent grid	(1st) AEs	Size	Equivalent grid
0.1° × 0.1°	–	–	–	AE 16 and AE 10	20	20
0.3° × 0.3°	AE 8 and AE 36	10	90	AE 8, AE 12, AE 43, and AE 40	11	99
0.5° × 0.5°	AE 67, AE 64, AE 63, AE 43, AE 42, AE 31, AE 47, AE 20, AE 61, and AE 68	43	1075	AE 79, AE 82, AE 31, AE 70, AE 53, and AE 34	20	500
Total	–	–	1165	–	–	619

Size = total number of 0.1° × 0.1° grids (= equivalent territorial extension) considering the overlaps of the priority AEs according to Fig. 5. The calculation of the equivalent grid (*b* × *c*) is detailed in the “Materials and methods” section.

The PI sensitivity applies both to areas with smaller grids ranked in better positions within the combination (e.g. AE 64 recovered for 0.5° grid occupied the first place in the E score for the Lower Parana + Iguassu combination, while AE 16 recovered for 0.1° grid occupied the first place in the PI score for the same combination; Tables 4 and 5), and to areas with fewer grids within the same grid size (e.g. AE 42 with six 0.5° grids occupied the first place in the E score for the Ribeira de Iguape + Upper Parana + Iguassu combination, while AE 82 with four grids occupied the first place in the PI score for the same combination; Tables 4 and 5). The exception to maximization in smaller areas occurred only in the combination of Southeastern Mata Atlantica + Ribeira de Iguape + Upper Parana + Iguassu, since only AEs with 0.5° of grids were recovered for this combination (Table 4), that is, the effect of the area (Species‐Area attribute) was lost, with differences about endemicity (Endemicity attribute) and extinction (Extinction attribute) prevailing. Therefore, comparing the areas ranked first for each combination of ecoregion(s), a reduction of approximately 46.9% in the equivalent grid (territorial extension) is noted about the E (equivalent grid = 1165) and PI (equivalent grid = 619) scores (Table 5; Fig. 5).

## Discussion

### Biogeographic patterns in the areas of endemism

In this study, the size of the analysed grids directly influenced the number of AEs identified in the NDM/VNDM program. Specifically, increasing the number of grids led to a greater number of AEs. For instance, using a grid size of 0.1° resulted in 17 AEs categorized into two CAs, whereas a grid size of 0.5° yielded 91 AEs summarized in six CAs. Additionally, the EI and E scores were also proportional to the grid size and the number of endemic species present in the CAs (see Tables 1, 2, 3). Several studies have highlighted the impact of grid size on the delineation of AEs using endemism viewer programs (Szumik et al., 2002; Casagranda et al., 2009; Prado et al., 2015; Daru et al., 2020; Pacifico et al., 2020; Frota et al., 2021; Florentín et al., 2022; Della and Prado, 2024). Very small grids often result in discontinuous distributions, leading to few (or even no) small AEs being defined (Szumik et al., 2002). In contrast, larger grids tend to produce very extensive AEs with a higher number of endemic species (Szumik et al., 2002).

The CAs that overlap with the ecoregions of the Lower Parana exhibit the highest estimated E scores identified by the NDM/VNDM for the study area. Although these regions host several individual species recognized as endemic, most of these species are widely distributed across the entire Lower Parana and Paraguay ecoregions (Abell et al., 2008; see Ota et al., 2018). This distribution extends far beyond the limited area of the Lower Parana within Brazil, specifically the State of Paraná (Reis et al., 2020). Despite the significance of these areas for conserving aquatic biodiversity—due to their concentration of numerous sympatric species and some threatened with extinction—CAs defined in the Lower Parana ecoregion, including the final stretch of the Iguassu River below the Iguassu Falls, lack a cohesive ontological identity. The biotic elements in these areas often do not share a common evolutionary history. This poses a challenge that should be carefully considered when delineating AEs, especially in relation to federative units or political boundaries (Schultz and Cracraft, 2024). It would be more prudent to recognize potential barriers that complicate the identification of homologous units and the recovery of vicariance events (Hovenkamp, 1997; Szumik et al., 2019; Schultz and Cracraft, 2024). Neighbouring endemic areas may not necessarily be characterized by sister taxa, which indicates a lack of a shared historical process that formed these areas (Szumik et al., 2019). In this context, it can be said that the Sete Quedas waterfalls effectively separate the fish lineages in the Lower Parana ecoregion. Additionally, among various definitions of AEs in the literature, the CAs of the Lower Parana may include species that have been established through long‐distance colonization (see Crother and Murray, 2011; Schultz and Cracraft, 2024). These areas may not contain endemic taxa, yet they could still be classified as AEs (Linder, 2001; Schultz and Cracraft, 2024).

Except for the Lower Parana ecoregion, the results indicated that species considered endemic to each ecoregion (Reis et al., 2020) contributed to an increase in the *E* scores of their respective CAs across all grid sizes and sets of overlapping ecoregions in the study area. In the Atlantic slope river basins, specifically the Southeastern Mata Atlantica and Ribeira de Iguape ecoregions, 23.4% of fish genera and 95.0% of fish species are endemic (Ribeiro, 2006). These high levels of endemism are likely linked to the ancient biogeographic history of these lineages, which originated during the Mesozoic (Cretaceous) period and experienced cladogenetic events during the Paleogene and Neogene (Ribeiro, 2006; Cassemiro et al., 2023). The uplift of the Serra do Mar, occurring around 23–16 million years ago (Ma), isolated the Atlantic slope river basins from the Interior slope. This uplift created stable relict habitats and provided opportunities for vicariance at higher elevations (Frota et al., 2021; Cassemiro et al., 2023). These areas have recently been described as historically stable refugia for taxa in the Atlantic Forest, supporting high levels of genetic diversity and endemism across several groups (Ribeiro, 2006; Frota et al., 2019, 2021; Cassemiro et al., 2023).

In the river basins that flow into the Interior Slope, the Iguassu and Upper Parana ecoregions exhibit significant fish endemism rates in the State of Paraná, with 67.4% and 10.7%, respectively (Reis et al., 2020). The high level of fish endemism in the Iguassu River basin has been linked to the formation of the Iguassu Falls (Baumgartner et al., 2012; Mezzaroba et al., 2021) and the reactivation of drainage “breaks,” which likely delineated its distinct sub‐basins (Garavello and Sampaio, 2010). The E scores of the AEs that overlap with the Upper Parana ecoregion reflect the degree of fish species endemism in this region, particularly in the Ivaí, Piquiri, Paranapanema, and Floodplain subecoregions (Reis et al., 2020). The fish endemism in the Ivaí subecoregion has been attributed to the presence of waterfalls in its upper reaches, near the Serra da Esperança (Frota et al., 2016a), as well as a strong correlation between dispersal and niche processes in its headwaters (Frota et al., 2022). Several exclusive fish species are shared between the Ivaí and Piquiri subecoregions (Reis et al., 2020), supporting the observation that the AEs identified in these areas (with grid sizes of 0.3° and 0.5°) are situated within an exclusive bioregion (Dagosta et al., 2024). Similarly, the AEs overlapping with the Paranapanema subecoregion (Reis et al., 2020) are also found in exclusive bioregions, specifically Ponta Grossa Arch I and II (Dagosta et al., 2024). These AEs are part of a region in the Upper Parana that has deep historical connections with neighbouring basins (Ribeiro, 2006; Morais‐Silva et al., 2018; Frota et al., 2019, 2020, 2021; Dagosta et al., 2024). Notably, several species exclusive to these AEs in the Upper Parana are also found in adjacent ecoregions. These species and/or lineages are primarily located in the Upper Parana but have broader distributions in other ecoregions, such as Iguassu, the Southeastern Mata Atlantica, and Ribeira de Iguape (Ribeiro, 2006; Dagosta et al., 2024).

The numerous examples of endemic fish species shared across distinct CAs and ecoregions are not merely coincidental; they indicate a shared biogeographic history of these regions. For instance, the headwaters of the Upper Parana, Iguassu, Ribeira de Iguape, and Southeastern Mata Atlantica ecoregions are linked to the geomorphological dynamics of the Ponta Grossa Arch. This geological formation features a complex system of fault lineaments and rifted blocks that create highly dynamic tectonic activity (Ribeiro, 2006; Franco‐Magalhães et al., 2010). Vertical movements between faulted blocks and the erosive evolution of rivers along these rifts facilitate the mixing of fish populations between adjacent drainages (Ribeiro, 2006; Morais‐Silva et al., 2018; Frota et al., 2019, 2020, 2021; Dagosta et al., 2024; Reis et al., 2025). This phenomenon helps define the AEs and specific bioregions, as previously mentioned (see Dagosta et al., 2024). These processes suggest a strong connection between vicariance and faunal exchange, which closely aligns with the biogeographic patterns identified in the AEs (Ribeiro, 2006; Frota et al., 2016a, b, 2019, 2020; Morais‐Silva et al., 2018; Reis et al., 2025). This connection highlights the significance of these areas for fish diversification and conservation in Southern Brazil (Affonso et al., 2015; Frota et al., 2016a, b, 2019, 2020, 2021; Cavalli et al., 2018; Alves et al., 2019).

### Conservation priorities

In the study area, AEs for freshwater fish exhibited spatial overlap across all five ecoregions. Larger grid sizes (0.3° and 0.5°) were more effective in identifying areas that spanned across ecoregion boundaries, while smaller grids (0.1°) delineated smaller areas that were confined to individual ecoregions. These findings indicate that, in conservation planning, it is important to employ different grid sizes to effectively identify priority areas for protecting both local endemic species and those with wider distributions (Aagesen et al., 2009; Casagranda et al., 2009; DaSilva et al., 2015; Prado et al., 2015; Ocampo‐Salinas et al., 2019; Frota et al., 2021; Della and Prado, 2024).

The combinations of ecoregion(s) represent unique compositions of endemic, sympatric, and threatened species. Except for the Southeastern Mata Atlantica + Ribeira de Iguape + Upper Parana + Iguassu combination, which was defined only by 0.5° grids, all other combinations were identified in at least two analyses using different grid sizes (see Table 4). While utilizing larger grids can increase the likelihood of including areas with greater ecosystem diversity, which may obscure observed biogeographic patterns (Sigrist and Carvalho, 2008), the use of different grid sizes is viewed as a measure of support for AEs (Casagranda et al., 2009; Hoffmeister and Ferrari, 2016; Florentín et al., 2022; Narváez‐Gómez et al., 2022; Della and Prado, 2024). When an area is identified across various grid sizes, it is considered more “robust” than an area found only with a single grid size (Florentín et al., 2022; Della and Prado, 2024).

The spatial and taxonomic distribution of fish lineages in the study area is not random; it reflects various ecological and evolutionary processes. This pattern helps identify regions that host endemic species (Abell et al., 2008; Albert et al., 2020). Despite some criticism, ecoregions have been utilized in studies focused on the conservation of other freshwater organisms in Southern Brazil (Gonçalves et al., 2018; Tumini et al., 2019; Frota et al., 2021). Accordingly, areas with overlapping ecoregions may encompass distinct habitats, which can serve as incubators for biological specialization and promote high rates of diversification in lineages and phenotypes (Abell et al., 2008; Frota et al., 2021).

Many of the AEs recovered in different combinations of ecoregion(s) include headwater locations of river basins that exhibit unique evolutionary patterns. These headwaters, characterized by their rugged terrain, may have promoted increased genetic differentiation among local populations (Thomaz et al., 2016). Over time, this differentiation could lead to species restriction or endemism (Carvajal‐Quintero et al., 2019; Frota et al., 2021). While freshwater fish diversification typically occurs under these conditions and at small geographic scales, particularly in response to landscape evolution (Albert et al., 2020; Cassemiro et al., 2023), it is important to note that ecoregions incorporate various ecological and evolutionary processes. Therefore, they are expected to exhibit unique patterns of species distribution and composition (Abell et al., 2008). This underscores the necessity for conservation efforts in AEs that overlap with ecoregions. Effective management strategies for the long‐term conservation of biological diversity should be developed, taking into account local community patterns alongside the processes that drive evolutionary diversification (Frota et al., 2021).

The results indicated that the proposed Priority Index was more effective in identifying smaller territorial areas. These areas could potentially support similar species richness, including endemic and threatened species, as well as the evolutionary diversification processes (i.e. biogeographic patterns) of fish lineages that are connected to the historical relationships between the AEs and the associated ecoregions. When we applied the index and ranked the AEs, we observed an almost 50% reduction in spatial area compared to the ranking that considered only endemic species (i.e. the final score E). There is a widely acknowledged need for conservation actions to be cost‐effective, which has led to increased interest and effort in evaluating their effectiveness (Martin et al., 2012; Kukkala and Moilanen, 2013; Smith et al., 2022; Giakoumi et al., 2025). The Priority Index could be utilized alongside endemicity analysis aimed at biodiversity conservation, as decision‐makers often face constraints related to human resources, funding, or data quality. Consequently, they may seek to acquire land with high biodiversity values while minimizing transaction and management costs (Rodewald et al., 2019; Smith et al., 2022; Giakoumi et al., 2025).

It is important to emphasize that the objectives of the spatial conservation prioritization approach should extend beyond merely designating protected areas (Kukkala and Moilanen, 2013). To evaluate the sustainability of defined spatial areas, it is highly recommended to incorporate additional data when determining priority areas for conservation. This includes considering the cost of conservation, opportunities for action, and the evolutionary history of biodiversity, along with integrating environmental, geographic, and social data. The grids established from the combination of Endemicity Analysis and Priority Index do not necessarily represent priority conservation sites; however, they are crucial for supporting systematic conservation planning and identifying priority areas.

Humanity faces a critical moment in enhancing the training of local authorities on regional conservation planning. It is essential to motivate these authorities to identify new conservation areas through programs that assess and plan for conservation on a regional scale (Knight et al., 2007). By doing so, society can benefit from effective conservation policies, practices, and research, which incorporate systematic conservation planning and spatial prioritization approaches (Kukkala and Moilanen, 2013; Smith et al., 2022; Giakoumi et al., 2025). The next step is to engage with policymakers to determine how to evaluate the human dimensions of the region and to facilitate the exchange of science‐based information, policy decisions, and management strategies (Darwall et al., 2018). Bridging the gap between conservation science and policy decisions represents a significant and urgent challenge (Azevedo‐Santos et al., 2017; Frota and Frota, 2018).

If managed properly, the AEs positioned by the Priority Index and ranked for all combinations of ecoregion(s) can be critical for the long‐term protection of several freshwater fish species in Southern Brazil, including numerous endemic and threatened species. We hope that the methodology proposed here regarding distribution patterns, endemism, sympatry, and threatened species, while considering optimizations of the spatial size necessary for prioritization, can be used to identify new priority areas for biodiversity conservation (both aquatic and terrestrial). This approach has proven effective in reconciling attributes easily extracted from the NDM/VNDM program when prioritizing spatial conservation. Therefore, we encourage future studies that adopt similar approaches or apply the Priority Index attributes in Endemicity Analysis to support monitoring programs, management, and policies favouring biodiversity conservation.

## Conflict of interest

The authors declare that they have no conflict of interest.

## Supporting information


**Figs. S1–S153.** Figures showing all Areas of Endemism (AEs) recovered for all grid sizes by the NDM/VNDM software.

**Table S1.** Spreadsheet containing details regarding the calculations of the Priority Index for Areas of Endemism (AEs) in 0.1° × 0.1° grids.

**Table S2.** Spreadsheet containing details regarding the calculations of the Priority Index for Areas of Endemism (AEs) in 0.3° × 0.3° grids.

**Table S3.** Spreadsheet containing details regarding the calculations of the Priority Index for Areas of Endemism (AEs) in 0.5° × 0.5° grids.

**Table S4.** Spreadsheet containing the Endemicity (E) and Priority Index (PI) scores to rank the Areas of Endemism (AEs) recovered endemism for all grid sizes.

**Data S1.** Final database of georeferenced records for native fish species from the State of Paraná used in the Endemicity Analysis performed.

## Data Availability

Distribution data are available in Supporting Information.

## References

[cla70002-bib-0001] Aagesen, L. , Szumik, C. , Zuloaga, F.O. and Morrone, O. , 2009. Quantitative biogeography in the South America highlands–recognizing the Altoandina, Puna and Prepuna through the study of Poaceae. Cladistics 25, 295–310. 34879615 10.1111/j.1096-0031.2009.00248.x

[cla70002-bib-0002] Aagesen, L. , Szumik, C. and Goloboff, P. , 2013. Consensus in the search for areas of endemism. J. Biogeogr. 40, 2011–2016.

[cla70002-bib-0003] Abell, R. , Thieme, M.L. , Revenga, C. , Bryer, M. , Kottelat, M. , Bogutskaya, N. , Coad, B. , Mandrak, N. , Balderas, S.C. , Bussing, W. , Stiassny, M.L.J. , Skelton, P. , Allen, G.R. , Unmack, P. , Naseka, A. , Ng, R. , Sindorf, N. , Robertson, J. , Armijo, E. , Higgins, J.V. , Heibel, T.J. , Wikramanayake, E. , Olson, D. , López, H.L. , Reis, R.E. , Lundberg, J.G. , Sabaj Pérez, M.H. and Petry, P. , 2008. Freshwater ecoregions of the world: A new map of biogeographic units for freshwater biodiversity conservation. Bioscience 58, 403–414.

[cla70002-bib-0004] Affonso, I.P. , Azevedo, R.F. , dos Santos, N.L.C. , Dias, R.M. , Agostinho, A.A. and Gomes, L.C. , 2015. Pulling the plug: Strategies to preclude expansion of dams in Brazilian rivers with high‐priority for conservation. Nat. Conserv. 13, 199–203.

[cla70002-bib-0005] Albert, J.S. , Tagliacollo, V.A. and Dagosta, F. , 2020. Diversification of Neotropical freshwater fishes. Annu. Rev. Ecol. Evol. Syst. 51, 27–53.

[cla70002-bib-0006] Alves, C.B.M. , Pompeu, P.S. , Mazzoni, R. and Brito, M.F.G. , 2021. Avanços em métodos de coleta de peixes e caracterização de habitat de riachos tropicais. Oecol. Aust. 25, 246–265.

[cla70002-bib-0007] Alves, G.H.Z. , Santos, R.S. , Figueiredo, B.R.S. , Manetta, G.I. , Message, H.J. , Pazianoto, L.H.R. , Guimarães, G.B. , Benedito, E. and Couto, E.V. , 2019. Misguided policy may jeopardize a diverse South Brazilian environmental protection area. Biota Neotrop. 19, e20180574. 10.1590/16760611-BN-2018-0574.

[cla70002-bib-0008] Azevedo‐Santos, V.M. , Fearnside, P.M. , Oliveira, C.S. , Padial, A.A. , Pelicice, F.M. , Lima, D.P., Jr. , Simberloff, D. , Lovejoy, T.E. , Magalhães, A.L.B. , Orsi, M.L. , Agostinho, A.A. , Esteves, F.A. , Pompeu, P.S. , Laurance, W.F. , Petrere, M., Jr. , Mormul, R.P. and Vitule, J.R.S. , 2017. Removing the abyss between conservation science and policy decisions in Brazil. Biodivers. Conserv. 26, 1745–1752.

[cla70002-bib-0009] Azevedo‐Santos, V.M. , Frederico, R.G. , Fagundes, C.K. , Pompeu, P.S. , Pelicice, F.M. , Padial, A.A. , Nogueira, M.G. , Fearnside, P.M. , Lima, L.B. , Daga, V.S. , Oliveira, F.J.M. , Vitule, J.R.S. , Callisto, M. , Agostinho, A.A. , Esteves, F.A. , Lima‐Junior, D.P. , Magalhães, A.L.B. , Sabino, J. , Mormul, R.P. , Grasel, D. , Zuanon, J. , Vilella, F.S. and Henry, R. , 2019. Protected areas: A focus on Brazilian freshwater biodiversity. Divers. Distrib. 25, 442–448.

[cla70002-bib-0010] Bailly, D. , Batista‐Silva, V.F. , Cassemiro, F.A. , Lemes, P. , Graça, W.J. , de Oliveira, A.G. , do Couto, E.V. , Ferreira, J.H.D. , Ré, R. , Rangel, T.F. and Agostinho, A.A. , 2021. The conservation of migratory fishes in the second largest river basin of South America depends on the creation of new protected areas. Aquat. Conserv. 31, 2515–2532.

[cla70002-bib-0011] Baumgartner, G. , Pavanelli, C.S. , Baumgartner, D. , Bifi, A.G. , Debona, T. and Frana, V.A. , 2012. Peixes do Baixo Rio Iguaçu. Eduem, Maringá.

[cla70002-bib-0012] Carvajal‐Quintero, J. , Villalobos, F. , Oberdorff, T. , Grenouillet, G. , Brosse, S. , Hugueny, B. , Jézéquel, C. and Tedesco, P.A. , 2019. Drainage network position and historical connectivity explain global patterns in freshwater fishes' range size. Proc. Natl. Acad. Sci. U. S. A. 116, 13434–13439. 31209040 10.1073/pnas.1902484116 PMC6613146

[cla70002-bib-0013] Casagranda, M.D. , Roig‐Juñent, S. and Szumik, C. , 2009. Endemismo a diferentes escalas espaciales: Un ejemplo con Carabidae (Coleoptera: Insecta) de América del Sur austral. Rev. Chil. Hist. Nat. 82, 17–42.

[cla70002-bib-0014] Cassemiro, F.A.S. , Albert, J.S. , Antonelli, A. , Menegotto, A. , Wüest, R.O. , Cerezer, F. , Coelho, M.T.P. , Reis, R.E. , Tan, M. , Tagliacollo, V. , Bailly, D. , da Silva, V.F.B. , Frota, A. , da Graça, W.J. , Ré, R. , Ramos, T. , Oliveira, A.G. , Dias, M.S. , Colwell, R.K. , Rangel, T.F. and Graham, C.H. , 2023. Landscape dynamics and diversification of the megadiverse South American freshwater fish fauna. Proc. Natl. Acad. Sci. U. S. A. 120, e2211974120. 10.1073/pnas.2211974120. 36595684 PMC9926176

[cla70002-bib-0015] Cavalli, D. , Frota, A. , Lira, A.D. , Gubiani, É.A. , Margarido, V.P. and Graça, W.J. , 2018. Update on the ichthyofauna of the Piquiri River basin, Paraná, Brazil: a conservation priority area. Biota Neotrop. 18, e20170350. 10.1590/1676-0611-BN-2017-0350.

[cla70002-bib-0016] Chakona, A. , Gouws, G. , Kadye, W.T. , Jordaan, M.S. and Swartz, E.R. , 2019. Reconstruction of the historical distribution ranges of imperilled stream fishes from a global endemic hotspot based on molecular data: Implications for conservation of threatened taxa. Aquat. Conserv. 30, 144–158.

[cla70002-bib-0017] Cracraft, J. , 1985. Historical biogeography and patterns of differentiation within the south American avifauna: Areas of endemism. Ornithol. Monogr. 36, 49–84.

[cla70002-bib-0018] Crother, B.I. and Murray, C.M. , 2011. Ontology of areas of endemism. J. Biogeogr. 38, 1009–1015.

[cla70002-bib-0019] Dagosta, F.C.P. , de Pinna, M. , Peres, C.A. and Tagliacollo, V.A. , 2021. Existing protected areas provide a poor safety‐net for threatened Amazonian fish species. Aquat. Conserv. 31, 1167–1189.

[cla70002-bib-0020] Dagosta, F.C. , Monção, M.S. , Nagamatsu, B.A. , Pavanelli, C.S. , Carvalho, F.R. , Dagosta, F.C.P. , Lima, F.C.T. , Langeani, F. , Dutra, G.M. , Ota, R.R. , Seren, T.J. , Tagliacollo, V. , Menezes, N.A. , Britski, H.A. , de Pinna, M. and de Pinna, M. , 2024. Fishes of the upper rio Paraná basin: diversity, biogeography and conservation. Neotrop. Ichthyol. 22, e230066. 10.1590/1982-0224-2023-0066.

[cla70002-bib-0021] Daru, B.H. , Farooq, H. , Antonelli, A. and Faurby, S. , 2020. Endemism patterns are scale dependent. Nat. Commun. 11, 2115. 10.1038/s41467-020-15921-6. 32355257 PMC7192928

[cla70002-bib-0022] Darwall, W.R.T. and Vié, J.‐C. , 2005. Identifying important sites for conservation of freshwater biodiversity: extending the species‐based approach. Fish. Manag. Ecol. 12, 287–293.

[cla70002-bib-0023] Darwall, W. , Bremerich, V. , De Wever, A. , Dell, A.I. , Freyhof, J. , Gessner, M.O. , Grossart, H.‐.P. , Harrison, I. , Irvine, K. , Jähnig, S.C. , Jeschke, J.M. , Lee, J.J. , Lu, C. , Lewandowska, A.M. , Monaghan, M.T. , Nejstgaard, J.C. , Patricio, H. , Schmidt‐Kloiber, A. , Stuart, S.N. , Thieme, M. , Tockner, K. , Turak, E. and Weyl, O. , 2018. The *Alliance for Freshwater Life*: a global call to unite efforts for freshwater biodiversity science and conservation. Aquat. Conserv. 28, 1015–1022.

[cla70002-bib-0024] DaSilva, M.B. , Pinto‐da‐Rocha, R. and DeSouza, A.M. , 2015. A protocol for the delimitation of areas of endemism and the historical regionalization of the Brazilian Rain Forest using harvestmen distribution data. Cladistics 31, 692–705. 34753274 10.1111/cla.12121

[cla70002-bib-0025] Della, A.P. and Prado, J. , 2024. Areas of endemism of Pteridaceae (Polypodiopsida) in Brazil: a first approach. Cladistics 40, 157–180. 38124237 10.1111/cla.12568

[cla70002-bib-0026] Domínguez, M.C. , Roig‐Juñent, S. , Tassin, J.J. , Ocampo, F.C. and Flores, G.E. , 2006. Areas of endemism of the Patagonian steppe: an approach based on insect distributional patterns using endemicity analysis. J. Biogeogr. 33, 1527–1537.

[cla70002-bib-0027] Ebach, M.C. , Morrone, J.J. , Parenti, L.R. and Viloria, A.L. , 2008. International code of area nomenclature. J. Biogeogr. 35, 1153–1157.

[cla70002-bib-0028] Escalante, T. , 2015. Parsimony analysis of endemicity and analysis of endemicity: a fair comparison. Syst. Biodivers. 13, 413–418.

[cla70002-bib-0029] Florentín, J.E. , Salas, R.M. , Jarvie, S. , Svenning, J.C. and Gómez, J.M.D. , 2022. Areas of endemism and conservation status of *Galianthe* species (Spermacoceae, Rubiaceae) in the Neotropics. Syst. Biodivers. 20, 2025946. 10.1080/14772000.2022.2025946.

[cla70002-bib-0030] Franco‐Magalhães, A.O.B. , Hackspacher, P.C. , Glasmacher, U.A. and Saad, A.R. , 2010. Rift to post‐rift evolution of a “passive” continental margin: The Ponta Grossa Arch, SE Brazil. Int. J. Earth Sci. 99, 1599–1613.

[cla70002-bib-0031] Fricke, R. , Eschmeyer, W.N. and Van der Laan, R. , 2025. Eschmeyer's catalog of fishes: genera, species, references. Available at: http://researcharchive.calacademy.org/research/ichthyology/catalog/fishcatmain.asp (accessed 9 March 2025).

[cla70002-bib-0032] Frota, A. and Frota, M. , 2018. Brazilian conservation under the light of historical materialism. Ecol. Econ. 145, 472–475.

[cla70002-bib-0033] Frota, A. , Deprá, G.C. , Petenucci, L.M. and Graça, W.J. , 2016a. Inventory of the fish fauna from Ivaí River basin, Paraná State, Brazil. Biota Neotrop. 16, e20150151. 10.1590/1676-0611-BN-2015-0151.

[cla70002-bib-0034] Frota, A. , Gonçalves, E.V.R. , Deprá, G.C. and Graça, W.J. , 2016b. Inventory of the ichthyofauna from the Jordão and Areia river basins (Iguaçu drainage, Brazil) reveals greater sharing of species than thought. Check List 12, 1995. 10.15560/12.6.1995.

[cla70002-bib-0035] Frota, A. , Message, H.J. , Oliveira, R.C. , Benedito, E. and Graça, W.J. , 2019. Ichthyofauna of headwater streams from the rio Ribeira de Iguape basin, at the boundaries of the Ponta Grossa Arch, Paraná, Brazil. Biota Neotrop. 19, e20180666. 10.1590/1676-0611-BN-2018-0666.

[cla70002-bib-0036] Frota, A. , Ota, R.R. , Deprá, G.C. , Ganassin, M.J.M. and Graça, W.J. , 2020. A new inventory for fishes of headwater streams from the rio das Cinzas and rio Itararé basins, rio Paranapanema system, Paraná, Brazil. Biota Neotrop. 20, e20190833. 10.1590/1676-0611-BN-2019-0833.

[cla70002-bib-0037] Frota, A. , Pacifico, R. and Graça, W.J. , 2021. Selecting areas with rare and restricted fish species in mountain streams of Southern Brazil. Aquat. Conserv. 31, 1269–1284.

[cla70002-bib-0038] Frota, A. , Ganassin, M.J.M. , Pacifico, R. , Gomes, L.C. and Graça, W.J. , 2022. Spatial distribution patterns and predictors of fish beta‐diversity in a large dam‐free tributary from a Neotropical floodplain. Ecohydrology 15, e2376. 10.1002/eco.2376.

[cla70002-bib-0039] Garavello, J.C. and Sampaio, F.A.A. , 2010. Five new species of genus *Astyanax* Baird & Girard, 1854 from Rio Iguaçu, Brazil (Ostariophysi, Characiformes, Characidae). Braz. J. Biol. 70, 847–865. 21085790 10.1590/s1519-69842010000400016

[cla70002-bib-0040] Giakoumi, S. , Richardson, A.J. , Doxa, A. , Moro, S. , Andrello, M. , Hanson, J.O. , Hermoso, V. , Mazor, T. , McGowan, J. , Kujala, H. , Law, E. , Álvarez‐Romero, J.G. , Magris, R.A. , Gissi, E. , Arafeh‐Dalmau, N. , Metaxas, A. , Virtanen, E.A. , Ban, N.C. , Runya, R.M. , Dunn, D.C. , Fraschetti, S. , Galparsoro, I. , Smith, R.J. , Bastardie, F. , Stelzenmüller, V. , Possingham, H.P. and Katsanevakis, S. , 2025. Advances in systematic conservation planning to meet global biodiversity goals. Trends Ecol. Evol. 40, 395–410. 39880725 10.1016/j.tree.2024.12.002

[cla70002-bib-0041] Goloboff, P. , 2024. NDM and VNDM: programs for the identification of areas of endemism, vers. 3. Program and documentation. Available at: http://www.lillo.org.ar/phylogeny (accessed 9 March 2025).

[cla70002-bib-0042] Gomes‐da‐Silva, J. , Amorim, A.M. and Forzza, R.C. , 2017. Distribution of the xeric clade species of Pitcairnioideae (Bromeliaceae) in South America: a perspective based on areas of endemism. J. Biogeogr. 44, 1994–2006.

[cla70002-bib-0043] Gonçalves, A.S. , Costa, G.C. , Bond‐Buckup, G. , Bartholomei‐Santos, M.L. and Santos, S. , 2018. Priority areas for conservation within four freshwater ecoregions in South America: A scale perspective based on freshwater crabs (Anomura, Aeglidae). Aquat. Conserv. 28, 1077–1088.

[cla70002-bib-0044] Guedes, T.B. , Sawaya, R.J. and Nogueira, C.C. , 2014. Biogeography, vicariance and conservation of snakes of the neglected and endangered caatinga region, North‐Eastern Brazil. J. Biogeogr. 41, 919–931.

[cla70002-bib-0045] Heilpern, S. , 2015. Biodiversity: Include freshwater species. Nature 518, 167. 10.1038/518167d 25673401

[cla70002-bib-0046] Hijmans, R.J. , Guarino, L. , Cruz, M. and Rojas, E. , 2001. Computer tools for spatial analysis of plant genetic resources data: 1. DIVA‐GIS. Plant Genet. Resour. Newslett. 2001, 15–19.

[cla70002-bib-0047] Hoffmeister, C.H. and Ferrari, A. , 2016. Areas of endemism of arthropods in the Atlantic Forest (Brazil): an approach based on a metaconsensus criterion using endemicity analysis. Biol. J. Linn. Soc. 119, 126–144.

[cla70002-bib-0048] Hovenkamp, P. , 1997. Vicariance events, not areas, should be used in biogeographical analysis. Cladistics 13, 67–79. 34920640 10.1111/j.1096-0031.1997.tb00241.x

[cla70002-bib-0049] IBGE – Instituto Brasileiro de Geografia e Estatística , 2022. Censo demográfico. Available at: https://cidades.ibge.gov.br/brasil/pr (accessed 9 March 2025).

[cla70002-bib-0050] ICMBio – Instituto Chico Mendes de Conservação da Biodiversidade , 2025. Sistema de Avaliação do Risco de Extinção da Biodiversidade – SALVE. Available at: https://salve.icmbio.gov.br (accessed 9 March 2025).

[cla70002-bib-0051] IPARDES – Instituto Paranaense de Desenvolvimento Econômico e Social , 2017. Indicadores de desenvolvimento sustentável por bacias hidrográficas do Estado do Paraná. IPARDES, Curitiba.

[cla70002-bib-0052] Jarduli, L.R. , Garcia, D.A.Z. , Vidotto‐Magnoni, A.P. , Casimiro, A.C.R. , Vianna, N.C. , de Almeida, F.S. , Jerep, F.C. and Orsi, M.L. , 2020. Fish fauna from the Paranapanema River basin, Brazil. Biota Neotrop. 20, e20180707. 10.1590/1676-0611-BN-2018-0707.

[cla70002-bib-0053] Jézéquel, C. , Tedesco, P.A. , Darwall, W. , Dias, M.S. , Frederico, R.G. , Hidalgo, M. , Hugueny, B. , Maldonado‐Ocampo, J. , Martens, K. , Ortega, H. , Torrente‐Vilara, G. , Zuanon, J. and Oberdorff, T. , 2020. Freshwater fish diversity hotspots for conservation priorities in the Amazon basin. Conserv. Biol. 34, 956–965. 31990088 10.1111/cobi.13466

[cla70002-bib-0054] Júlio Júnior, H.F. , Dei Tós, C. , Agostinho, A.A. and Pavanelli, C.S. , 2009. A massive invasion of fish species after eliminating a natural barrier in the upper rio Paraná basin. Neotrop. Ichthyol. 7, 709–718.

[cla70002-bib-0055] Knight, A.T. , Smith, R.J. , Cowling, R.M. , Desmet, P.G. , Faith, D.P. , Ferrier, S. , Gelderblom, C.M. , Grantham, H. , Lombard, A.T. , Maze, K. , Nel, J.L. , Parrish, J.D. , Pence, G.Q.K. , Possingham, H.P. , Reyers, B. , Rouget, M. , Roux, D. and Wilson, K.A. , 2007. Improving the key biodiversity areas approach for effective conservation planning. Bioscience 57, 256–261.

[cla70002-bib-0056] Kukkala, A.S. and Moilanen, A. , 2013. Core concepts of spatial prioritisation in systematic conservation planning. Biol. Rev. Camb. Philos. Soc. 88, 443–464. 23279291 10.1111/brv.12008 PMC3654170

[cla70002-bib-0057] Linder, H.P. , 2001. On areas of endemism, with an example from the African Restionaceae. Syst. Biol. 50, 892–912. 12116639 10.1080/106351501753462867

[cla70002-bib-0058] Maack, R. , 2017. Geografia física do Estado do Paraná. Editora UEPG, Ponta Grossa.

[cla70002-bib-0059] Margules, C.R. and Pressey, R.L. , 2000. Systematic conservation planning. Nature 405, 243–253. 10821285 10.1038/35012251

[cla70002-bib-0060] Martin, T.G. , Burgman, M.A. , Fidler, F. , Kuhnert, P.M. , Low‐Choy, S. , Mcbride, M. and Mengersen, K. , 2012. Eliciting expert knowledge in conservation science. Conserv. Biol. 26, 29–38. 22280323 10.1111/j.1523-1739.2011.01806.x

[cla70002-bib-0061] Martínez‐Hernández, F. , Mendoza‐Fernández, A.J. , Pérez‐García, F.J. , Martínez‐Nieto, M.I. , Garrido‐Becerra, J.A. , Salmerón‐Sánchez, E. , Merlo, M.E. , Gil, C. and Mota, J.F. , 2015. Areas of endemism as a conservation criterion for Iberian gypsophilous flora: a multiscale test using the NDM/VNDM program. Plant Biosyst. 149, 483–493.

[cla70002-bib-0062] Mezzaroba, L. , Debona, T. , Frota, A. , Graça, W.J. and Gubiani, É.A. , 2021. From the headwaters to the Iguassu Falls: inventory of the ichthyofauna in the Iguassu River basin shows increasing percentages of nonnative species. Biota Neotrop. 21, e20201083. 10.1590/1676-0611-BN-2020-1083.

[cla70002-bib-0063] MMA – Ministério do Meio Ambiente , 2022. Portaria MMA No. 148, de 7 de junho de 2022. Available at: https://www.in.gov.br/en/web/dou/‐/portaria‐mma‐n‐148‐de‐7‐de‐junho‐de‐2022‐406272733 (accessed 9 March 2025).

[cla70002-bib-0064] Morais‐Silva, J.P. , Oliveira, A.V. , Fabrin, T.M.C. , Diamante, N.A. , Prioli, S.M.A.P. , Frota, A. , Graça, W.J. and Prioli, A.J. , 2018. Geomorphology influencing the diversification of fish in small‐order rivers of neighboring basins. Zebrafish 15, 389–397. 29653071 10.1089/zeb.2017.1551

[cla70002-bib-0065] Morrone, J.J. , 1994. On the identification of areas of endemism. Syst. Biol. 43, 438–441.

[cla70002-bib-0066] Morrone, J.J. , 2009. Evolutionary Biogeography: An Integrative Approach With Case Studies. Columbia University Press, New York.

[cla70002-bib-0067] Morrone, J.J. , 2014. Parsimony analysis of endemicity (PAE) revisited. J. Biogeogr. 41, 842–854.

[cla70002-bib-0068] Morrone, J.J. , 2018. The spectre of biogeographical regionalization. J. Biogeogr. 45, 282–288.

[cla70002-bib-0069] Munguía‐Lino, G. , Escalante, T. , Morrone, J.J. and Rodríguez, A. , 2016. Areas of endemism of the North American species of Tigridieae (Iridaceae). Aust. Syst. Bot. 29, 142–156.

[cla70002-bib-0070] Myers, N. , Mittermeier, R.A. , Mittermeier, C.G. , Fonseca, G.A.B. and Kent, J. , 2000. Biodiversity hotspots for conservation priorities. Nature 403, 853–858. 10706275 10.1038/35002501

[cla70002-bib-0071] Narváez‐Gómez, J.P. , Szumik, C.A. , Goloboff, P.A. and Lohmann, L.G. , 2022. Unravelling distribution patterns of Neotropical lianas: an analysis of endemicity of tribe Bignonieae (Bignoniaceae). Bot. J. Linn. Soc. 199, 470–495.

[cla70002-bib-0072] Nogueira, C. , Buckup, P.A. , Menezes, N.A. , Oyakawa, O.T. , Kasecker, T.P. , Ramos Neto, M.B. and da Silva, J.M.C. , 2010. Restricted‐range fishes and the conservation of Brazilian freshwaters. PLoS One 5, e11390. 10.1371/journal.pone.0011390. 20613986 PMC2894945

[cla70002-bib-0073] Ocampo‐Salinas, J.M. , Castillo‐Cerón, J.M. , Manríquez‐Morán, N. , Goyenechea, I. and Casagranda, M.D. , 2019. Endemism of lizards in the Chihuahuan Desert province: An approach based on endemicity analysis. J. Arid Environ. 163, 9–17.

[cla70002-bib-0074] Ota, R.R. , Deprá, G.C. , Graça, W.J. and Pavanelli, C.S. , 2018. Peixes da planície de inundação do alto rio Paraná e áreas adjacentes: revised, annotated and updated. Neotrop. Ichthyol. 16, e170094. 10.1590/1982-0224-20170094.

[cla70002-bib-0075] Oyakawa, O.T. , Akama, A. , Mautari, K.C. and Nolasco, J.C. , 2006. Peixes de riachos da Mata Atlântica. Neotrópica, São Paulo.

[cla70002-bib-0076] Pacifico, R. , Almeda, F. , Frota, A. and Fidanza, K. , 2020. Areas of endemism on Brazilian mountaintops revealed by taxonomically verified records of Microlicieae (Melastomataceae). Phytotaxa 450, 119–148.

[cla70002-bib-0077] Paraná – Governo do Estado , 2024. Decreto No 6.040. Reconhece as espécies da fauna ameaçada de extinção no Estado do Paraná e dá outras providências. Available at: https://maternatura.org.br/wp‐content/uploads/2024/06/EX_2024‐06‐05.pdf (accessed 9 March 2025).

[cla70002-bib-0078] Paraná – Secretaria do Estado do Meio Ambiente e Recursos Hídricos, SEMA , 2010. Bacias hidrográficas do Paraná. Série Histórica. SEMA, Curitiba.

[cla70002-bib-0079] Platnick, N.I. , 1991. On areas of endemism. Austr. Syst. Bot. 4, xi–xii.

[cla70002-bib-0080] Prado, J.R. , Brennand, P.G.G. , Godoy, L.P. , Libardi, G.S. , de Abreu‐Júnior, E.F. , Roth, P.R.O. , Chiquito, E.A. and Percequillo, A.R. , 2015. Species richness and areas of endemism of oryzomyine rodents (Cricetidae, Sigmodontinae) in South America: an NDM/VNDM approach. J. Biogeogr. 42, 540–551.

[cla70002-bib-0081] Reis, R.E. , Albert, J.S. , Di Dario, F. , Mincarone, M.M. , Petry, P. and Rocha, L.A. , 2016. Fish biodiversity and conservation in South America. J. Fish Biol. 89, 12–47. 27312713 10.1111/jfb.13016

[cla70002-bib-0082] Reis, R.B. , Frota, A. , Deprá, G.C. , Ota, R.R. and Graça, W.J. , 2020. Freshwater fishes from Paraná State, Brazil: An annotated list, with comments on biogeographic patterns, threats, and future perspectives. Zootaxa 4868, 451–494. 10.11646/zootaxa.4868.4.1 33311378

[cla70002-bib-0083] Reis, R.B. , Stabile, B.H.M. , Frota, A. , Ferrer, J. , Oliveira, A.V. and Graça, W.J. , 2025. Recent dispersion routes between freshwater ecoregions evidence headwater captures in southern Brazil: a case study using cryptic species of the Neotropical freshwater fish genus *Cambeva* (Siluriformes: Trichomycteridae). Hydrobiologia 852, 852–873.

[cla70002-bib-0084] Ribeiro, A.C. , 2006. Tectonic history and the biogeography of the freshwater fishes from the coastal drainages of eastern Brazil: An example of faunal evolution associated with a divergent continental margin. Neotrop. Ichthyol. 4, 225–246.

[cla70002-bib-0085] Richardson, D.M. and Whittaker, R.J. , 2010. Conservation biogeography foundations, concepts and challenges. Divers. Distrib. 16, 313–320.

[cla70002-bib-0086] Rodewald, A.D. , Strimas‐Mackey, M. , Schuster, R. and Arcese, P. , 2019. Tradeoffs in the value of biodiversity feature and cost data in conservation prioritization. Sci. Rep. 9, 15921. 10.1038/s41598-019-52241-2. 31685869 PMC6828800

[cla70002-bib-0087] Santos, C.M.D. and Fuhlendorf, M. , 2019. GeX: An automated tool for generating XYD files for analysis of endemicity using VNDM. Cladistics 35, 125–129. 34622979 10.1111/cla.12236

[cla70002-bib-0088] Schultz, E.D. and Cracraft, J. , 2024. Rethinking spatial history: envisioning a mechanistic historical biogeography. Cladistics 40, 653–662. 39340473 10.1111/cla.12598

[cla70002-bib-0089] Sigrist, M.S. and Carvalho, C.J.B. , 2008. Detection of areas of endemism on two spatial scales using parsimony analysis of endemicity (PAE): the Neotropical region and the Atlantic Forest. Biota Neotrop. 8, 33–42.

[cla70002-bib-0090] Smith, R.J. , Cartwright, S.J. , Fairbairn, A.C. , Lewis, D.C. , Gibbon, G.E.M. , Stewart, C.L. , Sykes, R.E. and Addison, P.F.E. , 2022. Developing a nature recovery network using systematic conservation planning. Conserv. Sci. Pract. 4, e578. 10.1111/csp2.578.

[cla70002-bib-0091] Strahler, A.N. , 1957. Quantitative analysis of watershed geomorphology. Eos. Trans. AGU 38, 913–920.

[cla70002-bib-0092] Szumik, C. and Goloboff, P.A. , 2004. Areas of endemism: an improved optimality criterion. Syst. Biol. 53, 968–977. 15764564 10.1080/10635150490888859

[cla70002-bib-0093] Szumik, C. and Goloboff, P.A. , 2015. Higher taxa and the identification of areas of endemism. Cladistics 31, 568–572. 34753267 10.1111/cla.12112

[cla70002-bib-0094] Szumik, C.A. , Cuezzo, F. , Goloboff, P. and Chalup, A.E. , 2002. An optimality criterion to determine areas of endemism. Syst. Biol. 51, 806–816. 12396592 10.1080/10635150290102483

[cla70002-bib-0095] Szumik, C. , Pereyra, V.V. and Casagranda, M.D. , 2019. Areas of endemism: to overlap or not to overlap, that is the question. Cladistics 35, 198–229. 34622975 10.1111/cla.12343

[cla70002-bib-0096] Tedesco, P.A. , Beauchard, O. , Bigorne, R. , Blanchet, S. , Buisson, L. , Conti, L. , Cornu, J.F. , Dias, M.S. , Grenouillet, G. , Hugueny, B. , Jézéquel, C. , Leprieur, F. , Brosse, S. and Oberdorff, T. , 2017. A global database on freshwater fish species occurrence in drainage basins. Sci. Data 4, 170141. 10.1038/sdata.2017.141. 28972575 PMC5625552

[cla70002-bib-0097] Thomaz, A.T. , Christie, M.R. and Knowles, L.L. , 2016. The architecture of river networks can drive the evolutionary dynamics of aquatic populations. Evolution 70, 731–739. 26888211 10.1111/evo.12883

[cla70002-bib-0098] Tognelli, M.F. , Anderson, E.P. , Jiménez‐Segura, L.F. , Chuctaya, J. , Chocano, L. , Maldonado‐Ocampo, J.A. , Mesa‐Salazar, L. , Mojica, J.I. , Carvajal‐Vallejos, F.M. , Correa, V. , Ortega, H. , Rivadeneira Romero, J.F. , Sánchez‐Duarte, P. , Cox, N.A. , Hidalgo, M. , Jiménez Prado, P. , Lasso, C.A. , Sarmiento, J. , Velásquez, M.A. and Villa‐Navarro, F.A. , 2019. Assessing conservation priorities of endemic freshwater fishes in the Tropical Andes region. Aquat. Conserv. 29, 1123–1132.

[cla70002-bib-0099] Tumini, G. , Giri, F. , Williner, V. , Collins, P.A. and Morrone, J.J. , 2019. Selecting and ranking areas for conservation of *Aegla* (Crustacea: Decapoda: Anomura) in southern South America integrating biogeography, phylogeny and assessments of extinction risk. Aquat. Conserv. 29, 693–705.

