# Supplementary material for: Establishing new grid‐size‐dependent attributes to rank areas of endemism for conservation priorities
Source: Cladistics. 2025 Jun 19;41(5):493–512. doi: 10.1111/cla.70002 (PMC12466105; doi:10.1111/cla.70002)
Supplement: Supplementary file 1 — Figs. S1–S153. Figures showing all Areas of Endemism (AEs) recovered for all grid sizes by the NDM/VNDM software. [file CLA-41-493-s004.pdf]

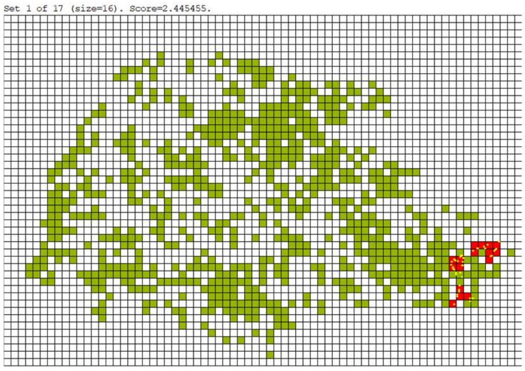

Fig. S1. Area of Endemism (AE = 1) recovered for 0.1º x 0.1º of grid size.

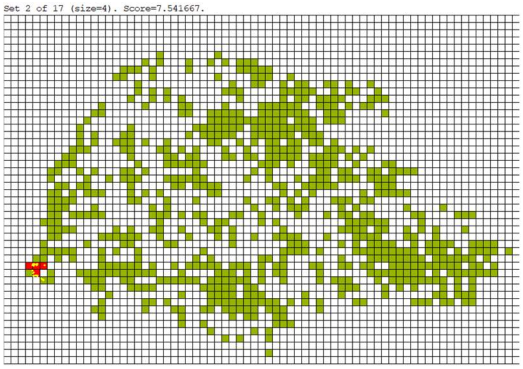

Fig. S2. Area of Endemism (AE = 2) recovered for 0.1º x 0.1º of grid size.

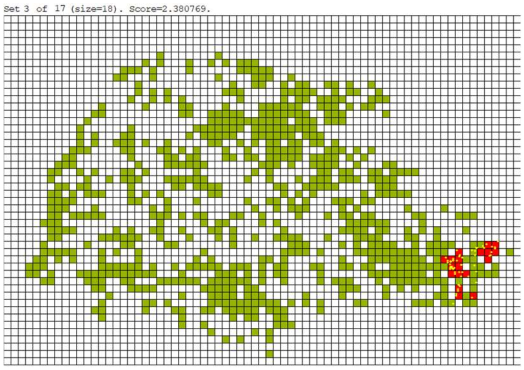

Fig. S3. Area of Endemism (AE = 3) recovered for 0.1º x 0.1º of grid size.

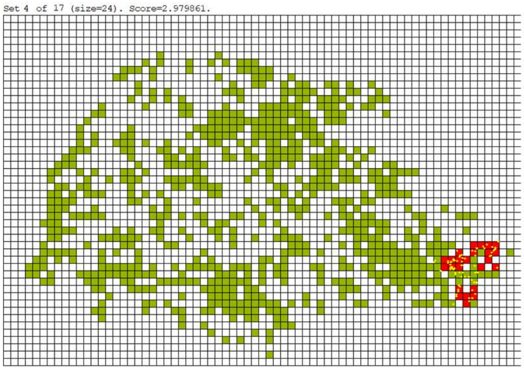

Fig. S4. Area of Endemism (AE = 4) recovered for 0.1º x 0.1º of grid size.

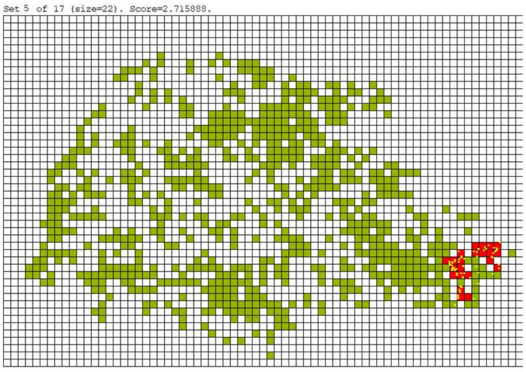

Fig. S5. Area of Endemism (AE = 5) recovered for 0.1º x 0.1º of grid size.

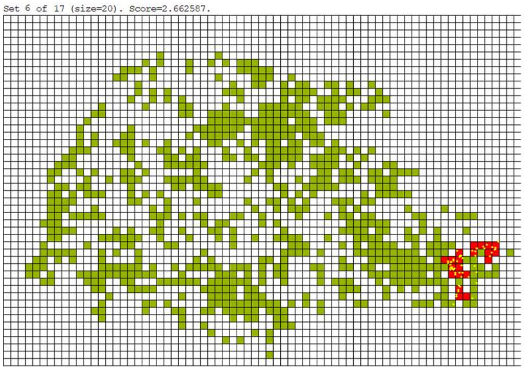

Fig. S6. Area of Endemism (AE = 6) recovered for 0.1º x 0.1º of grid size.

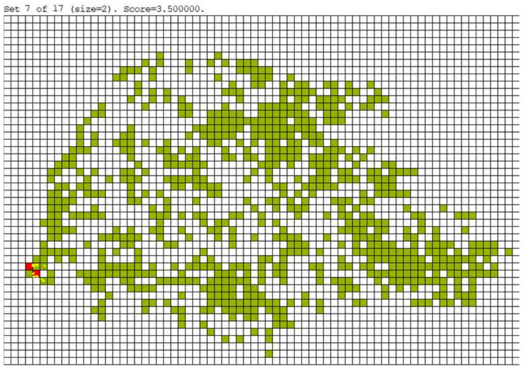

Fig. S7. Area of Endemism (AE = 7) recovered for 0.1º x 0.1º of grid size.

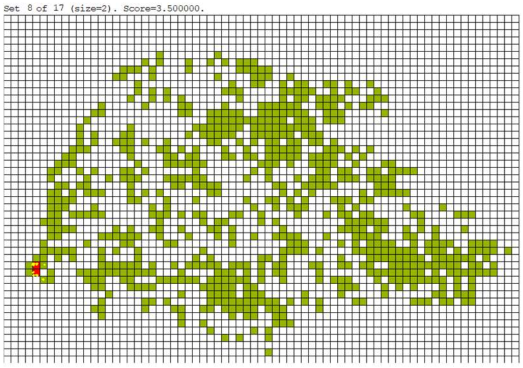

Fig. S8. Area of Endemism (AE = 8) recovered for 0.1º x 0.1º of grid size.

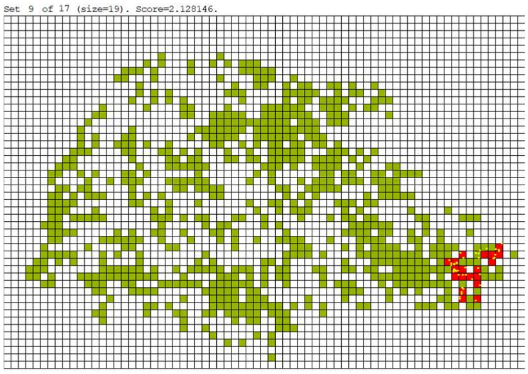

Fig. S9. Area of Endemism (AE = 9) recovered for 0.1º x 0.1º of grid size.

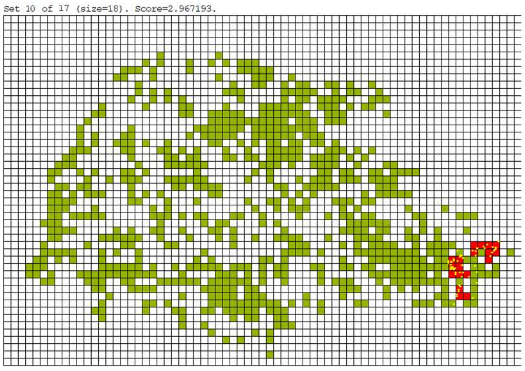

Fig. S10. Area of Endemism (AE = 10) recovered for 0.1º x 0.1º of grid size.

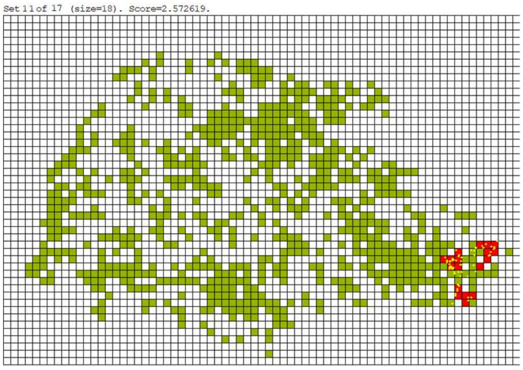

Fig. S11. Area of Endemism (AE = 11) recovered for 0.1º x 0.1º of grid size.

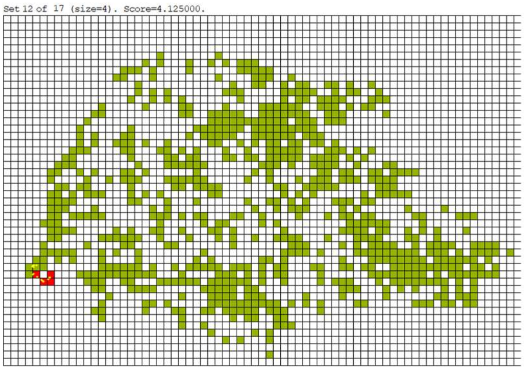

Fig. S12. Area of Endemism (AE = 12) recovered for 0.1º x 0.1º of grid size.

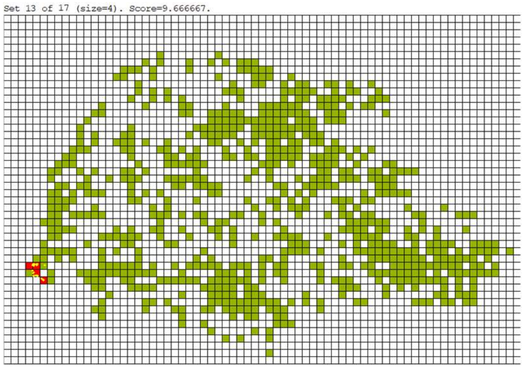

Fig. S13. Area of Endemism (AE = 13) recovered for 0.1º x 0.1º of grid size.

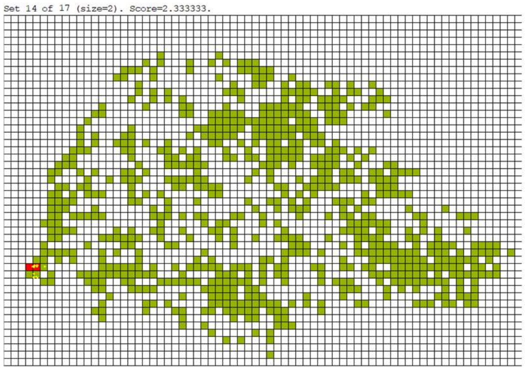

Fig. S14. Area of Endemism (AE = 14) recovered for 0.1º x 0.1º of grid size.

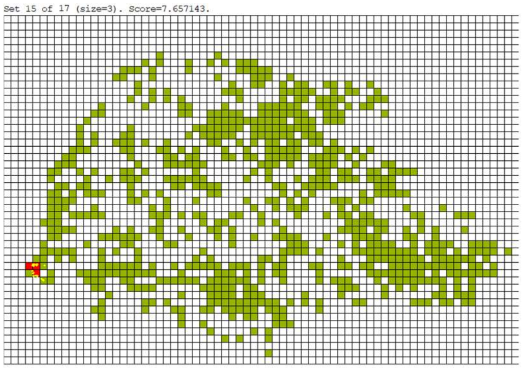

Fig. S15. Area of Endemism (AE = 15) recovered for 0.1º x 0.1º of grid size.

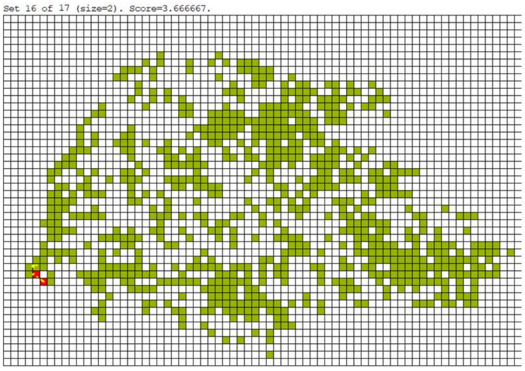

Fig. S16. Area of Endemism (AE = 16) recovered for 0.1º x 0.1º of grid size.

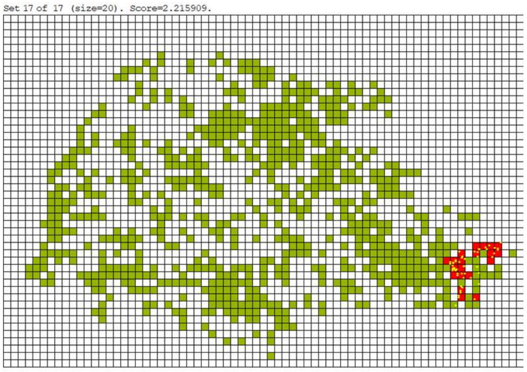

Fig. S17. Area of Endemism (AE = 17) recovered for 0.1º x 0.1º of grid size.

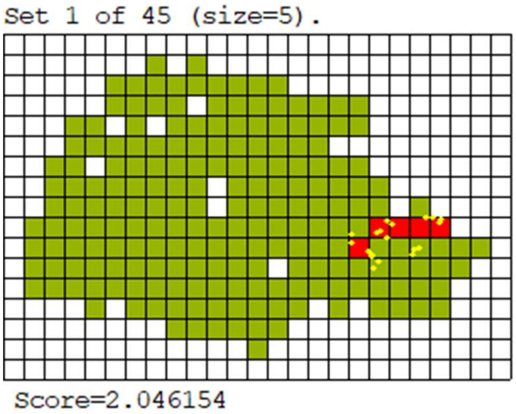

Fig. S18. Area of Endemism (AE = 1) recovered for 0.3º x 0.3º of grid size.

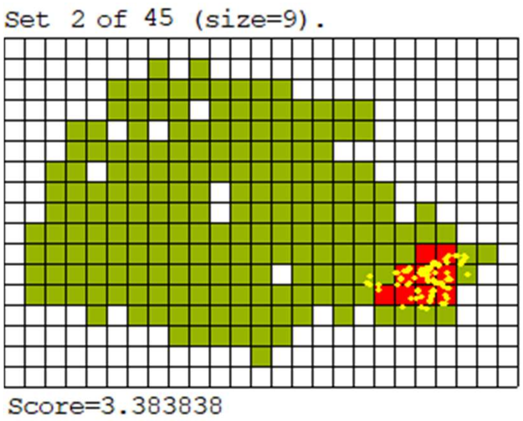

Fig. S19. Area of Endemism (AE = 2) recovered for 0.3º x 0.3º of grid size.

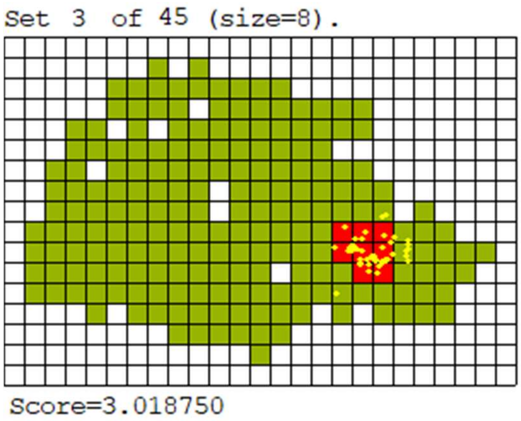

Fig. S20. Area of Endemism (AE = 3) recovered for 0.3º x 0.3º of grid size.

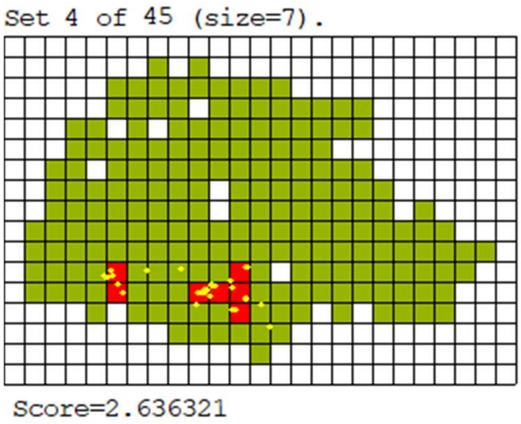

Fig. S21. Area of Endemism (AE = 4) recovered for 0.3º x 0.3º of grid size.

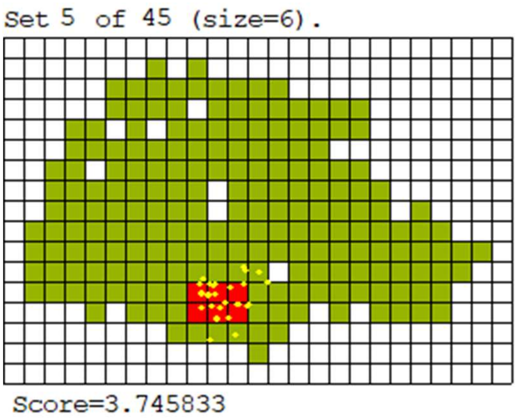

Fig. S22. Area of Endemism (AE = 5) recovered for 0.3º x 0.3º of grid size.

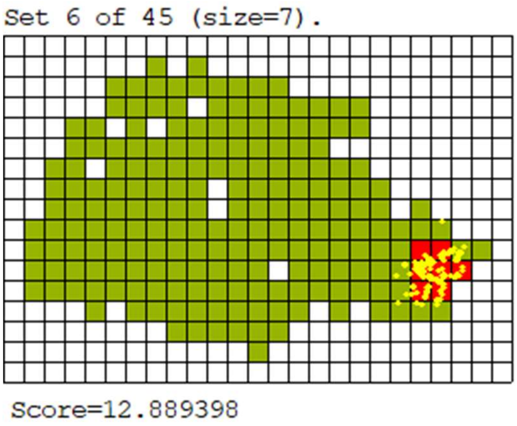

Fig. S23. Area of Endemism (AE = 6) recovered for 0.3º x 0.3º of grid size.

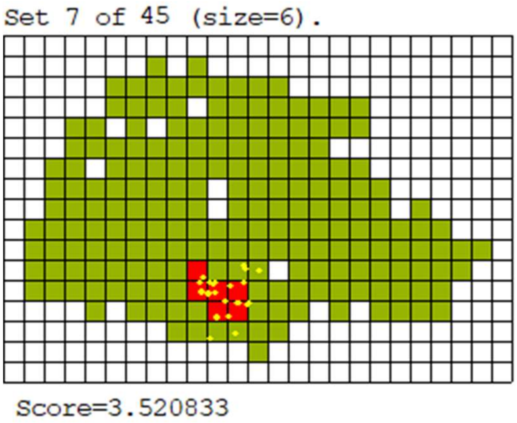

Fig. S24. Area of Endemism (AE = 7) recovered for 0.3º x 0.3º of grid size.

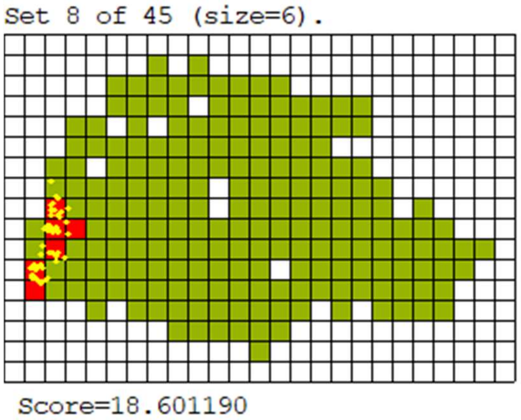

Fig. S25. Area of Endemism (AE = 8) recovered for 0.3º x 0.3º of grid size.

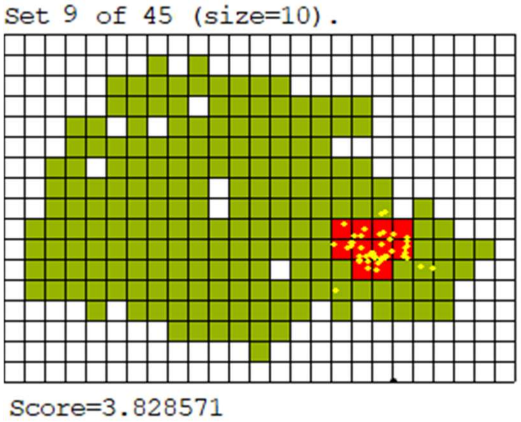

Fig. S26. Area of Endemism (AE = 9) recovered for 0.3º x 0.3º of grid size.

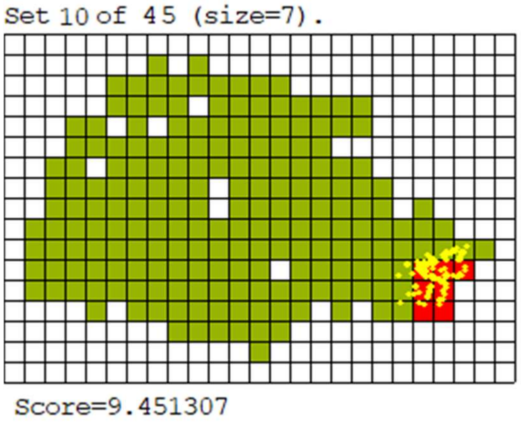

Fig. S27. Area of Endemism (AE = 10) recovered for 0.3º x 0.3º of grid size.

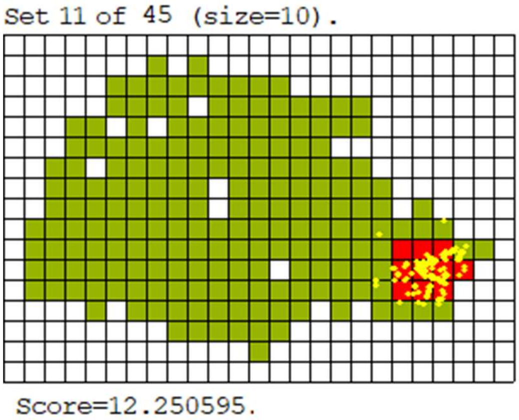

Fig. S28. Area of Endemism (AE = 11) recovered for 0.3º x 0.3º of grid size.

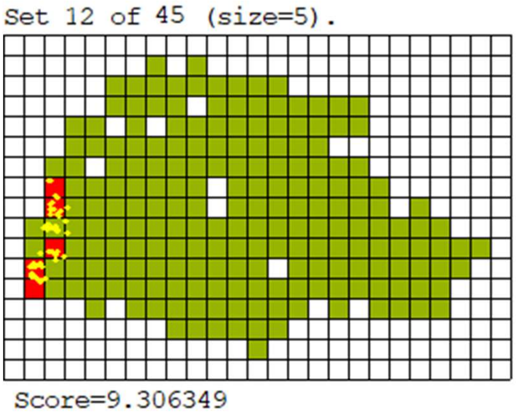

Fig. S29. Area of Endemism (AE = 12) recovered for 0.3º x 0.3º of grid size.

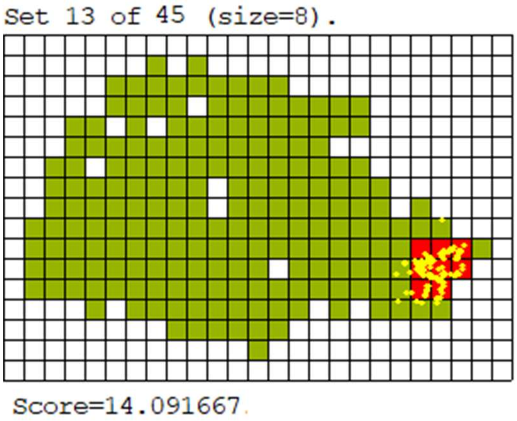

Fig. S30. Area of Endemism (AE = 13) recovered for 0.3º x 0.3º of grid size.

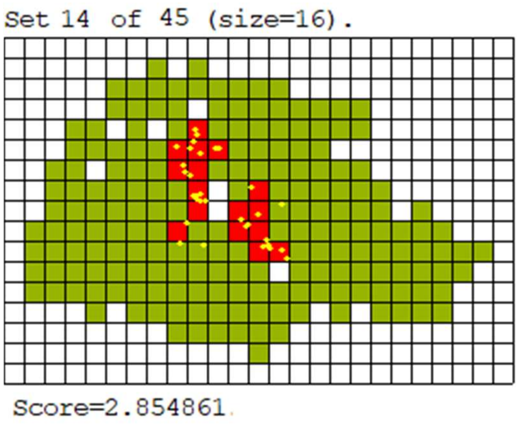

Fig. S31. Area of Endemism (AE = 14) recovered for 0.3º x 0.3º of grid size.

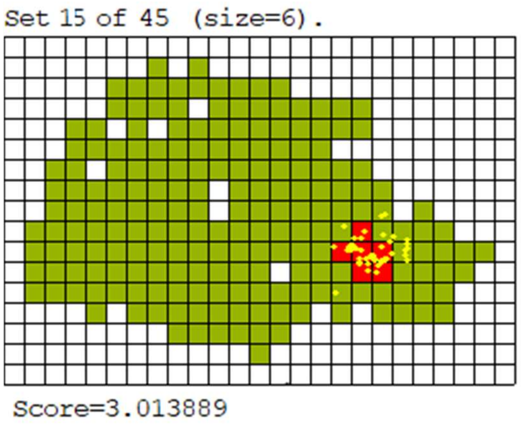

Fig. S32. Area of Endemism (AE = 15) recovered for 0.3º x 0.3º of grid size.

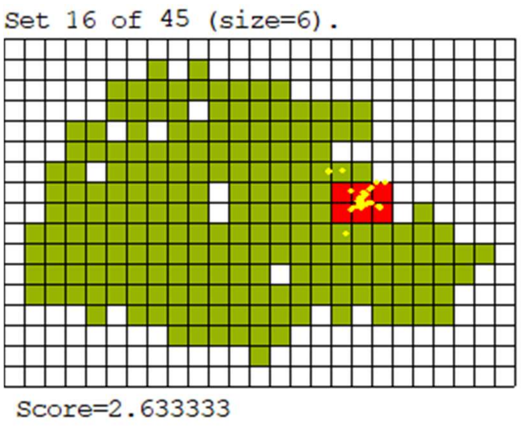

Fig. S33. Area of Endemism (AE = 16) recovered for 0.3º x 0.3º of grid size.

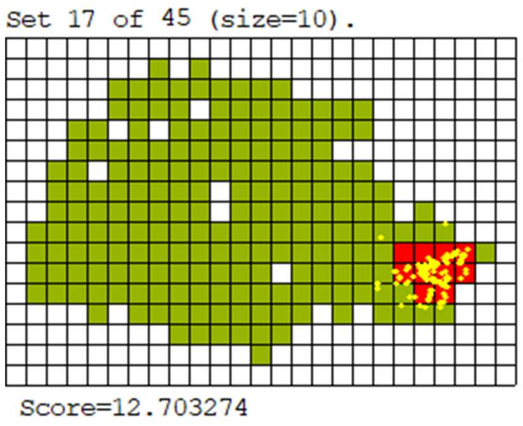

Fig. S34. Area of Endemism (AE = 17) recovered for 0.3º x 0.3º of grid size.

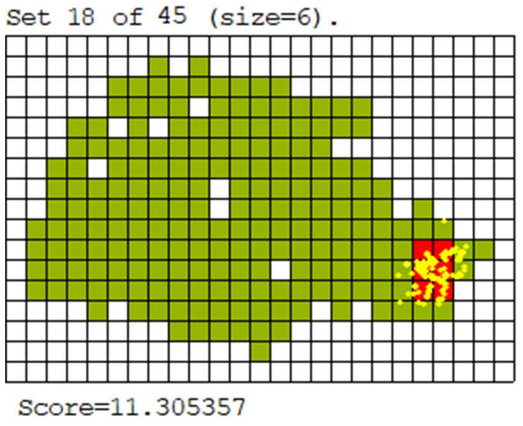

Fig. S35. Area of Endemism (AE = 18) recovered for 0.3º x 0.3º of grid size.

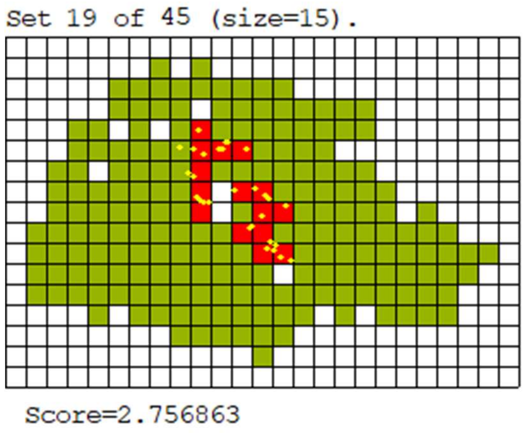

Fig. S36. Area of Endemism (AE = 19) recovered for 0.3º x 0.3º of grid size.

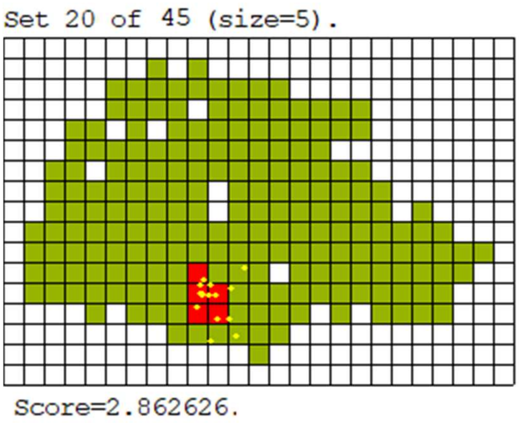

Fig. S37. Area of Endemism (AE = 20) recovered for 0.3° x 0.3° of grid size.

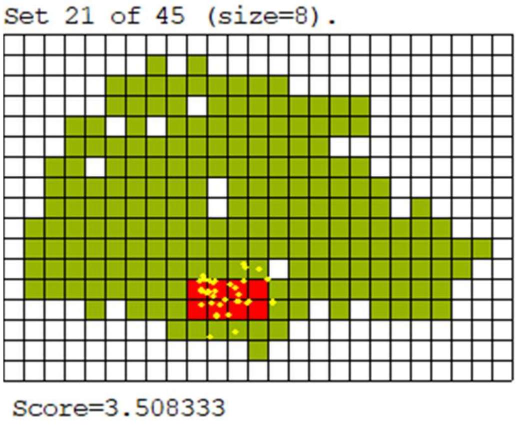

Fig. S38. Area of Endemism (AE = 21) recovered for 0.3° x 0.3° of grid size.

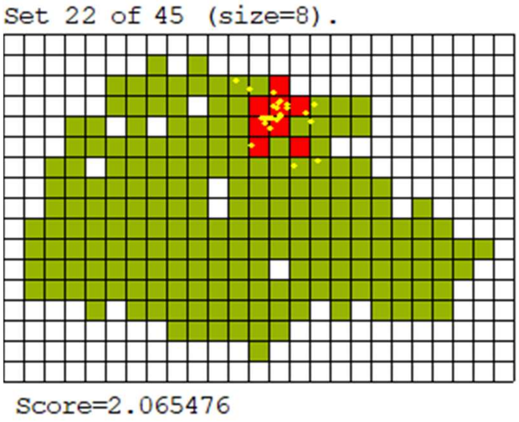

Fig. S39. Area of Endemism (AE = 22) recovered for 0.3° x 0.3° of grid size.

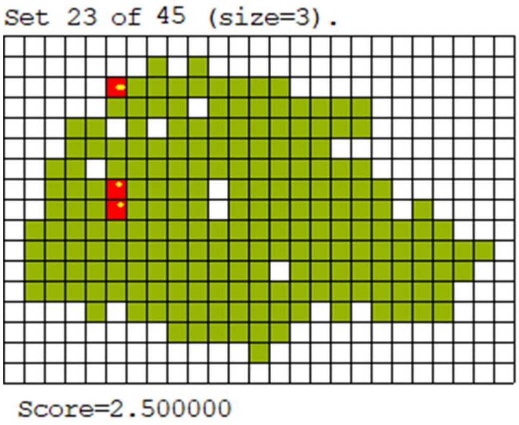

Fig. S40. Area of Endemism (AE = 23) recovered for 0.3° x 0.3° of grid size.

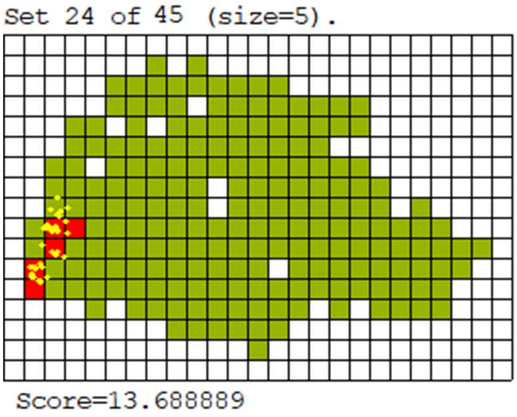

Fig. S41. Area of Endemism (AE = 24) recovered for 0.3° x 0.3° of grid size.

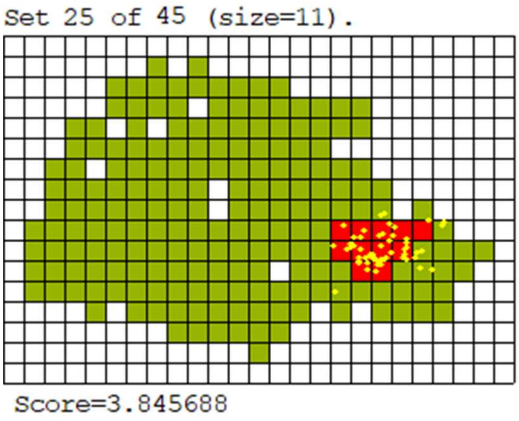

Fig. S42. Area of Endemism (AE = 25) recovered for 0.3° x 0.3° of grid size.

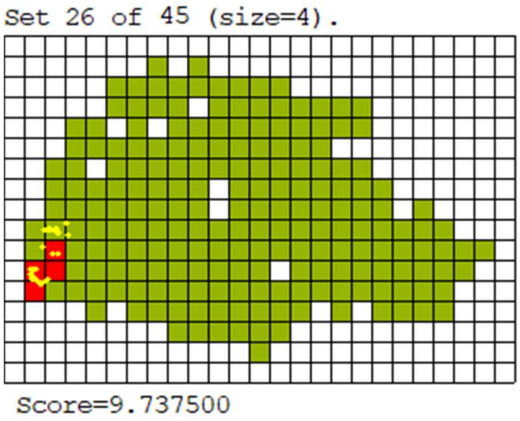

Fig. S43. Area of Endemism (AE = 26) recovered for 0.3º x 0.3º of grid size.

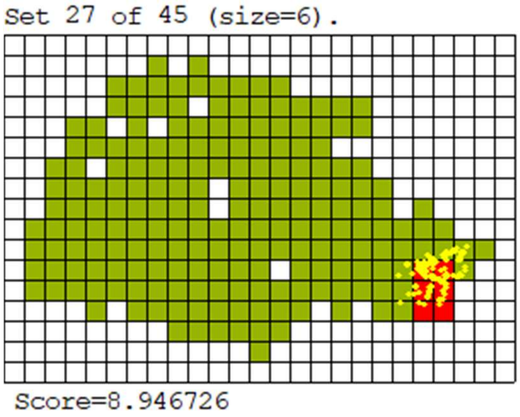

Fig. S44. Area of Endemism (AE = 27) recovered for 0.3º x 0.3º of grid size.

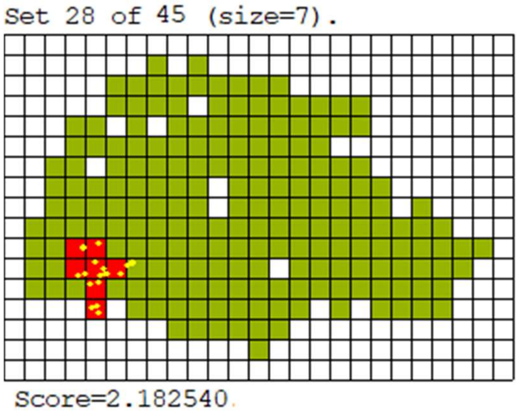

Fig. S45. Area of Endemism (AE = 28) recovered for 0.3º x 0.3º of grid size.

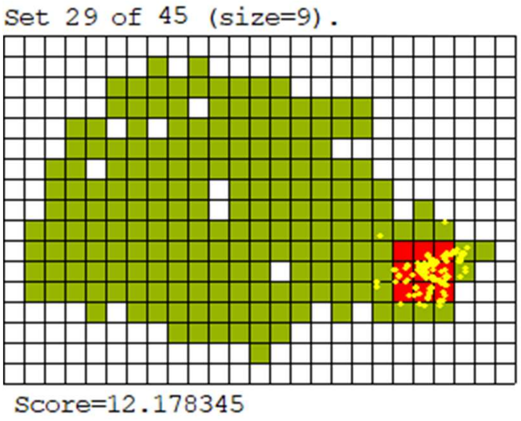

Fig. S46. Area of Endemism (AE = 29) recovered for 0.3º x 0.3º of grid size.

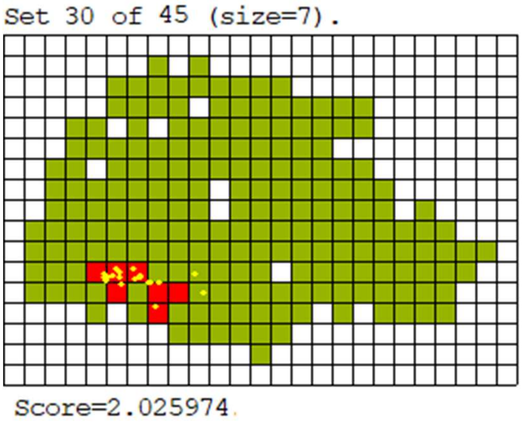

Fig. S47. Area of Endemism (AE = 30) recovered for 0.3º x 0.3º of grid size.

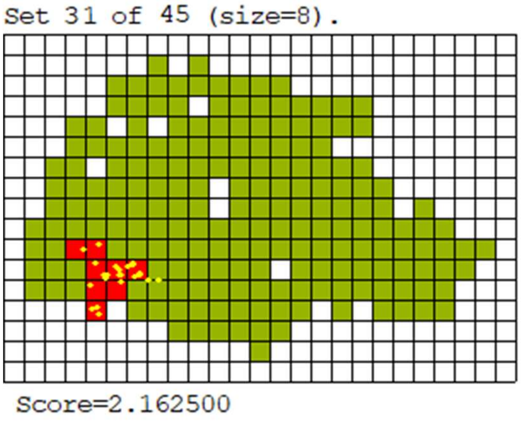

Fig. S48. Area of Endemism (AE = 31) recovered for 0.3º x 0.3º of grid size.

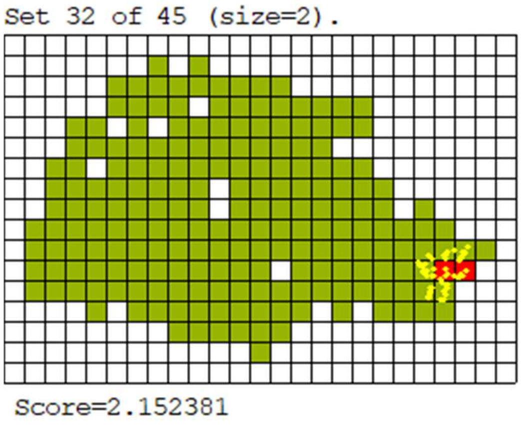

Fig. S49. Area of Endemism (AE = 32) recovered for 0.3º x 0.3º of grid size.

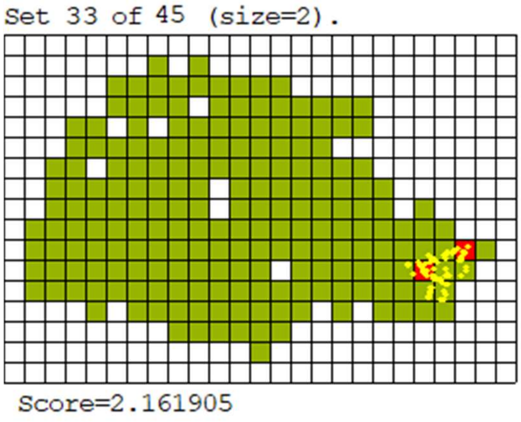

Fig. S50. Area of Endemism (AE = 33) recovered for 0.3º x 0.3º of grid size.

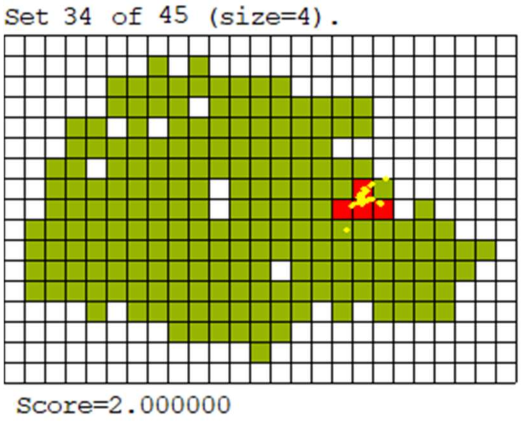

Fig. S51. Area of Endemism (AE = 34) recovered for 0.3º x 0.3º of grid size.

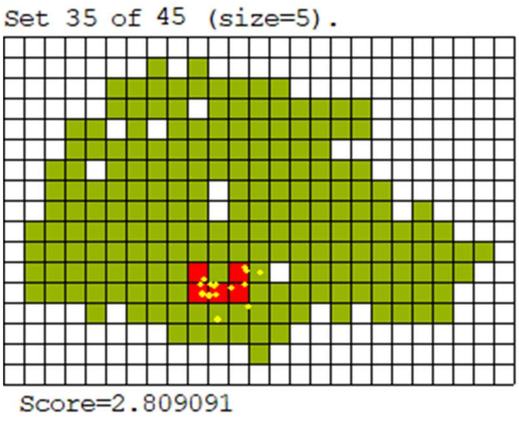

Fig. S52. Area of Endemism (AE = 35) recovered for 0.3º x 0.3º of grid size.

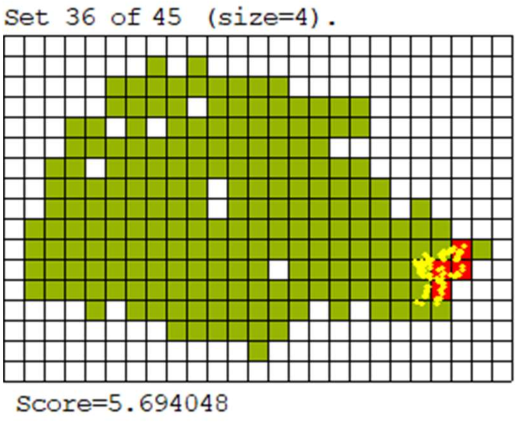

Fig. S53. Area of Endemism (AE = 36) recovered for 0.3º x 0.3º of grid size.

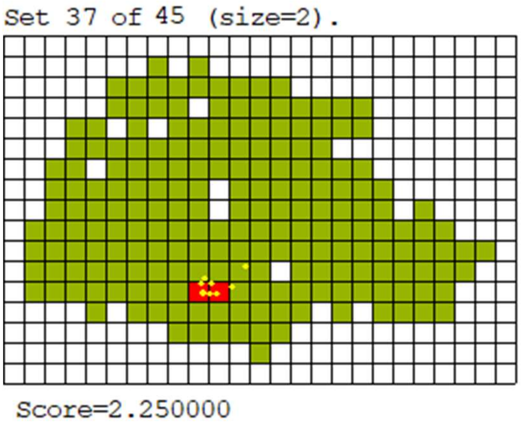

Fig. S54. Area of Endemism (AE = 37) recovered for 0.3º x 0.3º of grid size.

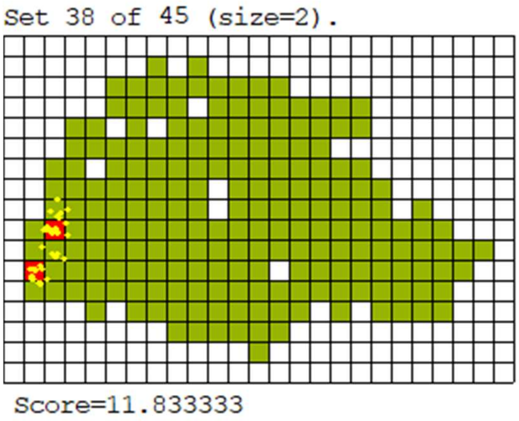

Fig. S55. Area of Endemism (AE = 38) recovered for 0.3º x 0.3º of grid size.

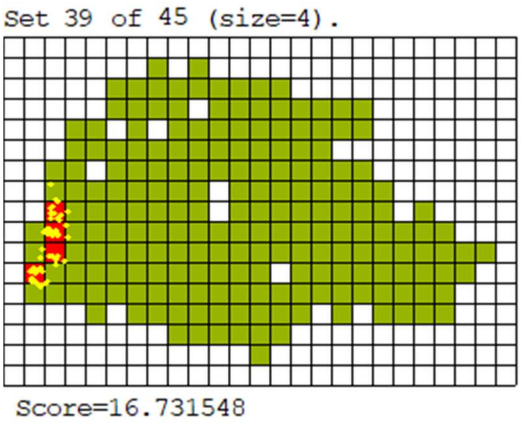

Fig. S56. Area of Endemism (AE = 39) recovered for 0.3º x 0.3º of grid size.

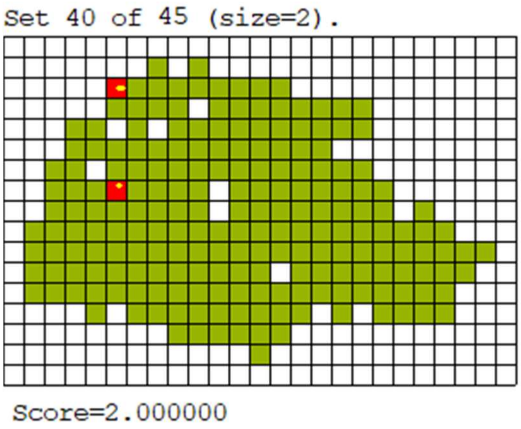

Fig. S57. Area of Endemism (AE = 40) recovered for 0.3º x 0.3º of grid size.

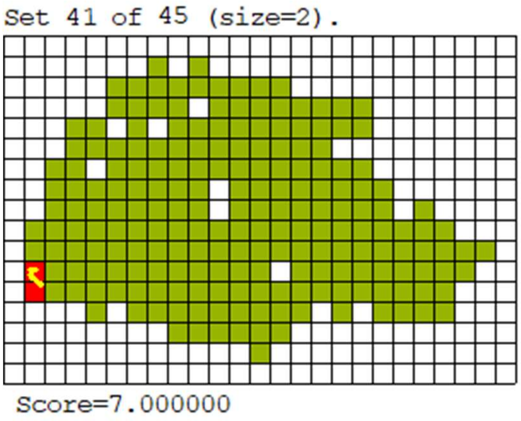

Fig. S58. Area of Endemism (AE = 41) recovered for 0.3º x 0.3º of grid size.

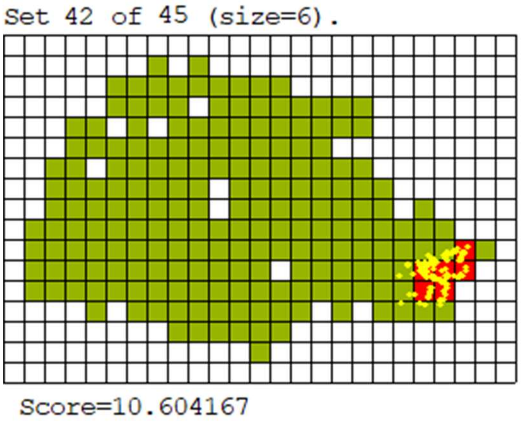

Fig. S59. Area of Endemism (AE = 42) recovered for 0.3º x 0.3º of grid size.

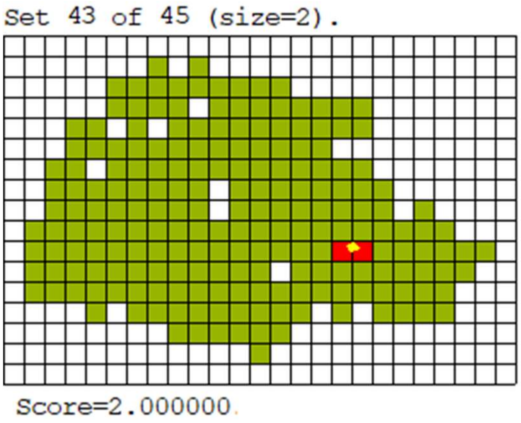

Fig. S60. Area of Endemism (AE = 43) recovered for 0.3º x 0.3º of grid size.

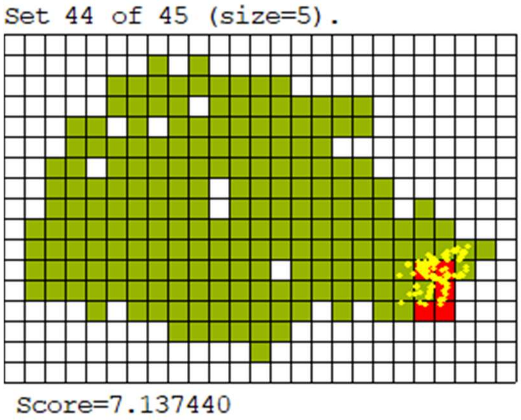

Fig. S61. Area of Endemism (AE = 44) recovered for 0.3º x 0.3º of grid size.

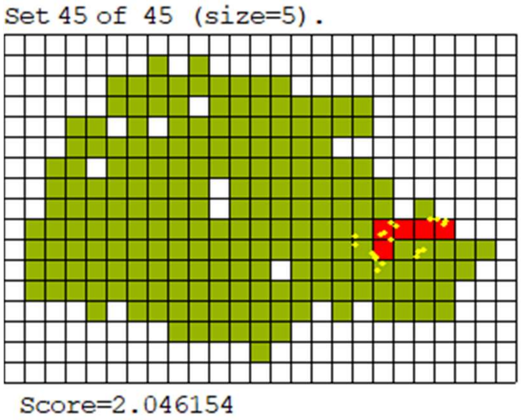

Fig. S62. Area of Endemism (AE = 45) recovered for 0.3º x 0.3º of grid size.

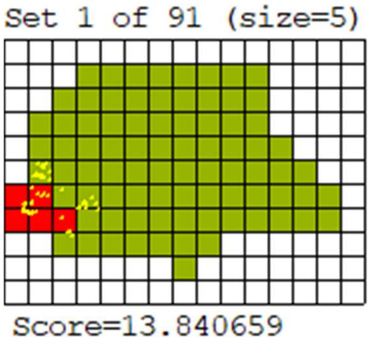

Fig. S63. Area of Endemism (AE = 1) recovered for 0.5º x 0.5º of grid size.

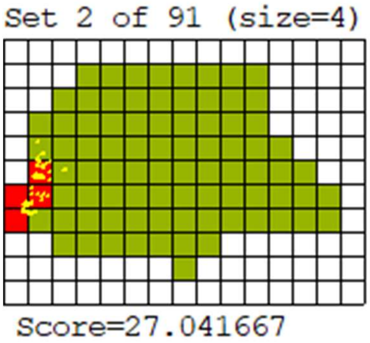

Fig. S64. Area of Endemism (AE = 2) recovered for 0.5º x 0.5º of grid size.

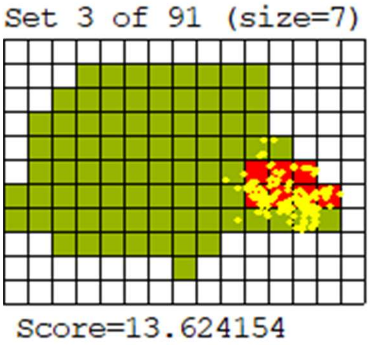

Fig. S65. Area of Endemism (AE = 3) recovered for 0.5º x 0.5º of grid size.

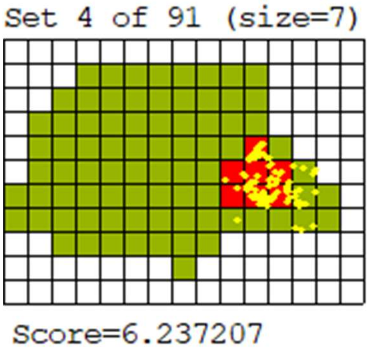

Fig. S66. Area of Endemism (AE = 4) recovered for 0.5º x 0.5º of grid size.

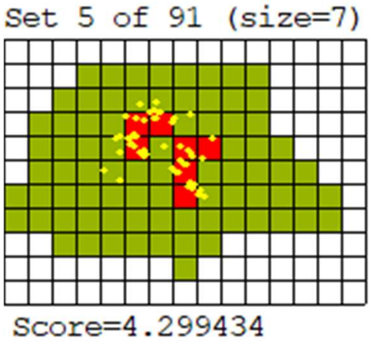

Fig. S67. Area of Endemism (AE = 5) recovered for 0.5º x 0.5º of grid size.

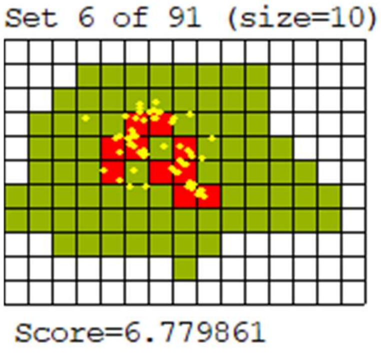

Fig. S68. Area of Endemism (AE = 6) recovered for 0.5º x 0.5º of grid size.

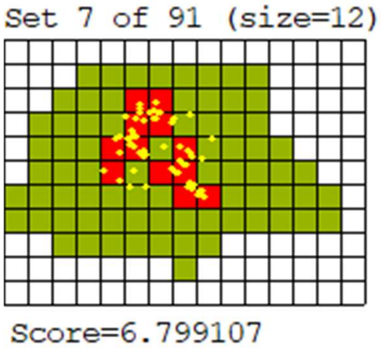

Fig. S69. Area of Endemism (AE = 7) recovered for 0.5º x 0.5º of grid size.

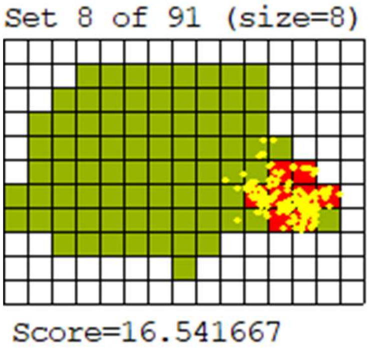

Fig. S70. Area of Endemism (AE = 8) recovered for 0.5º x 0.5º of grid size.

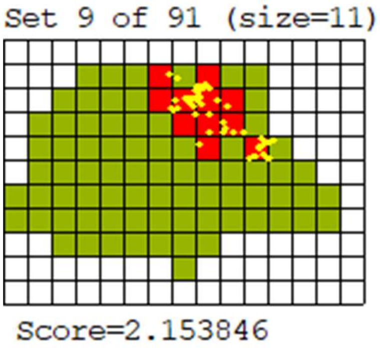

Fig. S71. Area of Endemism (AE = 9) recovered for 0.5º x 0.5º of grid size.

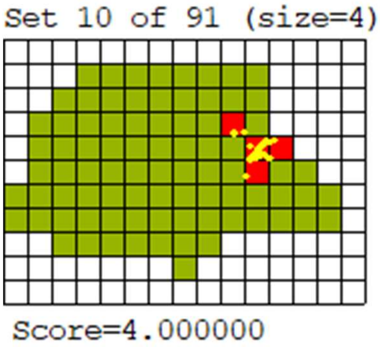

Fig. S72. Area of Endemism (AE = 10) recovered for 0.5º x 0.5º of grid size.

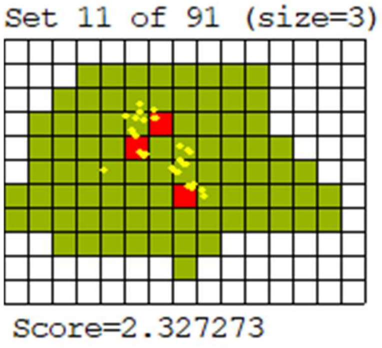

Fig. S73. Area of Endemism (AE = 11) recovered for 0.5º x 0.5º of grid size.

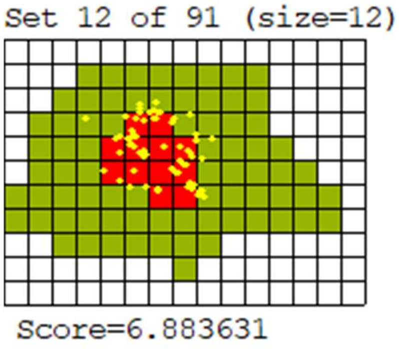

Fig. S74. Area of Endemism (AE = 12) recovered for 0.5º x 0.5º of grid size.

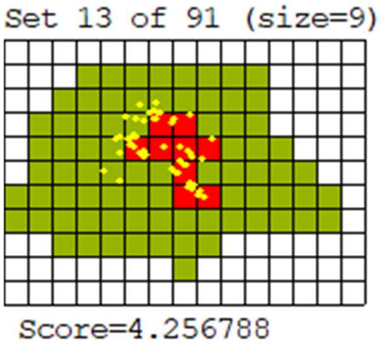

Fig. S75. Area of Endemism (AE = 13) recovered for 0.5º x 0.5º of grid size.

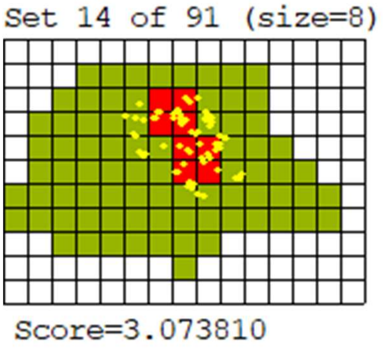

Fig. S76. Area of Endemism (AE = 14) recovered for 0.5º x 0.5º of grid size.

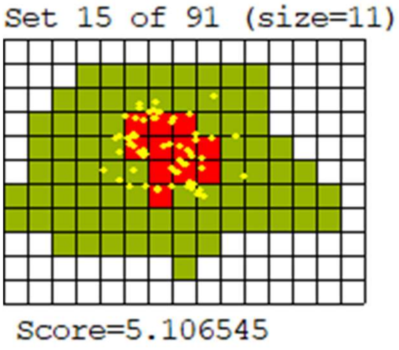

Fig. S77. Area of Endemism (AE = 15) recovered for 0.5º x 0.5º of grid size.

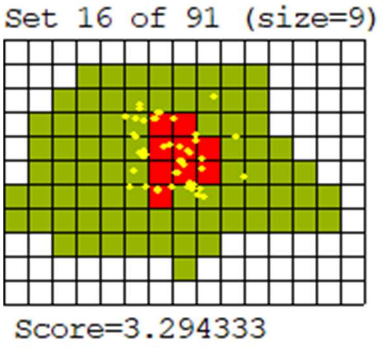

Fig. S78. Area of Endemism (AE = 16) recovered for 0.5º x 0.5º of grid size.

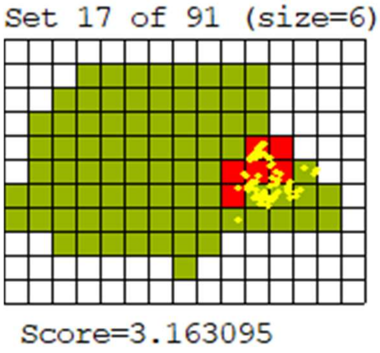

Fig. S79. Area of Endemism (AE = 17) recovered for 0.5º x 0.5º of grid size.

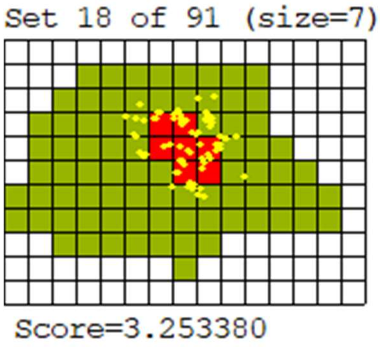

Fig. S80. Area of Endemism (AE = 18) recovered for 0.5º x 0.5º of grid size.

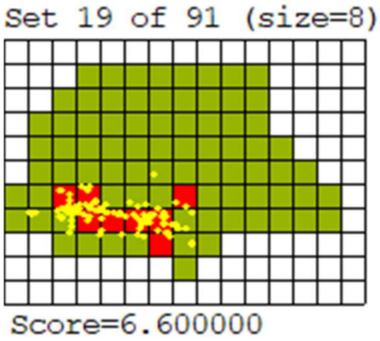

Fig. S81. Area of Endemism (AE = 19) recovered for 0.5º x 0.5º of grid size.

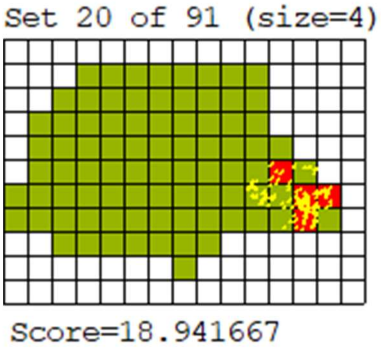

Fig. S82. Area of Endemism (AE = 20) recovered for 0.5º x 0.5º of grid size.

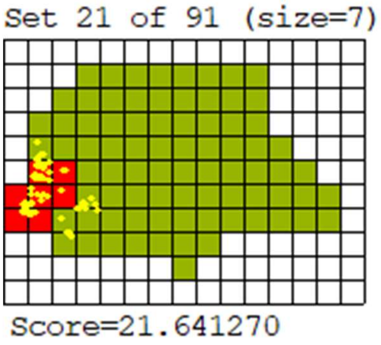

Fig. S83. Area of Endemism (AE = 21) recovered for 0.5º x 0.5º of grid size.

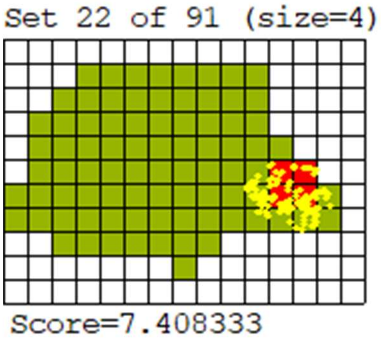

Fig. S84. Area of Endemism (AE = 22) recovered for 0.5º x 0.5º of grid size.

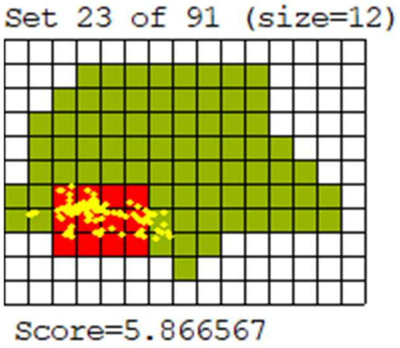

Fig. S85. Area of Endemism (AE = 23) recovered for 0.5º x 0.5º of grid size.

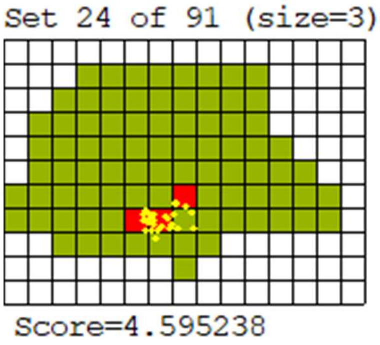

Fig. S86. Area of Endemism (AE = 24) recovered for 0.5º x 0.5º of grid size.

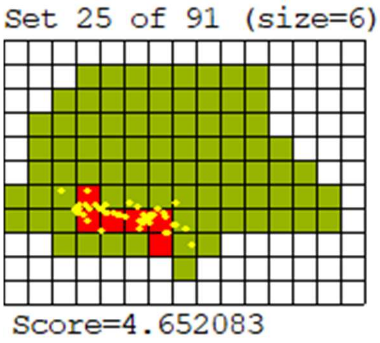

Fig. S87. Area of Endemism (AE = 25) recovered for 0.5º x 0.5º of grid size.

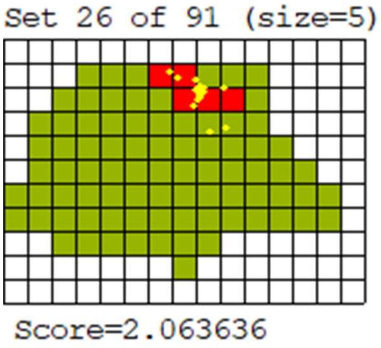

Fig. S88. Area of Endemism (AE = 26) recovered for 0.5º x 0.5º of grid size.

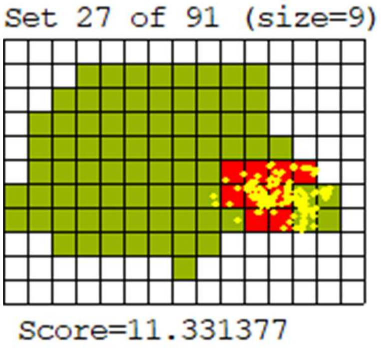

Fig. S89. Area of Endemism (AE = 27) recovered for 0.5º x 0.5º of grid size.

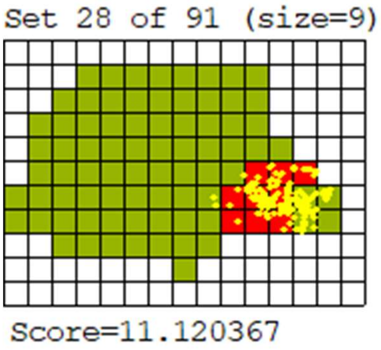

Fig. S90. Area of Endemism (AE = 28) recovered for 0.5º x 0.5º of grid size.

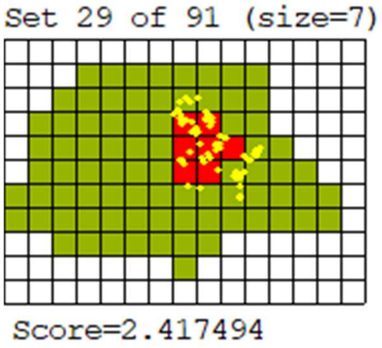

Fig. S91. Area of Endemism (AE = 29) recovered for 0.5º x 0.5º of grid size.

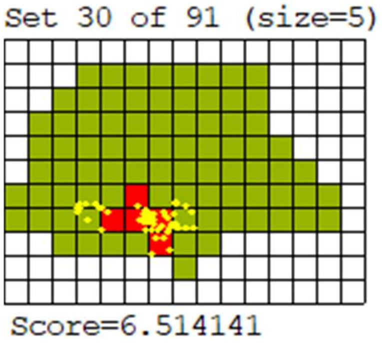

Fig. S92. Area of Endemism (AE = 30) recovered for 0.5º x 0.5º of grid size.

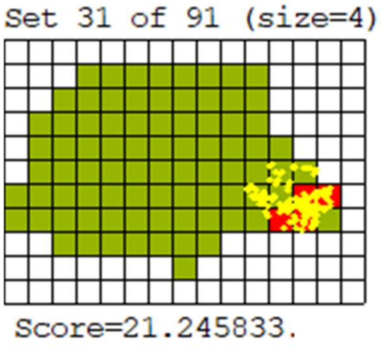

Fig. S93. Area of Endemism (AE = 31) recovered for 0.5º x 0.5º of grid size.

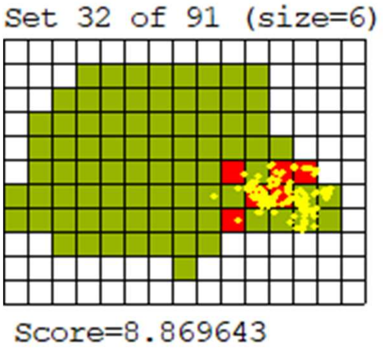

Fig. S94. Area of Endemism (AE = 32) recovered for 0.5º x 0.5º of grid size.

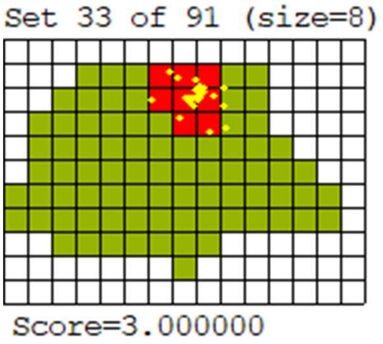

Fig. S95. Area of Endemism (AE = 33) recovered for 0.5º x 0.5º of grid size.

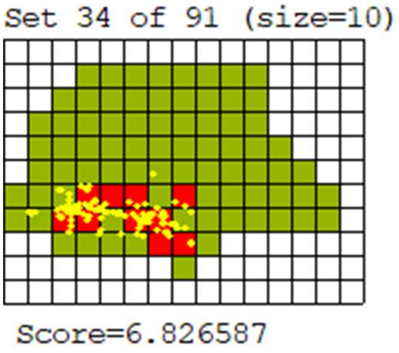

Fig. S96. Area of Endemism (AE = 34) recovered for 0.5º x 0.5º of grid size.

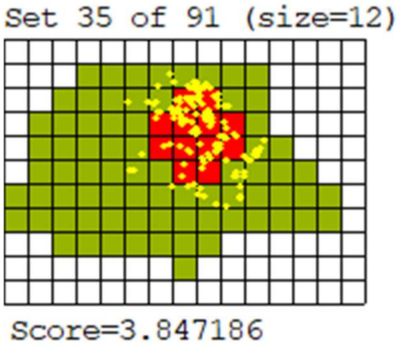

Fig. S97. Area of Endemism (AE = 35) recovered for 0.5º x 0.5º of grid size.

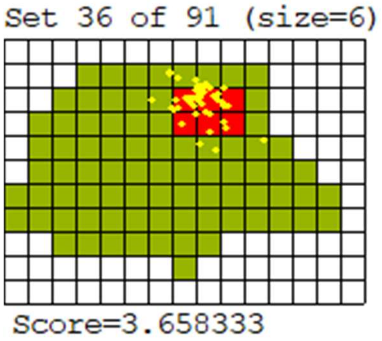

Fig. S98. Area of Endemism (AE = 36) recovered for 0.5º x 0.5º of grid size.

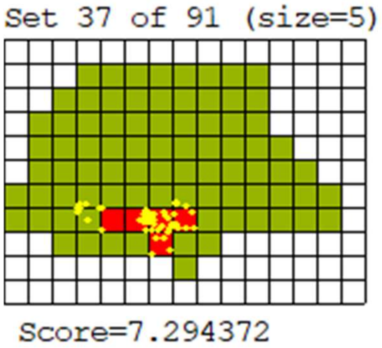

Fig. S99. Area of Endemism (AE = 37) recovered for 0.5º x 0.5º of grid size.

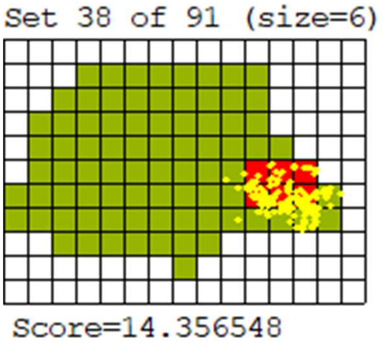

Fig. S100. Area of Endemism (AE = 38) recovered for 0.5º x 0.5º of grid size.

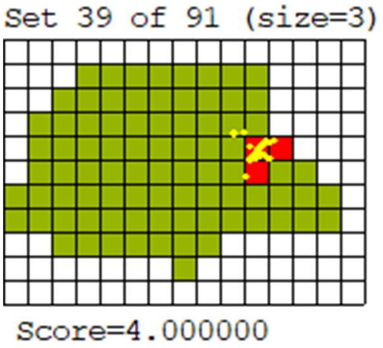

Fig. S101. Area of Endemism (AE = 39) recovered for 0.5º x 0.5º of grid size.

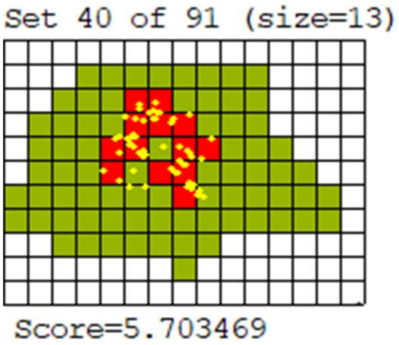

Fig. S102. Area of Endemism (AE = 40) recovered for 0.5º x 0.5º of grid size.

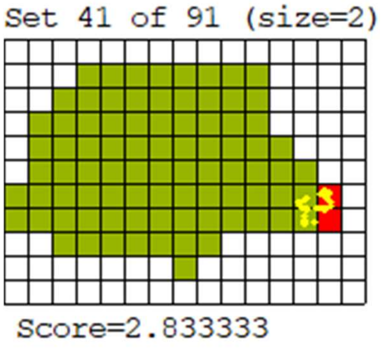

Fig. S103. Area of Endemism (AE = 41) recovered for 0.5º x 0.5º of grid size.

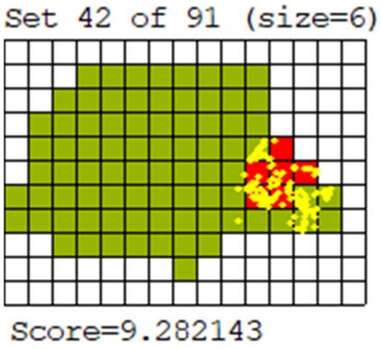

Fig. S104. Area of Endemism (AE = 42) recovered for 0.5º x 0.5º of grid size.

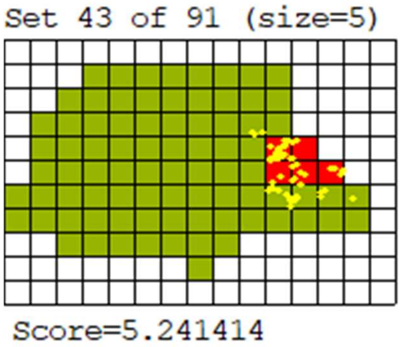

Fig. S105. Area of Endemism (AE = 43) recovered for 0.5º x 0.5º of grid size.

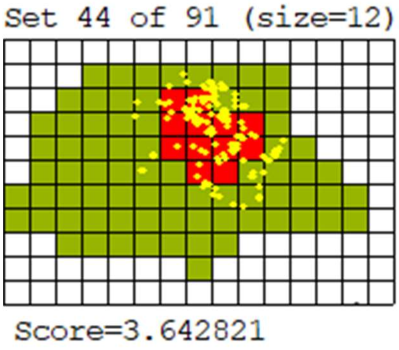

Fig. S106. Area of Endemism (AE = 44) recovered for 0.5º x 0.5º of grid size.

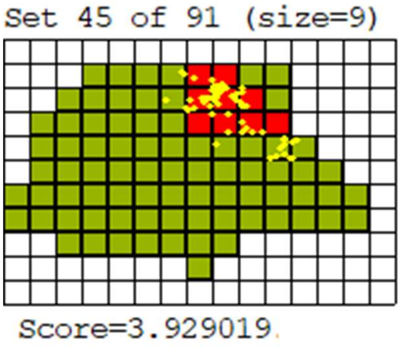

Fig. S107. Area of Endemism (AE = 45) recovered for 0.5º x 0.5º of grid size.

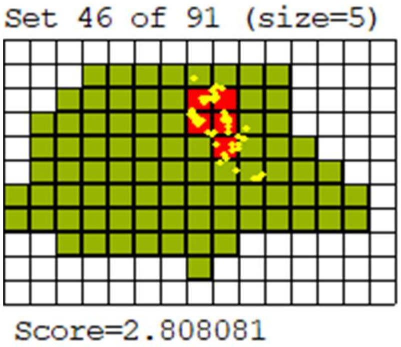

Fig. S108. Area of Endemism (AE = 46) recovered for 0.5º x 0.5º of grid size.

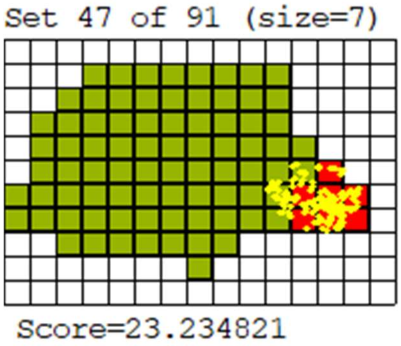

Fig. S109. Area of Endemism (AE = 47) recovered for 0.5º x 0.5º of grid size.

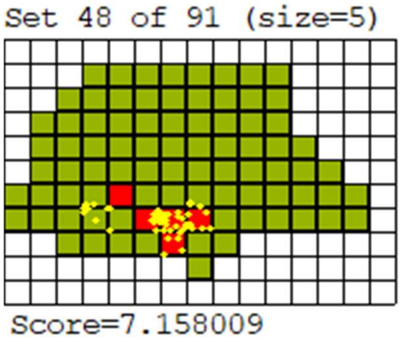

Fig. S110. Area of Endemism (AE = 48) recovered for 0.5º x 0.5º of grid size.

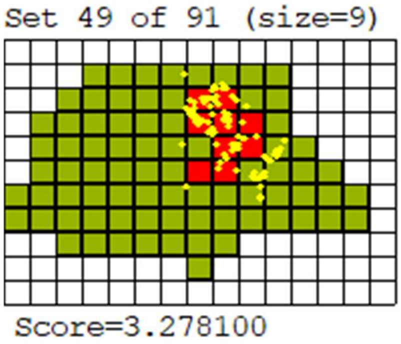

Fig. S111. Area of Endemism (AE = 49) recovered for 0.5º x 0.5º of grid size.

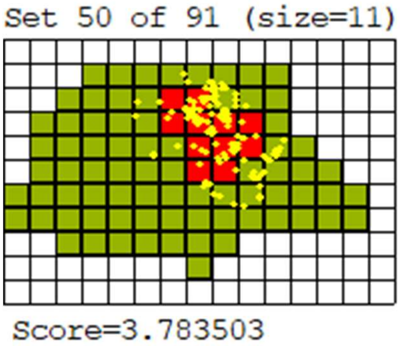

Fig. S112. Area of Endemism (AE = 50) recovered for 0.5º x 0.5º of grid size.

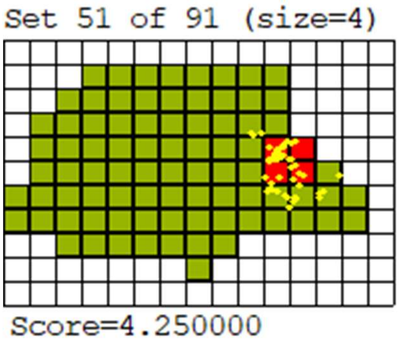

Fig. S113. Area of Endemism (AE = 51) recovered for 0.5º x 0.5º of grid size.

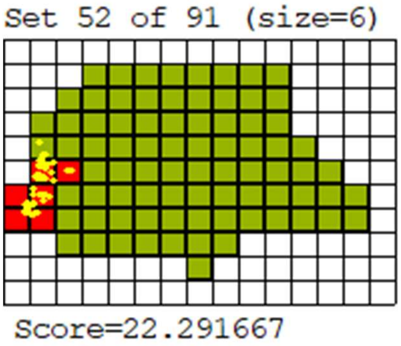

Fig. S114. Area of Endemism (AE = 52) recovered for 0.5º x 0.5º of grid size.

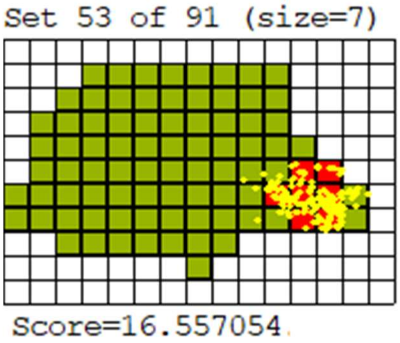

Fig. S115. Area of Endemism (AE = 53) recovered for 0.5º x 0.5º of grid size.

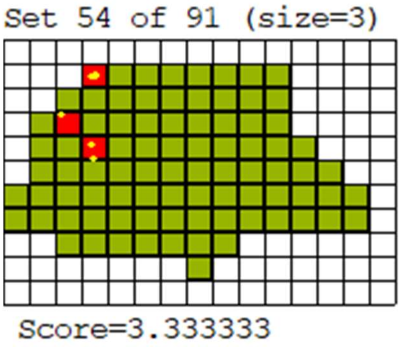

Fig. S116. Area of Endemism (AE = 54) recovered for 0.5º x 0.5º of grid size.

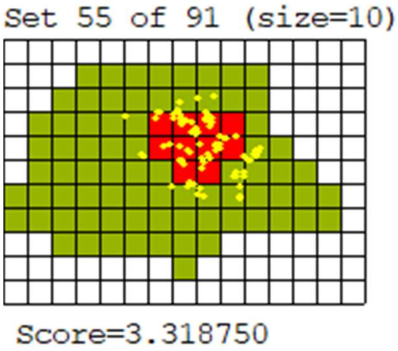

Fig. S117. Area of Endemism (AE = 55) recovered for 0.5º x 0.5º of grid size.

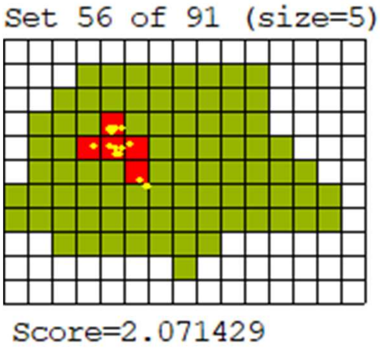

Fig. S118. Area of Endemism (AE = 56) recovered for 0.5º x 0.5º of grid size.

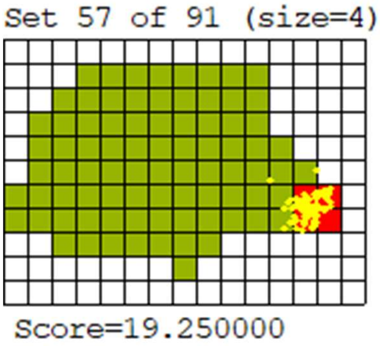

Fig. S119. Area of Endemism (AE = 57) recovered for 0.5º x 0.5º of grid size.

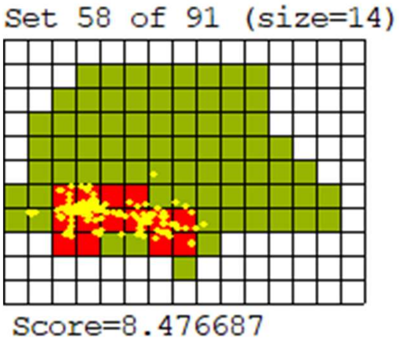

Fig. S120. Area of Endemism (AE = 58) recovered for 0.5º x 0.5º of grid size.

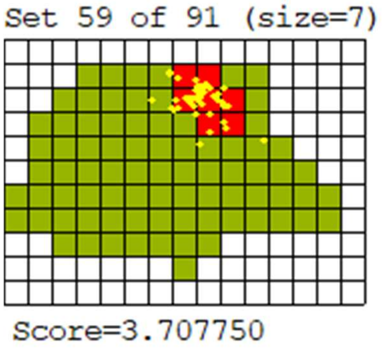

Fig. S121. Area of Endemism (AE = 59) recovered for 0.5º x 0.5º of grid size.

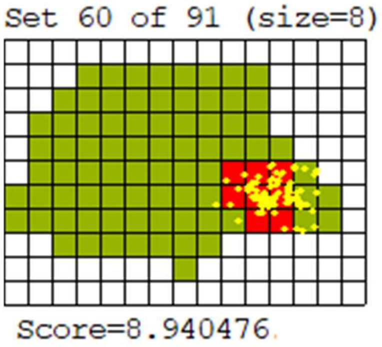

Fig. S122. Area of Endemism (AE = 60) recovered for 0.5º x 0.5º of grid size.

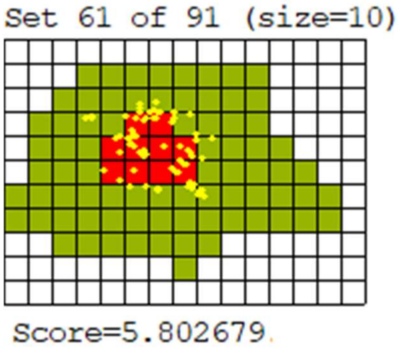

Fig. S123. Area of Endemism (AE = 61) recovered for 0.5º x 0.5º of grid size.

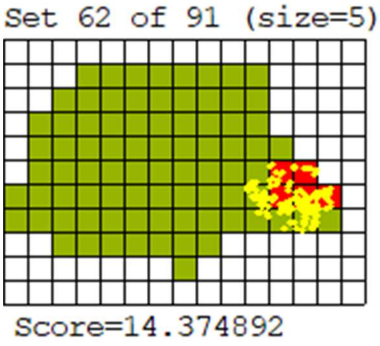

Fig. S124. Area of Endemism (AE = 62) recovered for 0.5º x 0.5º of grid size.

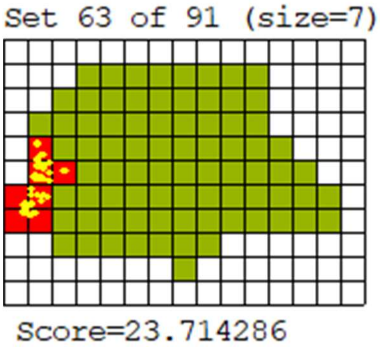

Fig. S125. Area of Endemism (AE = 63) recovered for 0.5º x 0.5º of grid size.

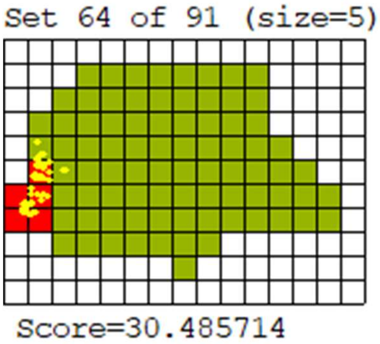

Fig. S126. Area of Endemism (AE = 64) recovered for 0.5º x 0.5º of grid size.

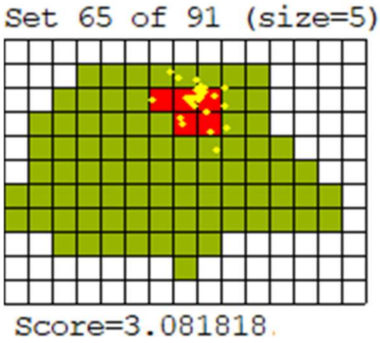

Fig. S127. Area of Endemism (AE = 65) recovered for 0.5º x 0.5º of grid size.

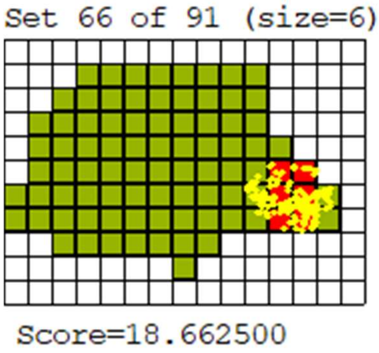

Fig. S128. Area of Endemism (AE = 66) recovered for 0.5º x 0.5º of grid size.

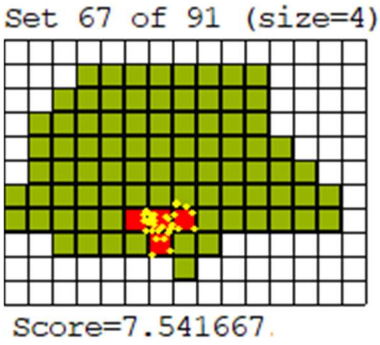

Fig. S129. Area of Endemism (AE = 67) recovered for 0.5º x 0.5º of grid size.

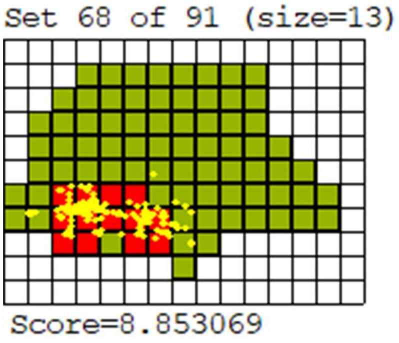

Fig. S130. Area of Endemism (AE = 68) recovered for 0.5º x 0.5º of grid size.

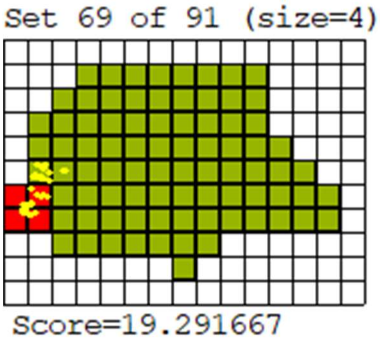

Fig. S131. Area of Endemism (AE = 69) recovered for 0.5º x 0.5º of grid size.

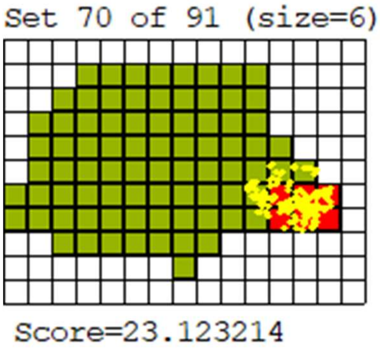

Fig. S132. Area of Endemism (AE = 70) recovered for 0.5º x 0.5º of grid size.

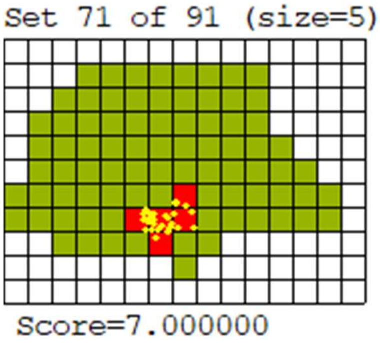

Fig. S133. Area of Endemism (AE = 71) recovered for 0.5º x 0.5º of grid size.

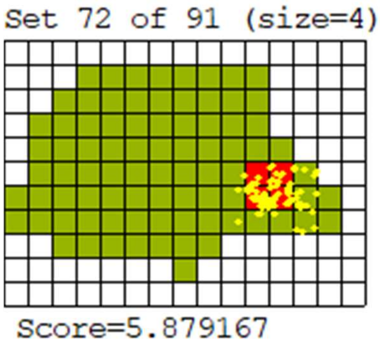

Fig. S134. Area of Endemism (AE = 72) recovered for 0.5º x 0.5º of grid size.

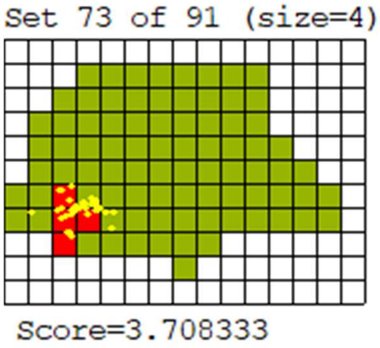

Fig. S135. Area of Endemism (AE = 73) recovered for 0.5º x 0.5º of grid size.

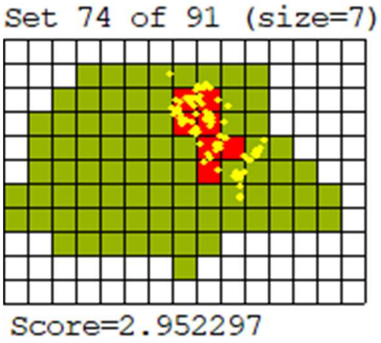

Fig. S136. Area of Endemism (AE = 74) recovered for 0.5º x 0.5º of grid size.

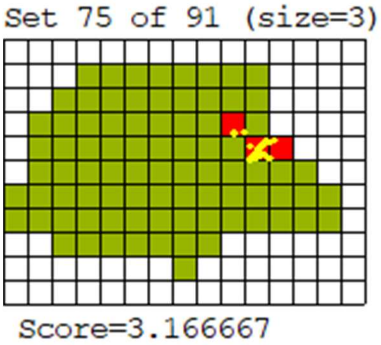

Fig. S137. Area of Endemism (AE = 75) recovered for 0.5º x 0.5º of grid size.

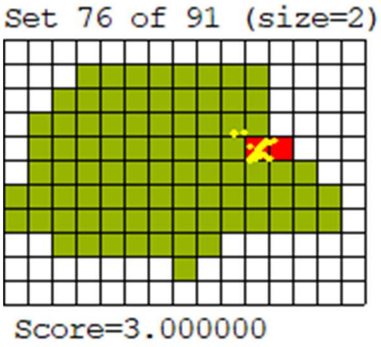

Fig. S138. Area of Endemism (AE = 76) recovered for 0.5º x 0.5º of grid size.

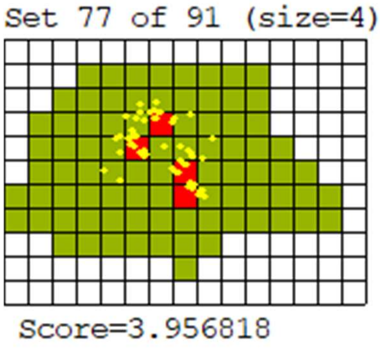

Fig. S139. Area of Endemism (AE = 77) recovered for 0.5º x 0.5º of grid size.

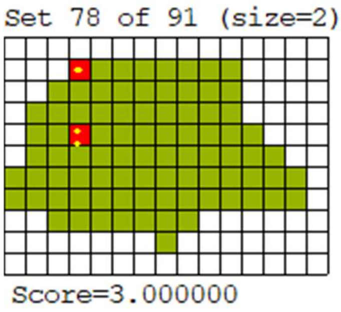

Fig. S140. Area of Endemism (AE = 78) recovered for 0.5º x 0.5º of grid size.

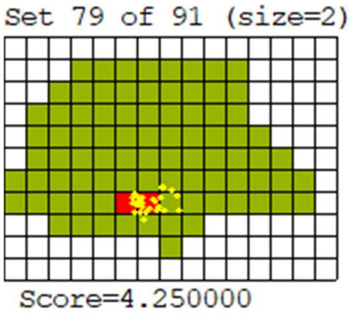

Fig. S141. Area of Endemism (AE = 79) recovered for 0.5º x 0.5º of grid size.

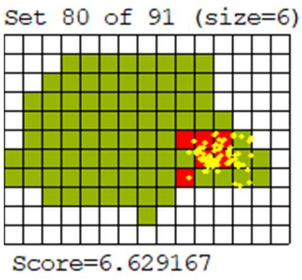

Fig. S142. Area of Endemism (AE = 80) recovered for 0.5º x 0.5º of grid size.

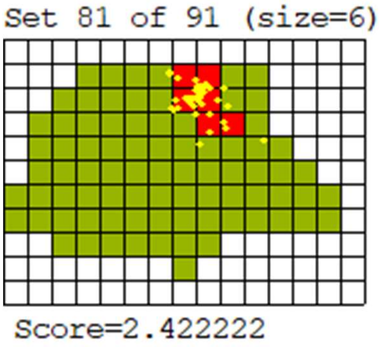

Fig. S143. Area of Endemism (AE = 81) recovered for 0.5º x 0.5º of grid size.

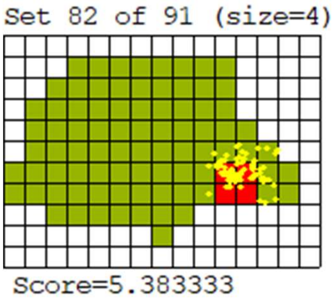

Fig. S144. Area of Endemism (AE = 82) recovered for 0.5º x 0.5º of grid size.

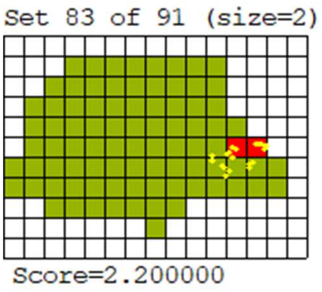

Fig. S145. Area of Endemism (AE = 83) recovered for 0.5º x 0.5º of grid size.

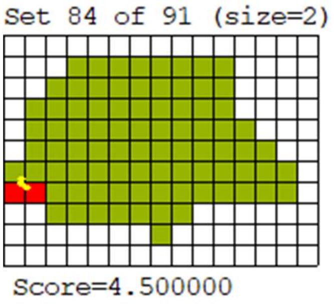

Fig. S146. Area of Endemism (AE = 84) recovered for 0.5º x 0.5º of grid size.

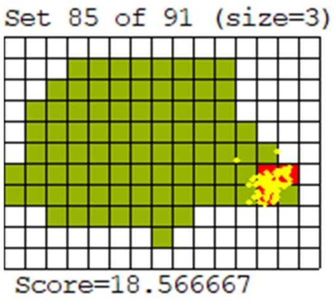

Fig. S147. Area of Endemism (AE = 85) recovered for 0.5º x 0.5º of grid size.

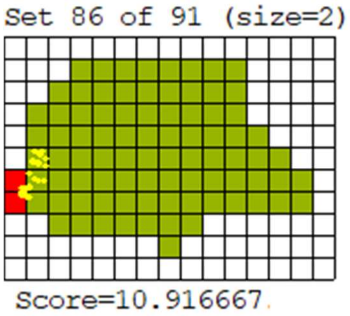

Fig. S148. Area of Endemism (AE = 86) recovered for 0.5º x 0.5º of grid size.

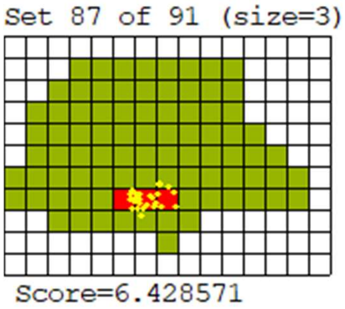

Fig. S149. Area of Endemism (AE = 87) recovered for 0.5º x 0.5º of grid size.

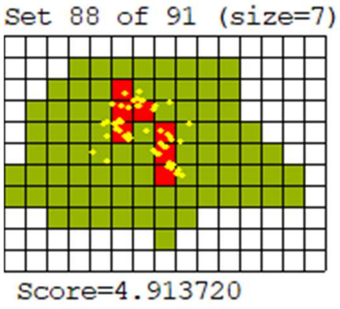

Fig. S150. Area of Endemism (AE = 88) recovered for 0.5º x 0.5º of grid size.

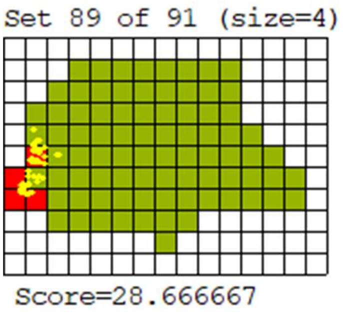

Fig. S151. Area of Endemism (AE = 89) recovered for 0.5º x 0.5º of grid size.

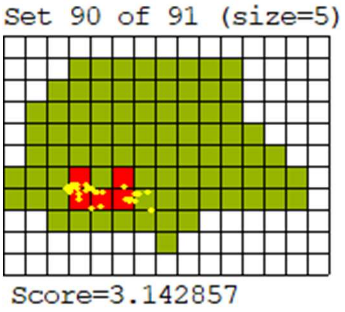

Fig. S152. Area of Endemism (AE = 90) recovered for 0.5º x 0.5º of grid size.

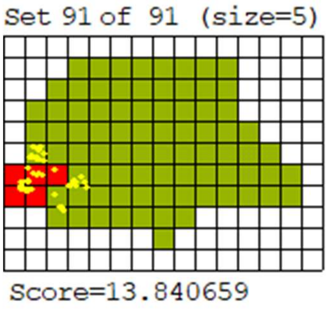

Fig. S153. Area of Endemism (AE = 91) recovered for 0.5º x 0.5º of grid size.
